# Anchor Peptide‐Based Immobilization Strategy Promote the Applications of Pickering Emulsion System in Natural Products Glycosylation

**DOI:** 10.1002/anie.202500834

**Published:** 2025-04-03

**Authors:** Peng Zhang, Zhe Dong, Shuaiqi Meng, Zhongyu Li, Yu Ji, Ulrich Schwaneberg

**Affiliations:** ^1^ Institute of Biotechnology RWTH Aachen University Worringerweg 3 52074 Aachen Germany; ^2^ School of Food Science and Bioengineering Changsha University of Science & Technology Changsha 410114 China; ^3^ State Key Laboratory of Green Biomanufacturing, College of Life Science and Technology Beijing University of Chemical Technology Beijing 100029 China; ^4^ Beijing Advanced Innovation Center for Soft Matter Science and Engineering Beijing University of Chemical Technology Beijing 100029 China

**Keywords:** Anchor peptide, Glycosylation, Immobilization of enzymes, Pickering emulsion, Spy chemistry

## Abstract

Pickering emulsion systems serve as an advanced platform for efficient fine chemical production in biphasic enzymatic catalysis, though their applications are currently limited to a few commercial enzyme classes. Herein, we designed an anchor peptide‐based immobilization strategy for Pickering emulsions to achieve efficient glycosylation of natural products by glycosyltransferases (GTs). Firstly, through enzyme mining, natural GTs were utilized to synthesize pharmaceutically important acacetin glucoside and galactoside. However, the best‐performing enzymes, BacGT and BarGT‐3, still showed low conversions for acacetin glucoside (<40%) and galactoside (<10%). Then, Spy chemistry was employed to cyclize these two GTs (Spy_BacGT and Spy_BarGT‐3) for improved robustness, and a Pickering emulsion was formed with the free two Spy_GTs, using 20% 2‐hexanone and 1% mesoporous silica nanoparticles (carriers of mesoporous, CM), achieving 70% and 66% conversions of acacetin glucoside and galactoside, respectively. Further immobilization of the cyclized GTs onto CM via the anchor peptide liquid chromatography peak I (LCI) (Spy_BacGT/BarGT‐3_LCI@CM) further enabled the system to reach more than 90% conversions of acacetin glycosides, retaining 90% conversions after 6–8 cycles. Moreover, this strategy was applied to GTs exhibiting substrate selectivity, achieving efficient nonselective catalysis. This study provides a simple and efficient immobilization strategy to broaden the applications of the Pickering emulsion system in enzymatic glycosylation.

## Graphical abstract

This study designs an anchor peptide‐based immobilization strategy to expand the application of Pickering emulsion to glycosyltransferases (GTs) for the first time, addressing low conversion challenge in aqueous solutions. The glycosylation Pickering emulsion achieved more than 90% conversion of natural product glycosides with high recyclability, offering a practical approach to broaden the applications of natural enzymes in Pickering emulsion.

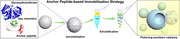

Pickering emulsions, stabilized by solid particles, are surfactant‐free dispersions of two immiscible liquid phases (typically oil and water).^[^ ^1^ ^]^ It emerges as an attractive catalysis platform to surpass the capability of traditional catalysis technology, owing to its unique biphasic environment and compartmentalized droplets.^[^ ^2^ ^]^ However, due to the requirements for suitable biphasic solvents, robust enzymes, and immobilization approaches, the current applications of Pickering emulsion are still largely confined to a few well‐established classes of enzymes (e.g., lipases and benzaldehyde lyase).^[^ ^3^, ^4^ ^]^ Therefore, expanding the application of Pickering emulsion system to more enzyme classes could unlock its untapped potentials in addressing critical production challenges in enzyme catalysis, including enzymatic glycosylation.

Enzymatic glycosylation, which has emerged as an eco‐friendly alternative under ambient conditions, offers significant advantages in terms of optimizing biological activity, enhancing bioavailability, and improving water solubility of substrates,^[^ ^5^, ^6^ ^]^ and it has gained widespread attention in various fields, including pharmaceuticals, food science, and industrial bioprocessing.^[^ ^7^, ^8^ ^]^ Glycosyltransferases (GTs) serve as the primary catalysts in enzyme‐catalyzed glycosylation, facilitating the transfer of sugar moieties from activated sugar donors to target acceptors.^[^ ^9^ ^]^ However, natural GTs usually show limitations in catalytic performance. On one hand, this necessitates enzyme mining and/or protein engineering to meet various synthetic needs.^[^ ^10^, ^11^ ^]^ To date, protein engineering (e.g., directed evolution and rational design) remains the main strategy for enhancing enzyme catalytic efficiency and robustness,^[^ ^12^, ^13^ ^]^ although it still requires extensive effort and time and often faces trade‐offs between activity and stability.^[^ ^14^ ^]^ On the other hand, mass‐transfer efficiency is also a pressing challenge, as the substrates often exhibit hydrophobicity. Organic solvents can improve mass‐transfer efficiency, but the engineering of GTs for organic solvent resistance is unlikely to dramatically increase catalytic activity.^[^ ^15^ ^]^ This motivates us to employ a Pickering emulsion system for efficient glycosylation reactions with less effort, but two challenges should be addressed: high robustness and efficient immobilization of GTs.

Improving enzyme robustness is essential for a successful Pickering emulsion, and the SpyTag/SpyCatcher conjugation system offers a promising solution for this improvement. This system is constructed based on the chemical interactions of a split protein domain, CnaB2 (*Streptococcus pyogenes*), consisting of two peptide fragments named; SpyTag (13 amino acids) and SpyCatcher (116 amino acids).^[^ ^16^ ^]^ The Spy chemistry facilitates the spontaneous reconstitution of covalent conjugation between SpyTag and SpyCatcher (Asp‐Lys) for enzyme cyclization and led to a much larger increase in robustness.^[^ ^17^ ^]^ Schoene et al. reported that β‐lactamase (BLA) was cyclized using the SpyTag/SpyCatcher conjugation system and retained activity even after heating at 100 °C.^[^ ^18^ ^]^ This highlights the potentials of Spy chemistry to significantly enhance the robustness of enzymes.

More importantly, enzyme immobilization on carriers plays a pivotal role in Pickering emulsion.^[^ ^19^ ^]^ Traditional enzyme immobilization is often achieved by modifying the surface of materials (e.g., nanoparticles) to enhance adsorption between the enzyme and the carriers.^[^ ^20^, ^21^ ^]^ Anchor peptides (APs), also known as material‐binding peptides (MBPs), offer a versatile platform as short peptides capable of binding to a wide range of material surfaces,^[^ ^22^ ^],^ e.g., silica, polymer, and metal.^[^ ^23^ ^]^ Especially, anchor peptide liquid chromatography peak I (LCI) exhibits promiscuous and efficient binding to many material surfaces under ambient conditions, such as stainless steel,^[^ ^24^ ^]^ synthetic polymers,^[^ ^25^ ^]^ and surfaces of plant leaves.^[^ ^26^ ^]^ APs can provide a simple, efficient, and economical strategy to facilitate the enzyme immobilization onto solid supports or surfaces without any modification.

In this study, we integrated an anchor peptide‐based immobilization strategy into Pickering emulsion system for enzymatic glycosylation (Figure 1a), and achieved efficient production of the pharmaceutically valuable acacetin glycosides (acacetin 7‐*O*‐β‐D glucoside, aca‐Glc and acacetin 7‐*O*‐β‐D galactoside, aca‐Gal, Figure 1b,c). Aca‐Glc and aca‐Gal are active flavonoid glycosides with diverse bioactivities. Aca‐Glc exhibits potential anti‐hypertensive, myocardial‐protective, anti‐diabetic, anti‐hyperlipidemic, anti‐inflammatory, and antioxidant effects, while aca‐Gal even showed notable bioactivities in the treatment of Alzheimer's disease and human immunodeficiency virus (HIV).^[^ ^27^, ^28^, ^29^, ^30^ ^]^ Currently, acacetin glycosides are mainly extracted from plants, and the in vitro synthesis process remains challenging due to the lack of efficient GTs and the low mass‐transfer efficiency caused by the water insolubility of acacetin.^[^ ^31^, ^32^ ^]^ Therefore, to identify GTs capable of efficiently catalyzing acacetin, we initially verified the previously identified promiscuous GT, BarGT‐3, located in the nBGT3 branch, which displayed notable activity toward flavonoid scaffolds.^[^ ^33^ ^]^ The results showed that the synthesis efficiency of aca‐Glc and aca‐Gal by BarGT‐3 still remained low (less than 25% and 10%, respectively), even with a four‐fold sugar donors supply (Figure 1b,c). Thus, we investigated other potentially efficient GTs within the nBGT‐3 branch. Phylogenetic analysis revealed that the nBGT‐3 branch was mainly divided into five distinct sub‐branches, indicating the existence of GTs with diverse catalytic characteristics (Figure S1). By clustering the GTs of the nBGT‐3 branch with 80% identity using CD‐HIT, we observed that they also grouped into five clusters. Interestingly, apart from BarGT‐3 in sub‐branch 1, the representative GTs from the other four clusters are distributed in the respective four sub‐branches, which are BpsGT (sub‐branch 2), BcyGT (sub‐branch 3), BacGT (sub‐branch 4), and BceGT (sub‐branch 5, Figure S1). The sequence identity among the five representative GTs ranges from 50% to 75% (Table S1).

**Figure 1:**
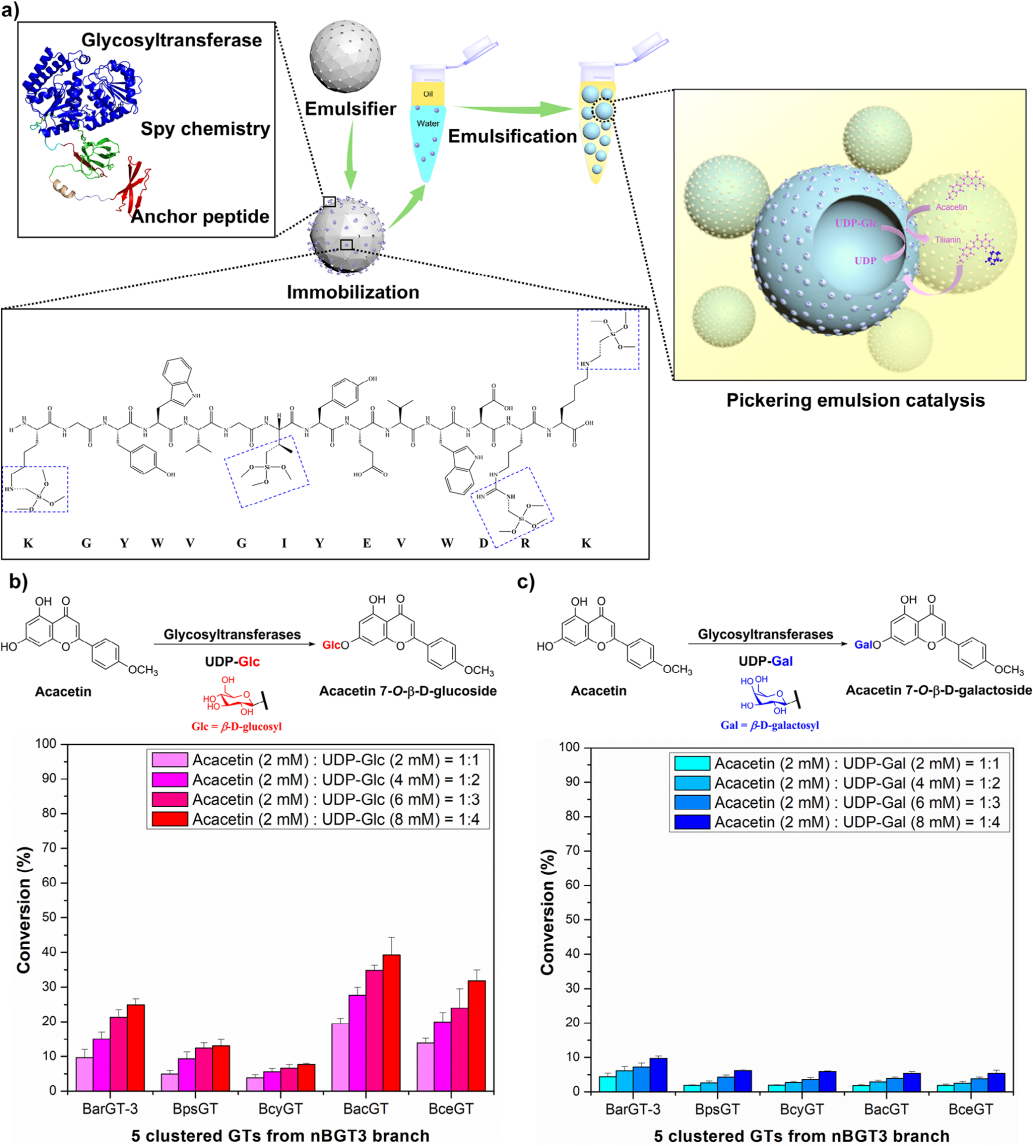
a) The scheme of the integration of the anchor peptide‐based immobilization strategy with Pickering emulsion system for enzymatic glycosylation and the interactions of the anchor peptide with silica particles, potentially facilitated by electrostatic interactions, hydrogen bonding, and hydrophobic effects; b) The glycosylation conversions of GTs BarGT‐3, BpsGT, BcyGT, BacGT, and BceGT for substrate acacetin with different concentrations of sugar donor UDP‐glucose (UDP‐Glc); c) The glycosylation conversions of GTs BarGT‐3, BpsGT, BcyGT, BacGT, and BceGT for acacetin with different concentrations of UDP‐galactose (UDP‐Gal).

All the five representative GTs (BarGT‐3, BpsGT, BcyGT, BacGT, and BceGT) achieved soluble expression (Figure S2). The glycosylation assays revealed that BacGT and BarGT‐3 exhibited the highest activity for synthesizing aca‐Glc and aca‐Gal, yet their conversions remained relatively low (less than 40% and 10%, respectively, Figure 1b,c and Figures S3,S4). Therefore, these two GTs were selected as candidates for utilization in the Pickering emulsion system. The first step is to explore oil phases immiscible with water phase but can dissolve water‐insoluble substrates (e.g., acacetin) to improve mass transfer efficiency. For this purpose, 20% (vol%) long‐chain alcohols and ketones (6–10 carbons) were selected for the glycosylation assays with mixed oil–water phases (ratio of substrate to sugar donor: 1:2). The alcohols, including 2‐hexanol, 2‐octanol, and 2‐decanol with different logP values significantly decrease enzyme activity (<10%, Figure S5). Interestingly, ketones such as 2‐hexanone can significantly enhance enzyme activity. The addition of 20% 2‐hexanone increased the conversions of BacGT and BarGT‐3 (up to 51.5% and 51.3%, respectively, Figure S5). This improvement can be attributed to the lower hydrophilicity of 2‐hexanone compared to alcohols with hydroxyl groups, which reduces its ability to penetrate and disrupt the hydrophobic active pocket of enzymes. However, as the chain length (logP value) increased, the addition of ketones is detrimental to enzyme activity: the addition of 2‐octanone only maintained weak activities of all five GTs (<30%), while the addition of 2‐decanone damaged their activities (<10%, Figure S5). Given these results, 2‐hexanone was selected to further test the conversions of BacGT and BarGT‐3 in oil–water biphasic reaction system, and Spy chemistry was used to enhance the robustness of the GTs (Figure 2a). By adding different volume concentrations (10%‐60%, vol%) of 2‐hexanone, it was observed that 20% 2‐hexanone maintained the highest conversions of BacGT and BarGT‐3 (Figure 2b,c). However, as the concentration of 2‐hexanone increased, the activities of the two GTs gradually decreased (Figure 2b,c). To further enhance the robustness of BacGT and BarGT‐3, we used the SpyTag/SpyCatcher conjugation system to cyclize BacGT and BarGT‐3. SpyTag003 was fused to the N‐terminus of BacGT and SpyCatcher003 to the C‐terminus (Using Spy_BacGT as an example, Figure 2a). To further confirm the successful cyclization of BacGT and BarGT‐3, we introduced a mutation (D10A) in SpyTag003, and the cyclized form of Spy_GTs showed lower mobility compared to the linear form of SpyD10A_GTs in SDS‐PAGE gel (Figure S2), consistent with previous studies.^[^ ^18^ ^]^ To evaluate the effects of cyclization on the robustness of BacGT and BarGT‐3, the conversions of Spy_BacGT and Spy_BarGT‐3 was measured at different volume concentrations (10%‐60%, vol%) of 2‐hexanone in biphasic reaction system. The results showed that Spy_BacGT and Spy_BarGT‐3 exhibited the highest activities in a biphasic system with 20% 2‐hexanone, and maintained more than 50% and 40% conversions with 60% 2‐hexanone, respectively (Figure 2b,c). It is indicated that Spy chemistry can effectively enhance the robustness of the GTs. Therefore, the Pickering emulsion system was further introduced to promote enzymatic glycosylation.

**Figure 2:**
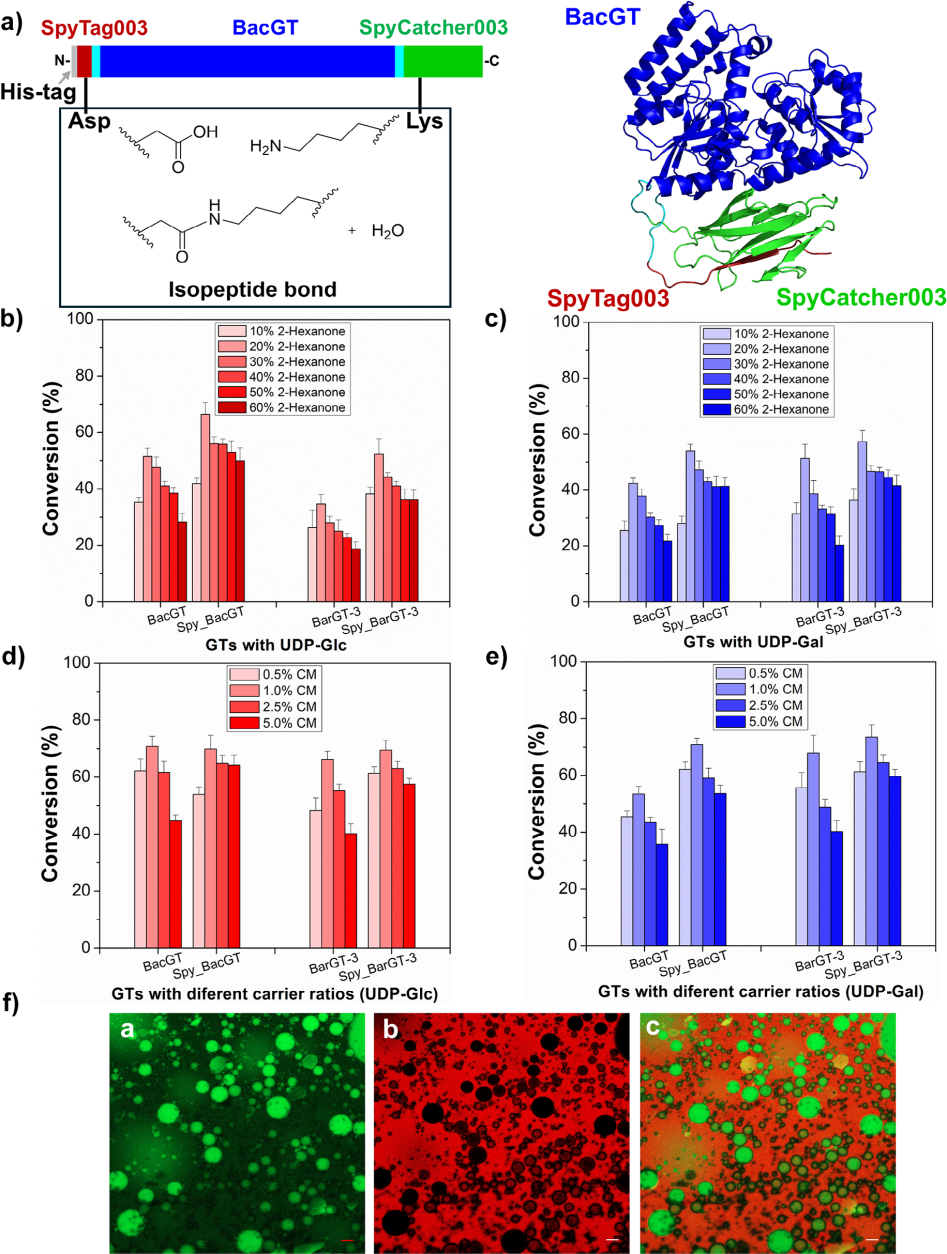
a) The schematic diagram of the SpyTag/SpyCatcher conjugation system used to cyclize BacGT to form Spy_BacGT; b) Comparison of the conversions of BacGT and Spy_BacGT, BarGT‐3 and Spy_BarGT‐3 with different 2‐hexanone concentrations (10%–60%) with sugar donor UDP‐Glc; c) Comparison of the conversions of BacGT and Spy_BacGT, BarGT‐3 and Spy_BarGT‐3 with different 2‐hexanone concentrations (10%–60%) with sugar donor UDP‐Gal; d) Comparison of the conversions of BacGT and Spy_BacGT, BarGT‐3 and Spy_BarGT‐3 with different carriers of mesoporous (CM) concentrations (0.5%–5%, wt%) with sugar donor UDP‐Glc; e) Comparison of the conversions of BacGT and Spy_BacGT, BarGT‐3 and Spy_BarGT‐3 with different CM concentrations (0.5%‐5%, wt%) with sugar donor UDP‐Gal; f) Confocal laser scanning microscopy (CLSM) images of the droplets dyed with a) Fluorescein isothiocyanate (FITC), water soluble, and b) Nile red, oil soluble/organic phase (2‐hexanone), and c) an individual water‐in‐oil Pickering emulsion.

To design an efficient Pickering emulsion system, mesoporous silica nanoparticles (carriers of mesoporous, CM) were selected as emulsifiers.^[^ ^34^ ^]^ CM possesses a glossy surface with an average diameter of 120 nm was observed in the scanning electron microscopy (SEM) image and the well‐defined pore channel is observed by transmission electron microscopy (TEM), indicating that CM could serve as a promising carrier for immobilization and mass transport of substrate (Figure S6). The porosity of CM was confirmed by N_2_ sorption isotherms (77 K), achieving a Brunauer–Emmett–Teller (BET) surface area of 881.6 m^2^ g−^1^. The average pore size distribution of CM is around 8 nm (Figure S7). These results indicate that CM could provide sufficient space for immoblization of GTs and transportation of substrates. The water contact angel was 66°, implying that the amphiphilic nanoparticle was likely to be the emulsifier for Pickering emulsion (Figure S8). Further, the effects of different concentrations of CM (1%‐5%, wt%) on the catalytic activity of GTs and Spy_GTs were explored. The results showed that both GTs and Spy_GTs achieved their highest conversions with 1% CM. Notably, Spy_BacGT and Spy_BarGT‐3 maintained more than 50% conversion even with 5% CM (Figure 2d,e). To further investigate whether the oil–water biphasic system formed a Pickering system by CM, the droplets were dyed with (FITC, for the water phase) and nile red (for the oil phase). As CLSM images shown in Figure 2f, this emulsion was in the form of water‐in‐oil, and herein Pickering emulsion droplet are uniform in shape with the average diameter of 8 µm.^[^ ^35^, ^36^ ^]^ Overall, Spy_BacGT and Spy_BarGT‐3 maintained high catalytic activity even at increasing CM concentrations, outperforming native BacGT and BarGT‐3. CM proved to be an effective emulsifier, forming a stable and efficient Pickering emulsion system.

Furthermore, anchor peptide LCI was utilized to immobilize Spy_BacGT and Spy_BarGT‐3 on CM. Based on the high conversions of the free Spy_GTs in the Pickering emulsion system, we fused LCI to the C‐terminus of Spy_GTs (Figure 3a) to achieve better immobilization, as proteins with C‐terminally fused LCI typically show strong binding.^[^ ^22^ ^]^ The anchor peptide absorbed and self‐assembled with silica particles possible via electrostatic interactions, hydrogen bonding, and hydrophobic effects (Figure 1a).^[^ ^37^, ^38^ ^]^ To confirm the successful cyclization of Spy_GTs_LCI, negative controls were also generated (SpyDA_GTs_LCI). The SDS‐PAGE gel showed that the cyclized form of Spy_BacGT/BarGT‐3_LCI had lower mobility, indicating the efficient cyclization (Figure S2). To investigate the immobilization efficiency of BacGT, Spy_BacGT, and Spy_BacGT_LCI on CM. BacGT and its fusion proteins were co‐incubated with CM and the efficiency of immobilization were detected after 30 min. The results showed that the immobilization rates for BacGT and Spy_BacGT were only 1.1 × 10^−9^ mol mg−^1^ and 1.2 × 10^−9^ mol mg−^1^, respectively. Notably, Spy_BacGT_LCI achieved an immobilization rate of 3.7 × 10^−9^ mol mg−^1^, which is 3.4‐fold higher than BacGT (Figure 3b). The water contact angle also decreased from 66° to 58° after immobilization, reflecting an improvement in the hydrophilicity of the carrier surface (Figure S8). To visualize the immobilization performance of Spy_BacGT_LCI, we labeled it with FITC and mixed it with CM (Spy_BacGT_LCI@CM). For comparison, carriers CM with FITC treatment were examined to exclude the possible fluorescent signal from particles themselves. CLSM images revealed that the green fluorescence of FITC‐Spy_BacGT_LCI@CM were obvious and homogeneous, indicating that Spy_BacGT_LCI was fully adsorbed on the carriers CM, while no obvious green signal was observed in only carriers (Figure 3c). The Spy_BacGT_LCI@CM and Spy_BarGT‐3_LCI@CM (the molar equivalent to free Spy_GTs) further achieved the highest conversion (>90%) with 30% 2‐hexanone, and even retained more than 70% conversion with 60% 2‐hexanone (Figure 3d). Additionally, we examined mass‐transfer during the entire catalytic process. Before the addition of Spy_BacGT_LCI@CM, 98% (∼1.96 mM) of the substrate was in the oil phase (2‐hexanone). Throughout the catalysis process, the substrate distribution in the aqueous phase remained less than 5%. However, the product distribution in oil and water phases were 62% and 33% after 2 h, respectively (Figure S9). This indicates that in the biphasic system, the glycosylated product will be gradually transferred from the oil phase to the water phase, thereby enhancing mass transfer efficiency during the process of Pickering emulsion catalysis. After the initial reaction, the system was centrifuged, and the Spy_BacGT_LCI@CM and Spy_BarGT‐3_LCI@CM were directly recycled for the next reaction cycle to explore the recyclability of the Pickering emulsion catalysis system. The two immobilized GTs demonstrated excellent recyclability, retaining 90% of their activity even after 6–8 cycles (Figure 3e,f). It is shown that a stable Pickering system could still be formed in the 8^th^ reaction cycle of Spy_BacGT_LCI@CM with UDP‐Glc. The Pickering emulsion droplets exhibited uniform shapes, but their average diameter increased (Figure S10). We also observed that the conversions of the Pickering emulsion system decreased with increasing cycles, which might result from the loss of enzymes. To ascertain this hypothesis, the mixture in the 8^th^ reaction cycle of Spy_BacGT_LCI@CM with UDP‐Glc was washed with buffer and the washed buffer was determined by circular dichroism. The adsorption in the range from 190 to 260 nm implied the presence of a few enzymes in the buffer (Figure S11). The acacetin glycosides catalyzed by the Pickering emulsion were isolated and confirmed by ^1^H and ^13^C Nuclear Magnetic Resonance (NMR) spectra, and the glycosylation at hydroxy groups of C‐1 of acacetin glycosides were further determined by the key Heteronuclear Multiple Bond Correlations (HMBC) from H‐1 to C‐1, respectively.(Figures S12–S17).

**Figure 3:**
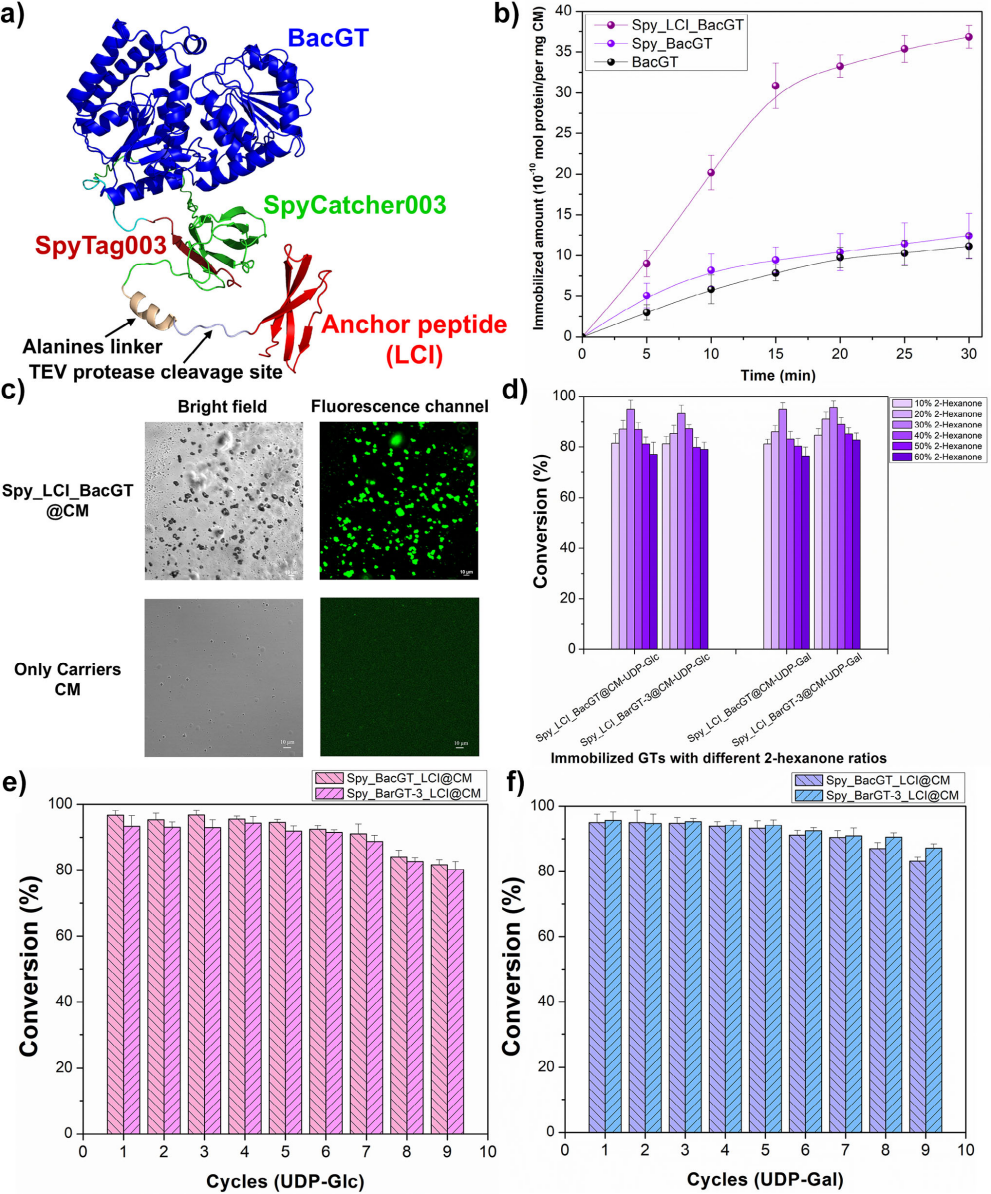
a) The schematic diagram of the LCI was fused into Spy_BacGT to form Spy_BacGT_LCI; b) The efficiency of immobilization for BacGT and its fusion proteins to CM within 30 min; c) The CLSM images of the immobilization performance of Spy_BacGT_LCI@CM (labeled with FITC) with control (only carriers CM labeled with FITC); d) Comparison of the conversions of Spy_BacGT_LCI@CM and Spy_BarGT‐3_LCI@CM with different 2‐hexanone concentrations (10%–60%) with sugar donors UDP‐Glc and UDP‐Gal; e) The conversions of Spy_BacGT_LCI@CM and Spy_BarGT‐3_LCI@CM at different Pickering emulsion reaction cycles (1–9) with sugar donor UDP‐Glc; f) The conversions of Spy_BacGT_LCI@CM and Spy_BarGT‐3_LCI@CM at different Pickering emulsion reaction cycles (1–9) with sugar donor UDP‐Gal.

Moreover, we utilized the Pickering emulsion system for another GT, BarGT‐1, known for its substrate selectivity. Our previous studies reported that BarGT‐1 exhibits selectivity toward different hydroxyl groups within the same scaffold.^[^ ^39^ ^]^ We designed an immobilized enzyme, Spy_BarGT‐1_LCI@CM to evaluate the universality of the anchor peptide‐based immobilization strategy in enhancing the catalytic efficiency of BarGT‐1 using substrate spectrum of six substrates, with the corresponding glycosylated products already identified (Figure 4).^[^ ^39^ ^]^ The results showed that, compared to the free enzyme BarGT‐1, the immobilized enzyme Spy_BarGT‐1_LCI@CM exhibited nonselective and highly efficient glycosylation of different hydroxyl positions on a single scaffold, as well as promoted promiscuous and efficient glycosylation across different chemical skeletons (Figure 4). Additionally, the immobilized enzyme Spy_BarGT‐3_LCI@CM also exhibited superior catalytic activities compared to the free enzyme BarGT‐3. Both Spy_BarGT‐1_LCI@CM and Spy_BarGT‐3_LCI@CM achieved substrate conversion rates exceeding 90% for various substrates, highlighting the effectiveness of the anchor peptide‐based immobilization strategy in enhancing enzymatic performance and broadening the applicability of GTs to diverse catalytic scenarios.

**Figure 4:**
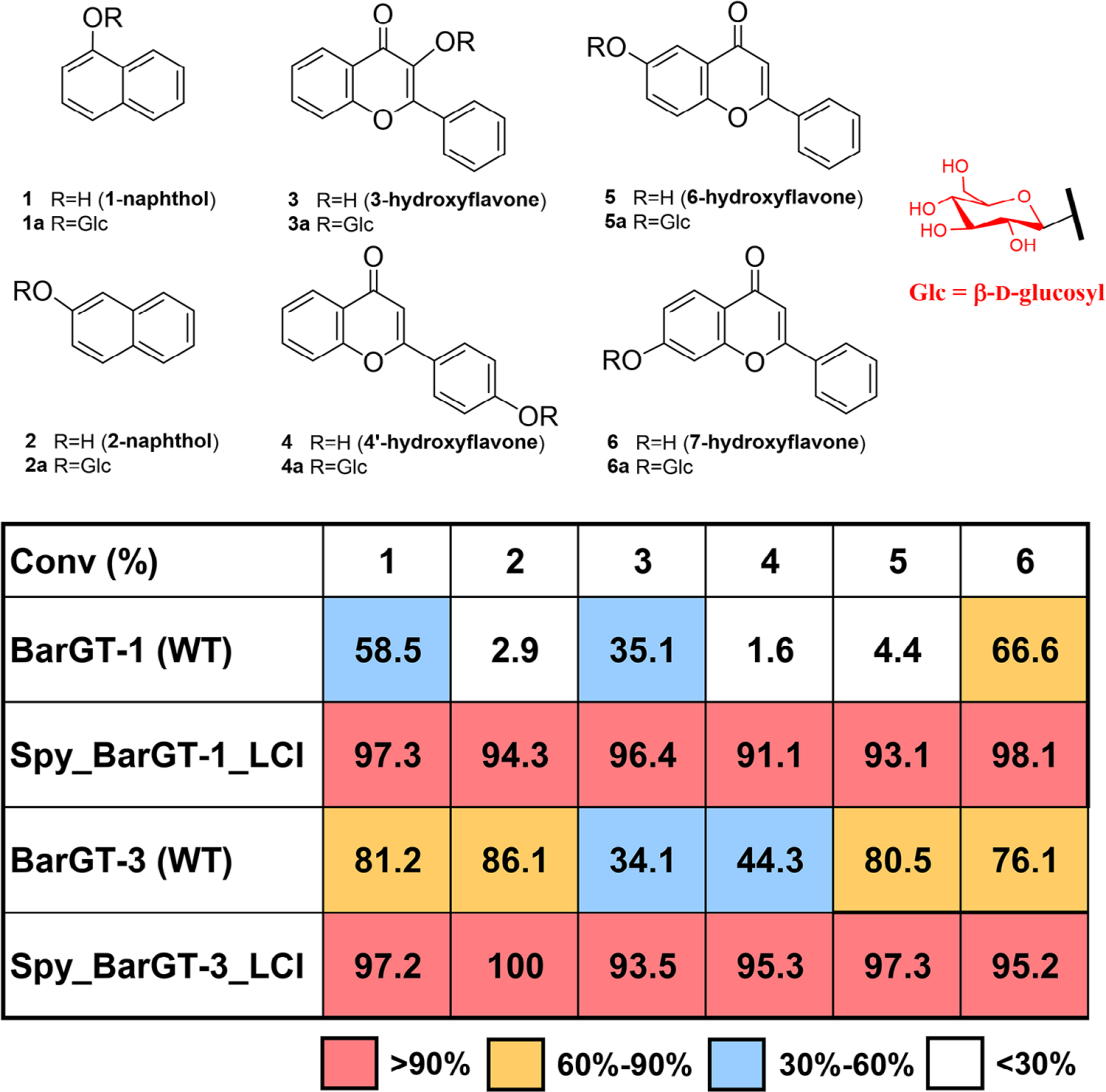
The conversions of BarGT‐1, BarGT‐3, Spy_BarGT‐1_LCI@CM, and Spy_BarGT‐3_LCI@CM towards a panel of 6 substrates **1**–**6**: 1‐naphthol **1**, 2‐naphthol **2**, 3‐hydroxyflavone **3**, 4′‐hydroxyflavone **4**, 6‐hydroxyflavone **5**, 7‐hydroxyflavone **6**, are shown in a heatmap. The glycosylated products are **1a**–**6a**: 1‐naphthol *O*‐β‐D glucoside **1a**, 2‐naphthol *O*‐β‐D glucoside **2a**, 3‐hydroxyflavone *O*‐β‐D glucoside **3a**, 4′‐hydroxyflavone *O*‐β‐D glucoside **4a**, 6‐hydroxyflavone *O*‐β‐D glucoside **5a**, 7‐hydroxyflavone *O*‐β‐D glucoside **6a**.

In conclusion, this study, for the first time, established a highly efficient Pickering emulsion system for enzymatic glycosylation. The integration of the Pickering emulsion system with an anchor peptide immobilization strategy on stabilized GTs successfully overcame the mass‐transfer limitations typically encountered in the glycosylation of poorly water‐soluble substrates, achieving remarkable conversion. The integration of the Spy chemistry with anchor peptide‐based immobilization provides a simple and efficient platform for enhancing enzyme robustness and catalytic performance. This study not only expands the use of Pickering emulsions in glycosylation, particularly for hydrophobic pharmaceutical substrates, but also opens new avenues for adapting Pickering emulsion systems to other enzyme classes, further broadening their potential applications in biocatalysis and the production of high‐value products.

## Supporting Information

The authors have cited additional references within the Supporting Information.^[^ ^34^, ^39^, ^40^, ^41^, ^42^ ^]^

## Conflict of Interests

The authors declare no conflict of interest.

## Supporting information



Supporting Information

## Data Availability

The data that support the findings of this study are available from the corresponding author upon reasonable request.
