# Supplementary material for: Anchor Peptide‐Based Immobilization Strategy Promote the Applications of Pickering Emulsion System in Natural Products Glycosylation
Source: Angew Chem Int Ed Engl. 2025 Apr 3;64(21):e202500834. doi: 10.1002/anie.202500834 (PMC12087810; doi:10.1002/anie.202500834)
Supplement: Supplementary file 1 — Supporting Information [file ANIE-64-e202500834-s001.docx]

**Anchor Peptide-based Immobilization Strategy Promote the Applications of Pickering Emulsion System in Natural Product Glycosylation**

Peng Zhang^+^,^[1]^ Zhe Dong^+^,^[1,4]^ Shuaiqi Meng,^[1]^ Zhongyu Li,^[1]^ Yu Ji*,^[1,2]^ and Ulrich Schwaneberg*,^[1,3]^

[1] Dr. P. Zhang, Dr. Z. Dong, Dr. S. Meng, Z. Li, Dr. Y. Ji, Prof. U. Schwaneberg
Institute of Biotechnology
RWTH Aachen University
Worringerweg 3, 52074 Aachen, Germany

[2] Prof. Dr. Y. Ji

State Key Laboratory of Green Bio-manufacturing, College of Life Science and Technology

Beijing University of Chemical Technology

100029 Beijing, China

[3] Prof. U. Schwaneberg

Beijing Advanced Innovation Center for Soft Matter Science and Engineering

Beijing University of Chemical Technology

100029 Beijing, China

[4] Dr. Z. Dong

School of Food Science and Bioengineering

Changsha University of Science & Technology

410114 Changsha, China

^+^These authors contributed equally to this work.

*Corresponding authors

u.schwaneberg@biotec.rwth-aachen.de, yu.ji@buct.edu.cn,

**Table of Contents**

[1. Experimental Procedures 3](#_Toc189671317)

[1.1. Phylogenetic analysis 3](#_Toc189671318)

[1.2. Chemicals and reagents 3](#_Toc189671319)

[1.3. Bacterial strains, plasmids and media 3](#_Toc189671320)

[1.4. Protein expression and purification 3](#_Toc189671321)

[1.5. Synthesis of mesoporous silica nano particles 4](#_Toc189671322)

[1.6. Characterization of materials and emulsion 4](#_Toc189671323)

[1.7. Determination of enzyme immobilization rate 5](#_Toc189671324)

[1.8. *In vitro* glycosylation reactions 5](#_Toc189671325)

[1.9. HPLC-PDA, MS and NMR assays 6](#_Toc189671326)

[1.10. Mass transfer assays for substrate/product 7](#_Toc189671327)

[1.11. Characterization of circular dichroism 7](#_Toc189671328)

[2. Additional Figures and Tables 8](#_Toc189671329)

[Fig. S1. Phylogenetic analysis of GTs in the nBGT3 branch and selection of representative enzymes in sub-branches through CD-HIT clustering 8](#_Toc189671330)

[Fig. S2. The SDS-PAGE detection of the purification of GT proteins 9](#_Toc189671331)

[Fig. S3. HPLC and positive ion mass spectrometry (MS) analysis for product aca-Glc 10](#_Toc189671332)

[Fig. S4. HPLC and positive ion mass spectrometry (MS) analysis for product aca-Gal 11](#_Toc189671333)

[Fig. S5. 20% (volume concentration, vol%) long-chain carbon alcohols and ketones (6 to 10 carbons) were selected for the biphasic glycosylation assays 12](#_Toc189671334)

[Fig. S6. The images of scanning electron microscope (SEM) and transmission electron microscope (TEM) of representative carriers of mesoporous (CM) 13](#_Toc189671335)

[Fig. S7. Nitrogen adsorption/desorption isotherms and pore diameter distributions of carriers of mesoporous (CM) 14](#_Toc189671336)

[Fig. S8. The air-water contact angle for CM before and after enzyme immobilization 15](#_Toc189671337)

[Fig. S9. The proportions of the substrate (acacetin) and product (aca-Glc) in the oil phase (2-hexanone) and water phase over 2 h 16](#_Toc189671338)

[Fig. S10. The Confocal laser scanning microscopy (CLSM) images of the droplets 17](#_Toc189671339)

[Fig. S11. Circular dichroism spectra of the washed buffer from the 8^th^ reaction cycle 18](#_Toc189671340)

[Table S1. Sequence identity among the five GT candidates 19](#_Toc189671341)

[3. ^1^H and ^13^C NMR spectral data of glycosylated products 20](#_Toc189671342)

[Fig. S37. The integration of Spy chemistry with anchor peptide-based immobilization for enhancing enzyme robustness and catalytic performance in three phases 36](#_Toc189671343)

[4. The DNA and protein sequences of GTs with restriction sites 37](#_Toc189671344)

[5. References 48](#_Toc189671345)

# 1. Experimental Procedures

## 1.1. Phylogenetic analysis

A total of 273 GT sequences from nBGT-3 branch were conducted multiple sequence alignments using MAFFT (https://mafft.cbrc.jp/alignment/server/), after removing redundant sequences.^[1]^ MrBayes (version 3.2) were used for phylogenetic analysis. MrBayes is a software tool that performs phylogenetic analysis using Bayesian inference and Markov Chain Monte Carlo algorithms.^[2]^ The resulting phylogenetic trees were visualized by iTOL.^[3]^

## 1.2. Chemicals and reagents

All chemicals were of analytical grade and purchased from Sigma-Aldrich (Steinheim, Germany), AppliChem (Darmstadt, Germany), ExtraSynthese (Genay Cedex, France), TCI (Eschborn, Germany), Cymit (Barcelona, Spain), and VWR International (Darmstadt, Germany).

## 1.3. Bacterial strains, plasmids and media

Electrocompetent *E. coli* BL21 (DE3) (Agilent Technologies, Santa Clara, CA) was prepared for the protein expression. The genes encoding BarGT-3 (QEL68377), BpsGT (AJI18252), BcyGT (AWC60795), BacGT (AIE79969), BceGT (QKH10521) and BarGT-1 (QEL68904) with pET-28a (+) vector were synthesized in GenScript (GenScript Biotech, Rijswijk, Netherlands). LB media: 10 g/L tryptone, 5 g/L yeast extract and 10 g/L NaCl; SOC media: 20 g/L tryptone, 5 g/L yeast extract, 10 mM NaCl, 2.5 mM KCl, 10 mM MgCI_2_, 10 mM MgSO_4_ and 20 mM glucose (Carl Roth GmbH, Karlsruhe, Germany).

## 1.4. Protein expression and purification

As reported in previous protocol,^[4]^ 2 mL seeding transformants (BarGT-3, BpsGT, BcyGT, BacGT, BceGT, BarGT-1, Spy_BacGT and Spy_BacGT_LCI, Spy_BarGT-3 and Spy_BarGT-3_LCI, Spy_BarGT-1 and Spy_BarGT-1_LCI, the primers for variants SpyDA_GT and SpyDA_GT_LCI are:

SpyTagD10A_GT-F: GATGGTT**GCA**GCGTACAAGCGTTATAAAGGATCTG

SpyTagD10A_GT-R: TGTACGC**TGC**AACCATCACGATATGTGGTACACC)

were transferred into a 300 mL flask containing 100 mL LB media (kanamycin, 40 μg/mL) for large-scale cultivation at 37°C until OD reached 0.4~0.6 and induced by adding IPTG (0.1 mM) for protein expression at 16°C for extra 20 h. The cell pellets were harvested by centrifugation at 4000 rpm for 30 min, washed twice with a Tris–HCl buffer (50 mM Tris–HCl, pH 7.5) and resuspended in 10 mL of the same buffer. The cells were sonicated in ice-water mixture (10 min, 5 s pulse, 10 s pause, 60% amplitude), and the supernatant was collected by centrifugation at 4000 rpm for 30 min at 4°C. The target proteins were further analyzed by the 12% sodium dodecyl sulfate-polyacrylamide gel electrophoresis (SDS-PAGE).

After being treated with 0.45 μm filters, the supernatants were purified using a His-Tag Ni-IDA column (MACHEREY-NAGEL, Dueren, Germany). The binding proteins were eluted with a Tris–HCl buffer (50 mM Tris–HCl, pH 7.5) containing different concentrations of imidazole (20 mM, 50 mM, 100 mM, 150 mM and 200 mM), and the elutes were analyzed by SDS-PAGE. Imidazole was removed from the protein solution by using ultrafiltration tubes (30 or 50 kDa, Millipore, Schwalbach, Germany). The concentrated protein samples were stored at −80°C for the assays of purified proteins.

## 1.5. Synthesis of mesoporous silica nano particles

The preparation of the carriers (CM) is based on the previous reference with some modifications.^[5]^ Firstly, 2 g of cetyltrimethylammonium bromide was dissolved in 320 mL of deionized water in a round-bottom flask at the temperature of 35°C. Subsequently, 14 mL of 28 wt% ammonium hydroxide was added in, and the mixture was stirred at 200 rpm for 5 min to obtain a clear solution. Then, a mixture of 10 mL n-hexane and 40 mL tetraethyl orthosilicate was prepared and added dropwise to the above clear solution at a rate of 1 mL/min. The reaction mixture was stirred continuously for 12 h. Finally, the mixture was centrifuged at 8000 rpm for 10 min to collect the solid particles. The powder was washed five times each with 100 mL deionized water and ethanol, and then dried in an oven at 60°C for 8 h. To remove the template, the resultant dried powder was placed in a muffle furnace, heated to 550°C for 6 h at a rate of 15°C/min.

## 1.6. Characterization of materials and emulsion

Before observation of scanning electron microscopy (SEM) and transmission electron microscopy (TEM), the nanoparticles were dispersed in ethanol. SEM images were gained by SU8010 (Hitachi, Japan). TEM observation was conducted on FEI-Talos F200X) (Thermo Scientific, USA). The gas adsorption isotherms were performed on ASAP 2020 (Micromeritics, USA). The N_2_ sorption isotherms were collected at 77 K with a liquid N_2_ bath. The total surface area was calculated with Brunauer−Emmett−Teller (BET) method.

The confocal laser scanning microscopy (CLSM) were observed on Nikon N-SIM S (Nikon, Japan). For confirmation of successful enzyme immobilization on nanoparticles, the enzyme solution was mixed with fluorescein isothiocyanate (FITC) to gain the labelled enzyme. The immobilization was conducted with this labelled enzyme. The resultant immobilized enzyme was observed with an excitation wavelength of 488 nm. The Pickering emulsion was labeled with FITC and Nile red (0.05 mg/mL) to figure out the type of emulsion.

## 1.7. Determination of enzyme immobilization rate

500 µL purified BacGT, Spy_BacGT and Spy_BacGT_LCI (1 mM) were incubated with 5 mg CM with 1000 rpm at room temperature. Samples were taken at 5, 10, 15, 20, 25, and 30 minutes, with three replicates for each time point. The samples were centrifuged continuously at 14,000 rpm for 10 min after sampling to separate the CM and enzyme solution. The enzyme concentrations in the supernatant were then measured again. The moles of immobilized enzymes (Spy_BacGT_LCI@CM) were determined by the difference in enzyme concentrations before and after incubation. The Spy_BacGT_LCI@CM was washed three times with Tris–HCl buffer (50 mM Tris–HCl, pH 7.5), and stored at –20°C for subsequent use in the Pickering emulsion.

## 1.8. *In vitro* glycosylation reactions

The purified proteins were subjected to *in vitro* glycosylation reactions with 1000 rpm at 37°C for 2 h. **In water-phase glycosylation reaction**: A 200 μL mixture containing 50 mM Tris-HCl buffer (with 10 mM MgCl_2_), different concentrations (2, 4, 6, 8 mM) UDP-Glc (or UDP-Gal), 2 mM substrate (dissolved in DMSO) and 20 μM proteins (BarGT-3, BpsGT, BcyGT, BacGT, BceGT and BarGT-1). **In the biphasic glycosylation reaction system**: A 200 μL mixture containing 50 mM Tris-HCl buffer (with 10 mM MgCl_2_), 4 mM UDP-Glc (or UDP-Gal), 2 mM substrate (dissolved in DMSO), 2-hexanone with different concentrations (10%-60%, vol%) and 20 μM Spy_BacGT (Spy_BarGT-3 or Spy_BarGT-1). The carriers of mesoporous (CM) were added according to weight percent (1%-5%, wt%). **Pickering emulsion system with immobilized protein**: 200 μL mixture containing 50 mM Tris-HCl buffer (with 10 mM MgCl_2_), 4 mM UDP-Glc (or UDP-Gal), 2 mM substrate (dissolved in DMSO), 2-hexanone with different concentrations (10%-60%, vol%) and ~2.1 mg immobilized Spy_BacGT_LCI (Spy_BarGT-3_LCI or Spy_BarGT-1_LCI, the molar equivalent to free Spy_BacGT, Spy_BarGT-3 and Spy_BarGT-1, respectively). A stable Pickering emulsion was obtained by vigorously shearing at 20,000 rpm for 3 min. All the above reactions were terminated with an equal volume of acetonitrile. The post-reaction biphasic system was first centrifuged at 14,000 rpm for 10 min to separate the oil phase (2-hexanone) and the water phase, and the oil phase was dried by nitrogen blowing. Then, the remaining reaction mixtures were terminated with an equal volume of acetonitrile. After centrifugation at 14,000 rpm for 30 min, the reaction mixtures were analyzed by HPLC-MS. To calculate the number of cycles for the immobilized enzymes (Spy_BacGT_LCI@CM or Spy_BarGT-3_LCI@CM), after the first Pickering emulsion catalysis (2 h), the samples were centrifuged at 14,000 rpm for 10 min. The immobilized enzymes were separated from the reaction mixtures, and washed three times with Tris–HCl buffer (50 mM Tris–HCl, pH 7.5). The immobilized enzymes then subjected to the next Pickering emulsion reaction after re-emulsification.

## 1.9. HPLC-PDA, MS and NMR assays

The samples were subjected to HPLC-PDA (HPLC, Nexera X2, Shimadzu Deutschland GmbH, Duisburg, Germany) equipped with a C_18_ column (YMC CO., LTD, C18, 250 mm×4.6 mm, 5 µm), and equipped with an ESI-MS single quadrupole mass detector (ThermoFisher Scientific Surveyor MSQ Plus, Illinois, USA). Masses were determined via the mass/charge ratio (m/z) in a positive mode with 55 V cone voltage and 3 KV needle voltage at 400°C. Products were detected by UV absorbance from 200 to 400 nm. The binary mobile phase consisted of solvent A (HPLC grade purity water) and solvent B (HPLC grade purity acetonitrile) at a flow rate of 1 mL/min. HPLC method used for analysis of glycosylated products was as follows: 10–90% solvent B (0–25 min), 90% solvent B (25.1–29 min), 10% solvent B (29.1–35 min). For the preparative scale reaction, a 20 mL system containing immobilized enzymes (~10 mg), 10 mM UDP-Glc (or UDP-Gal), 5 mM substrate (dissolved in DMSO), 30% 2-hexanone (vol%), 50 mM Tris-HCl buffer (with 10 mM MgCl_2_). The Pickering emulsion was obtained by vigorously shearing at 20,000 rpm for 3 min (emulsification), and incubated with 1000 rpm for 2 h at 37°C. After centrifugation at 14,000 rpm for 10 min, the oil phase (2-hexanone) and the water phase were stopped by adding the same volume of acetonitrile, respectively. Centrifugation continued at 14,000 rpm and 4°C for 30 min, and the supernatant was concentrated by rotary evaporator, and separated by reversed-phase semipreparative HPLC (Shimadzu Deutschland GmbH, Duisburg, Germany) using a C_18_ column (CS-Chromatographie Service GmbH, Dueren, Germany, 125 mm × 8 mm, 10 μm) at a flow rate of 1 mL/min. 1D and 2D NMR spectra of each product (~ 10 mg) were recorded on a Bruker Avance III 400 (or 600) NMR spectrometer (Bruker BioSpin, Germany).

## 1.10. Mass transfer assays for substrate/product

Before adding Spy_BacGT_LCI@CM, the substrate (acacetin) was dissolved in the biphasic system and vigorously sheared at 20,000 rpm for 3 min. Subsequently, the concentrations of the substrate and product aca-Glc (tilianin) were measured in both the oil phase (2-hexanone) and the water phase (according to the standard curves for the substrate and product). Then, Spy_BacGT_LCI@CM was added, and the reaction solutions were vigorously sheared again at 20,000 rpm for 3 min to form a Pickering emulsion. Samples were taken at 20, 40, 60, 80, 100, and 120 min, with three replicates for each time point. The samples were collected and centrifuged at 14,000 rpm for 10 min to separate the CM and solutions. The concentrations of the substrate and product in both the oil phase and the water phase were measured according to the standard curves for the substrate and product.

## 1.11. Characterization of circular dichroism

Circular dichroism measurements were performed on Chirascan V100 (Applied Photophysics Ltd, UK). After 8^th^ reaction, the mixture was washed with buffer. Measurements of the CD spectra were made by taking 200 μL of washed buffer. The CD spectra were recorded at room temperature in the range of 190–260 nm and the path length in the cuvette was 1.0 mm.

# 2. Additional Figures and Tables


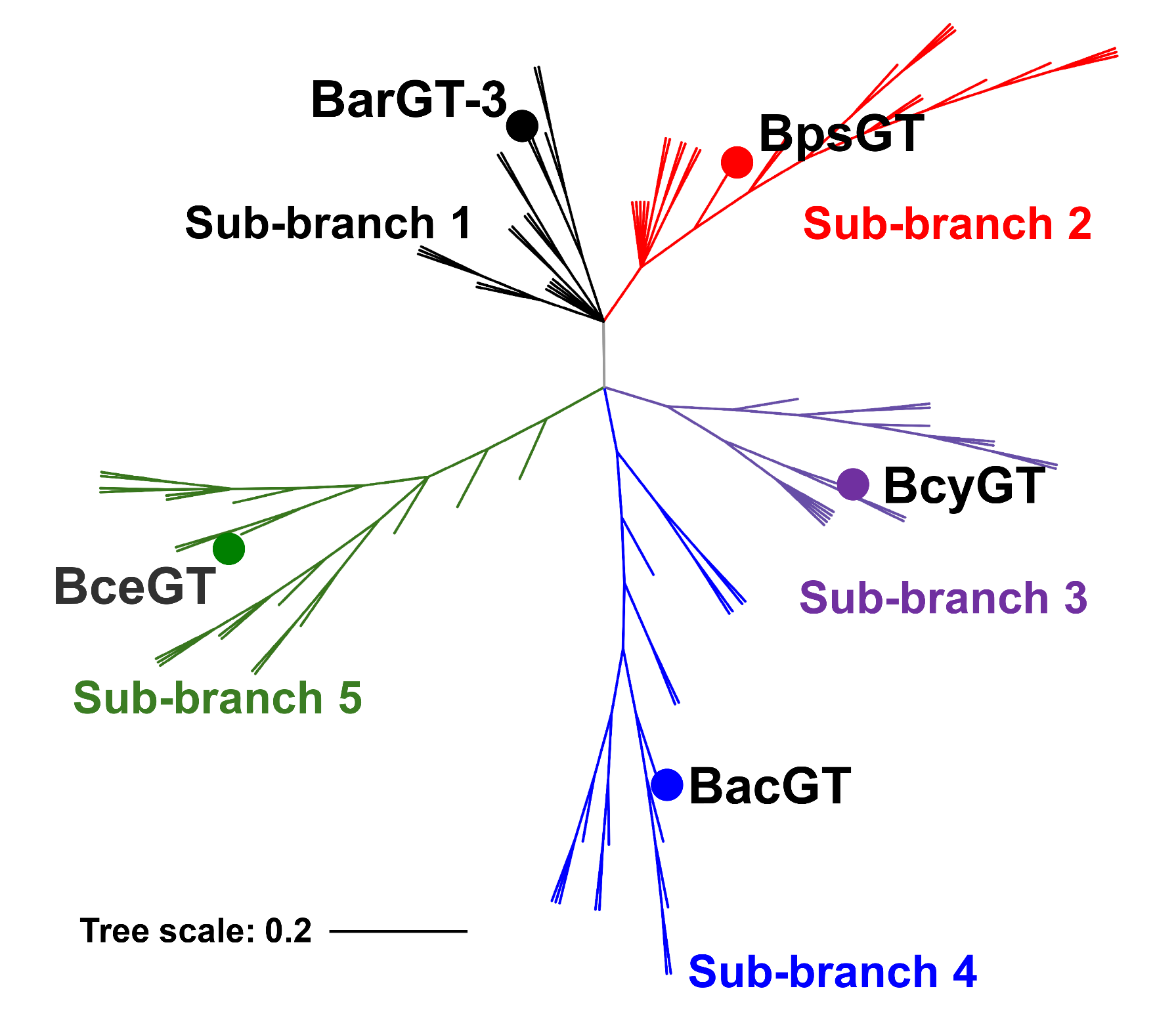


## Fig. S1. Phylogenetic analysis of GTs in the nBGT3 branch and selection of representative enzymes in sub-branches through CD-HIT clustering.


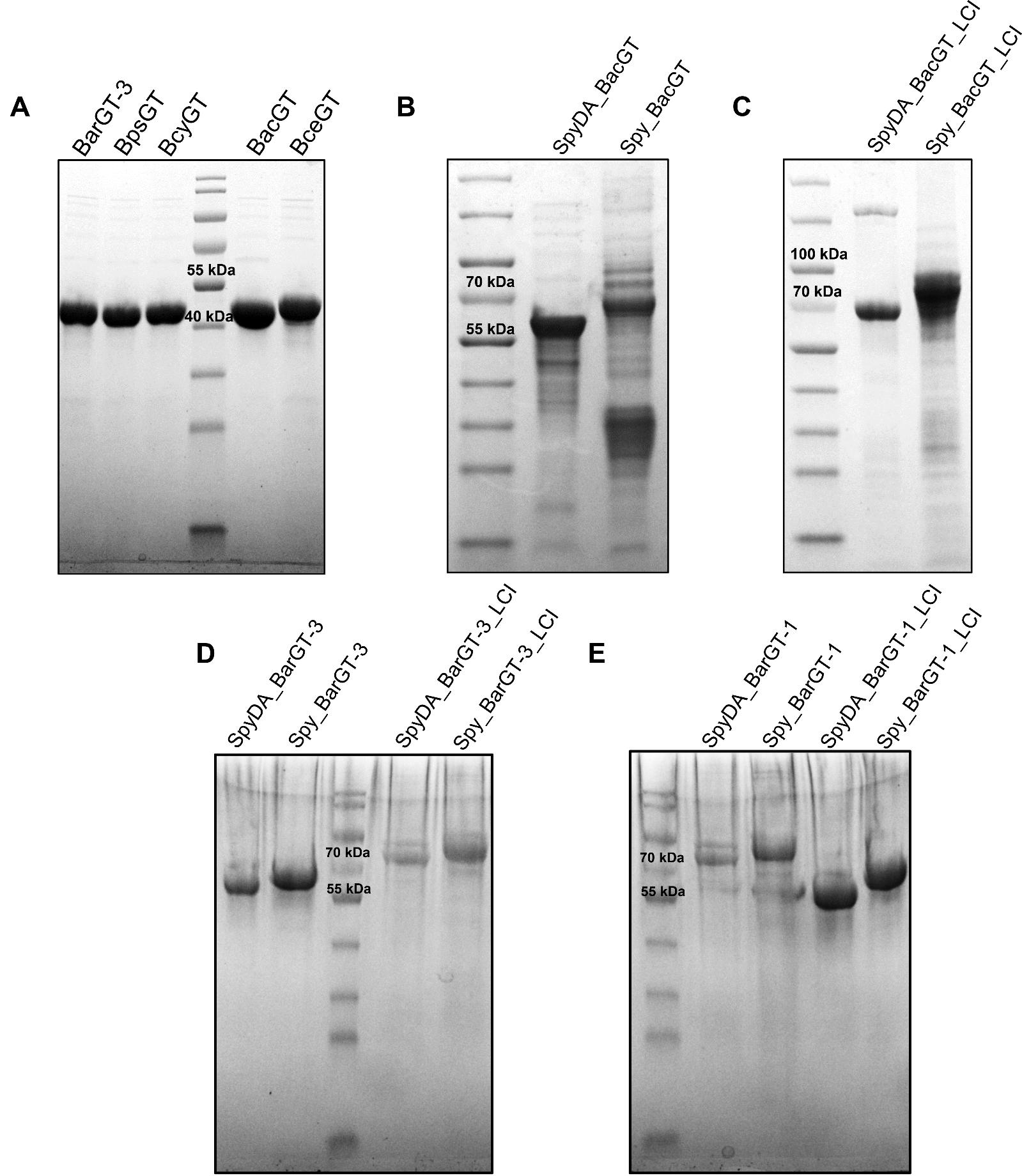


## Fig. S2. The SDS-PAGE detection of the purification of GT proteins. (A). The purification of GTs BarGT-3, BpsGT, BcyGT, BacGT and BceGT. (B). The purification of cyclized protein (Spy_BacGT) and non-cyclized BacGT (SpyDA_BacGT). (C). The purification of cyclized protein with anchor peptide LCI (Spy_BacGT_LCI) and non-cyclized BacGT (SpyDA_BacGT_LCI). (D). The purification of cyclized protein (Spy_BarGT-3) and non-cyclized BarGT-3 (SpyDA_BarGT-3) and the purification of cyclized protein with anchor peptide LCI (Spy_BarGT-3_LCI) and non-cyclized BarGT-3 (SpyDA_BarGT-3_LCI). (E). The purification of cyclized protein (Spy_BarGT-1) and non-cyclized BarGT-1 (SpyDA_BarGT-1) and the purification of cyclized protein with anchor peptide LCI (Spy_BarGT-1_LCI) and non-cyclized BarGT-1 (SpyDA_BarGT-1_LCI).


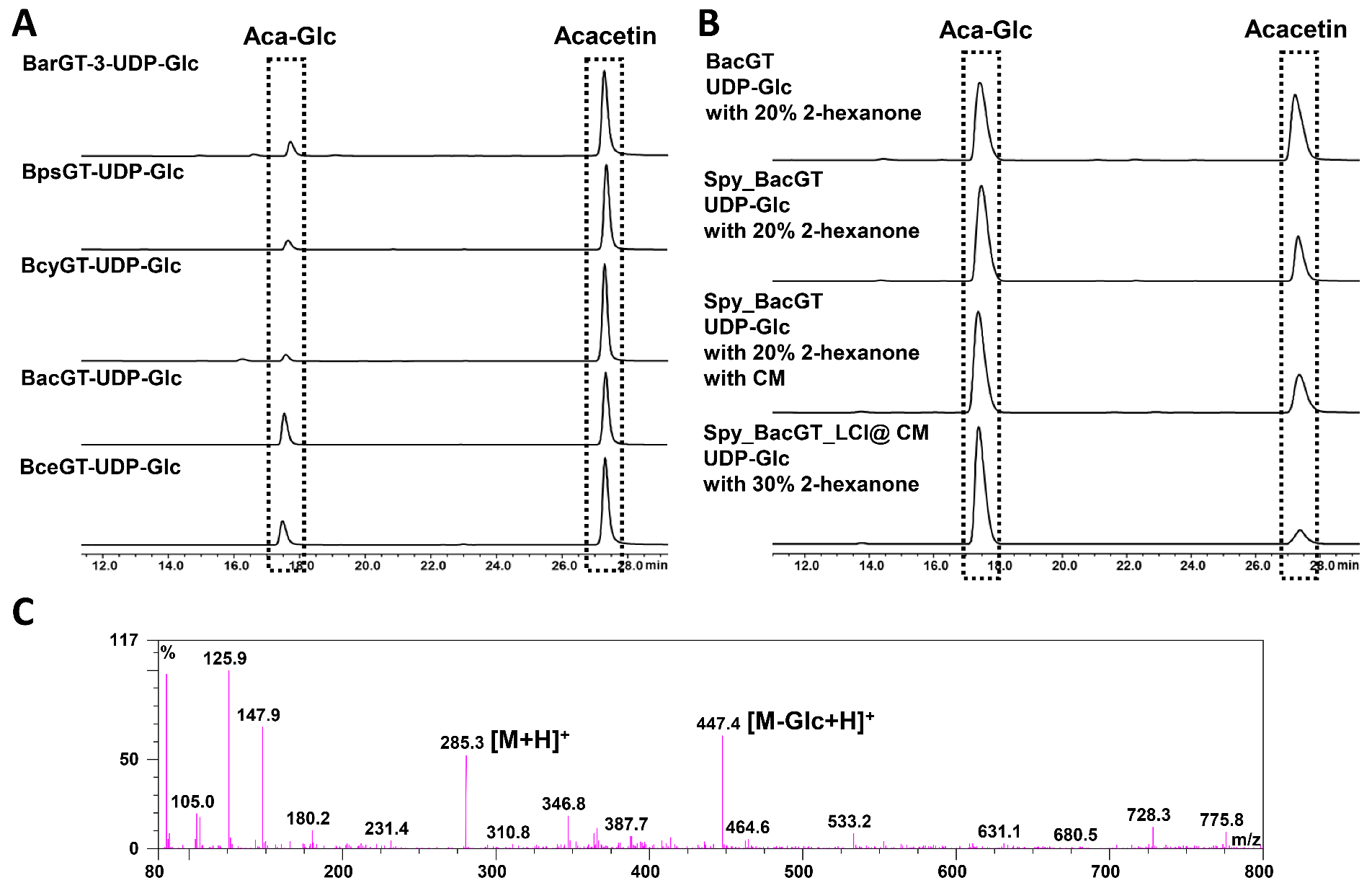


## Fig. S3. HPLC and positive ion mass spectrometry (MS) analysis for product aca-Glc. (A). The activities of five GTs (BarGT-3, BpsGT, BcyGT, BacGT and BceGT) towards substrate acacetin were determined by HPLC. (B). The activities of BacGT, Spy_BacGT, and Spy_BacGT_LCI with oil phase (2-hexanone) and carriers of mesoporous (CM) towards substrate acacetin were determined by HPLC. (C). The product aca-Glc ([M-Glc+H]^+^) was identified by positive ion MS.


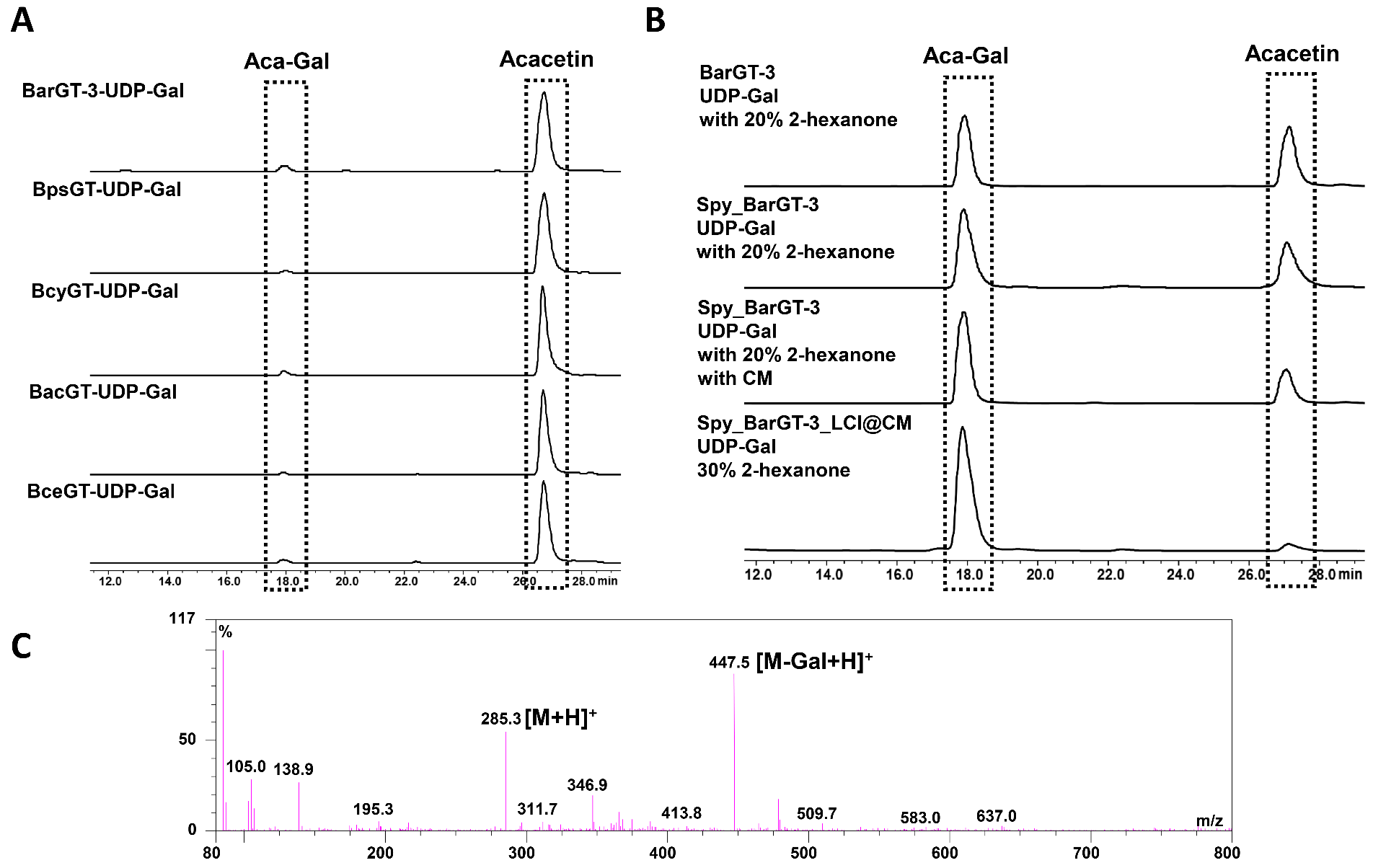


## Fig. S4. HPLC and positive ion mass spectrometry (MS) analysis for product aca-Gal. (A). The activities of five GTs (BarGT-3, BpsGT, BcyGT, BacGT and BceGT) towards substrate acacetin were determined by HPLC. (B). The activities of BacGT, Spy_BacGT, and Spy_BacGT_LCI with oil phase (2-hexanone) and carriers of mesoporous (CM) towards substrate acacetin were determined by HPLC. (C). The product aca-Gal ([M-Gal+H]^+^) was identified by positive ion MS.


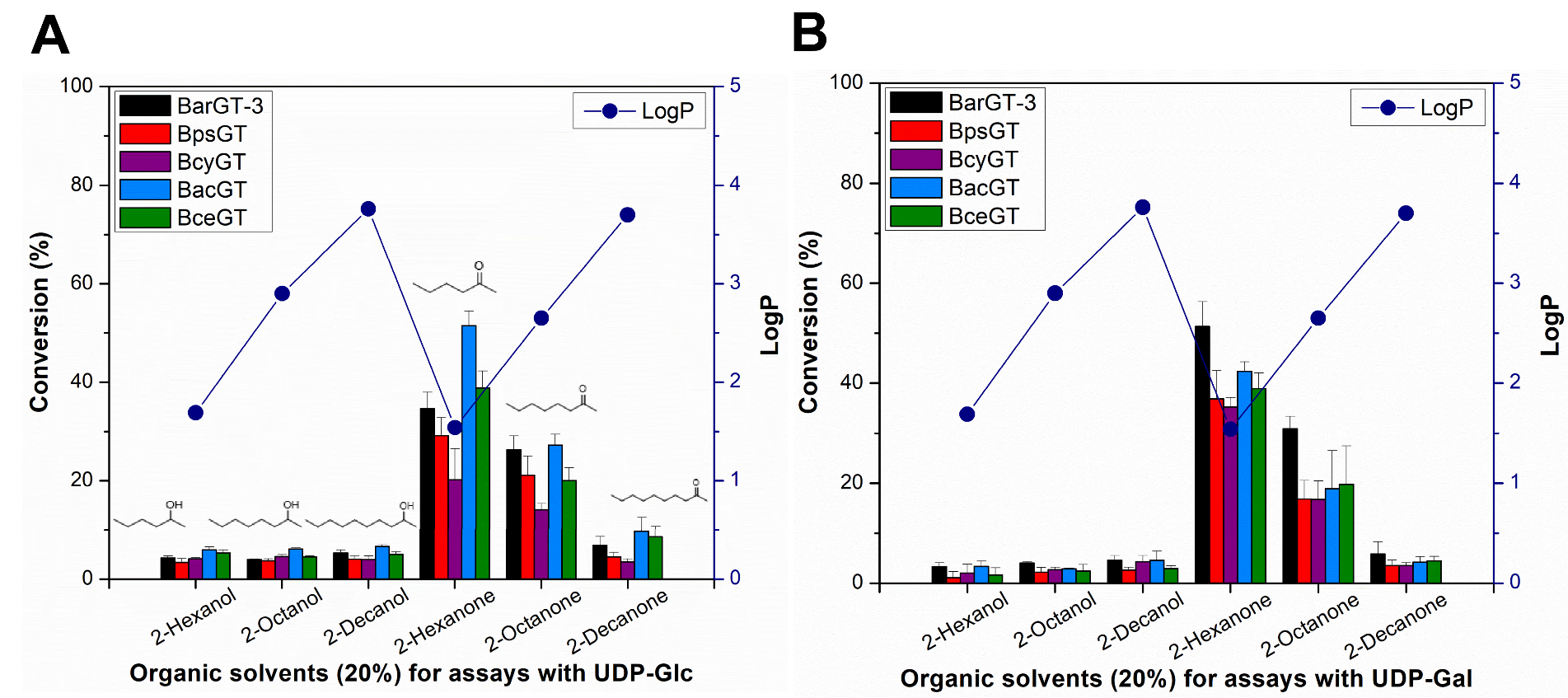


## Fig. S5. 20% (volume concentration, vol%) long-chain carbon alcohols and ketones (6 to 10 carbons) were selected for the biphasic glycosylation assays using 5 GTs with UDP-Glc (A) and UDP-Gal (B) and logP values were labeled (right column).


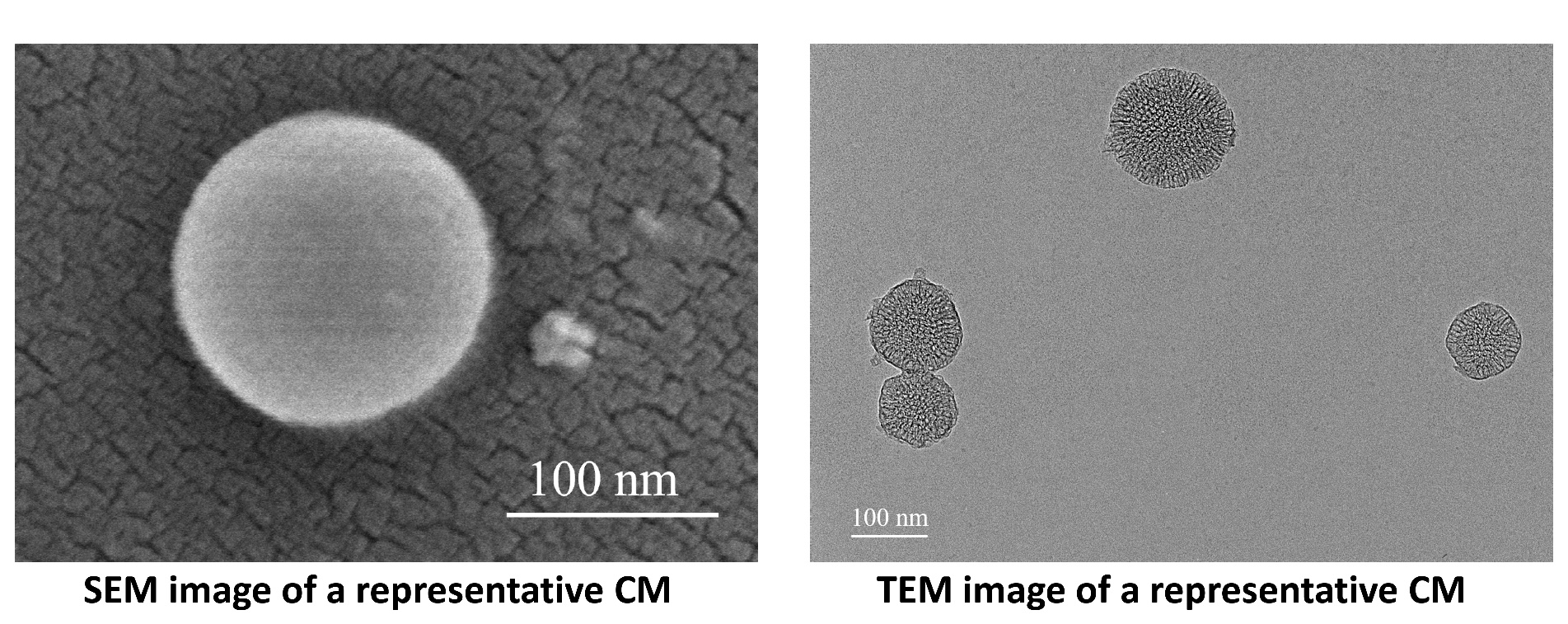


## Fig. S6. The images of scanning electron microscope (SEM) and transmission electron microscope (TEM) of representative carriers of mesoporous (CM).


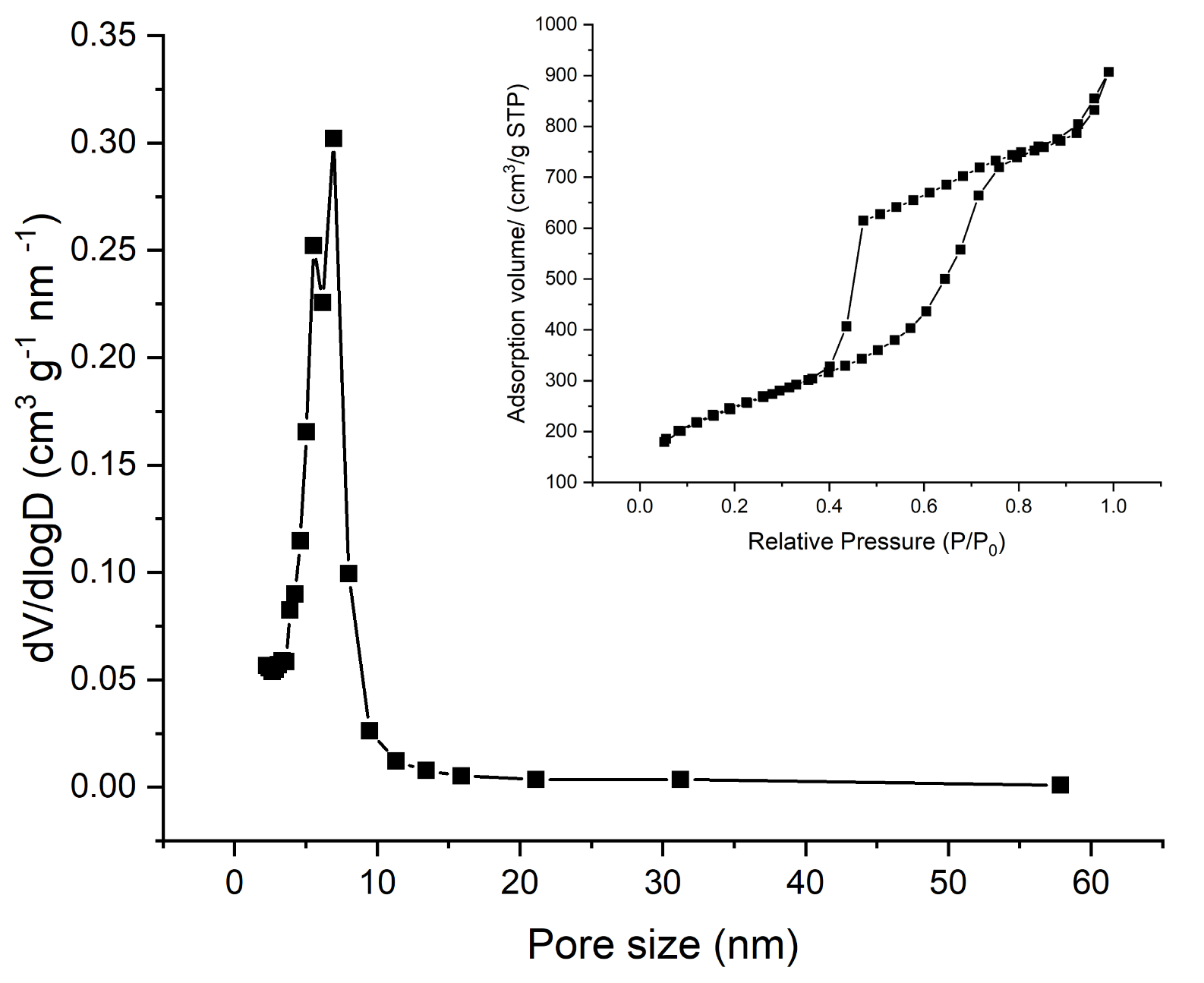


## Fig. S7. Nitrogen adsorption/desorption isotherms and pore diameter distributions of carriers of mesoporous (CM).


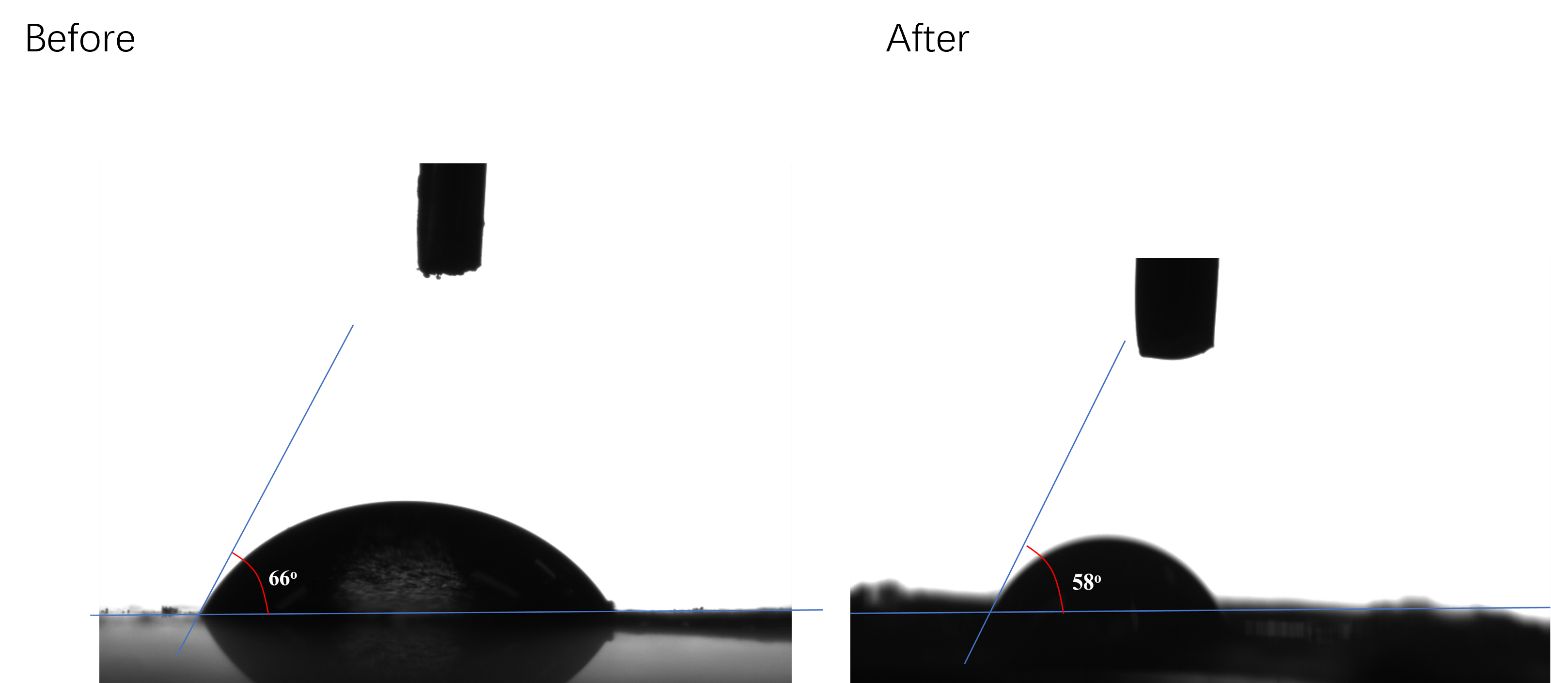


## Fig. S8. The air-water contact angle for CM before and after enzyme immobilization.


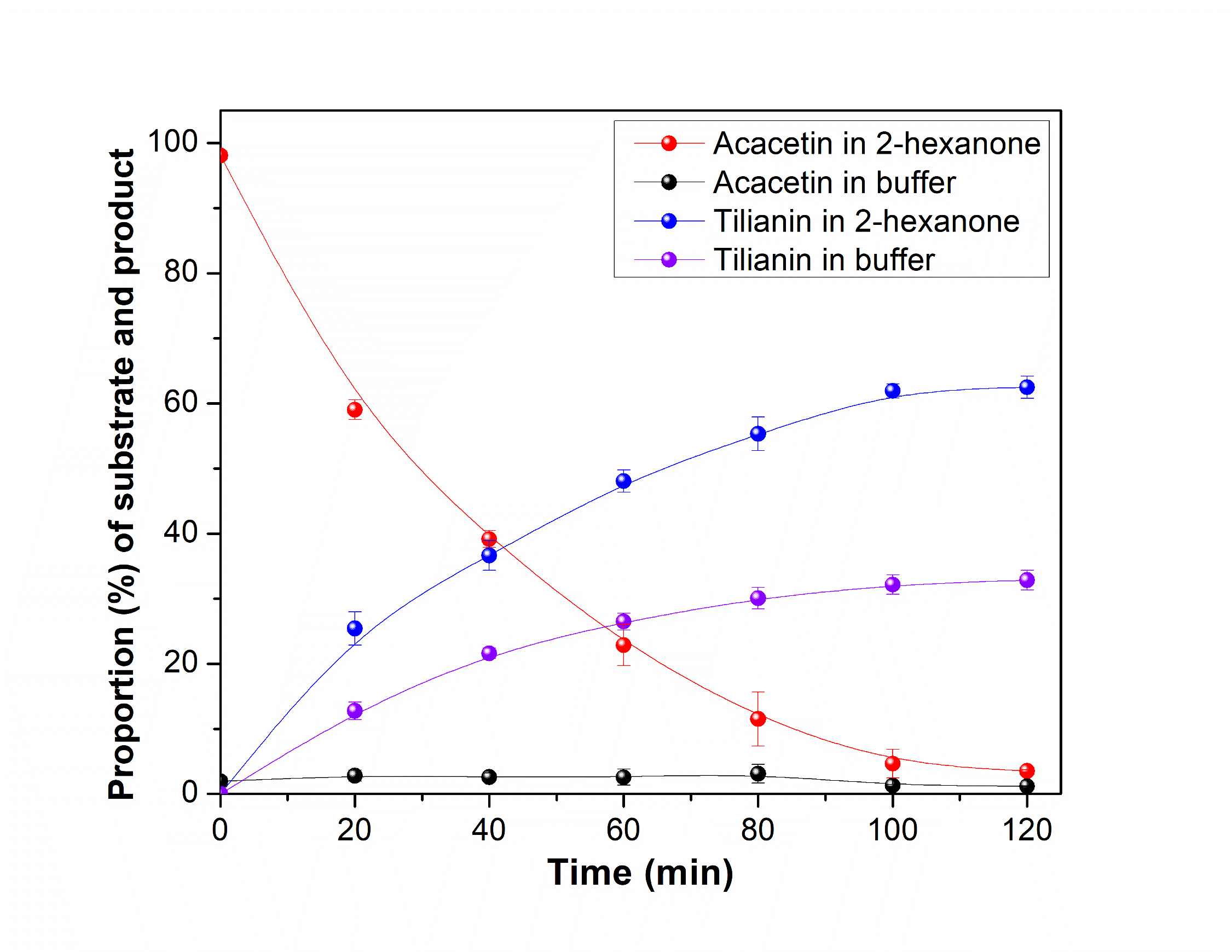


## Fig. S9. The proportions of the substrate (acacetin) and product (aca-Glc) in the oil phase (2-hexanone) and water phase over 2 h.


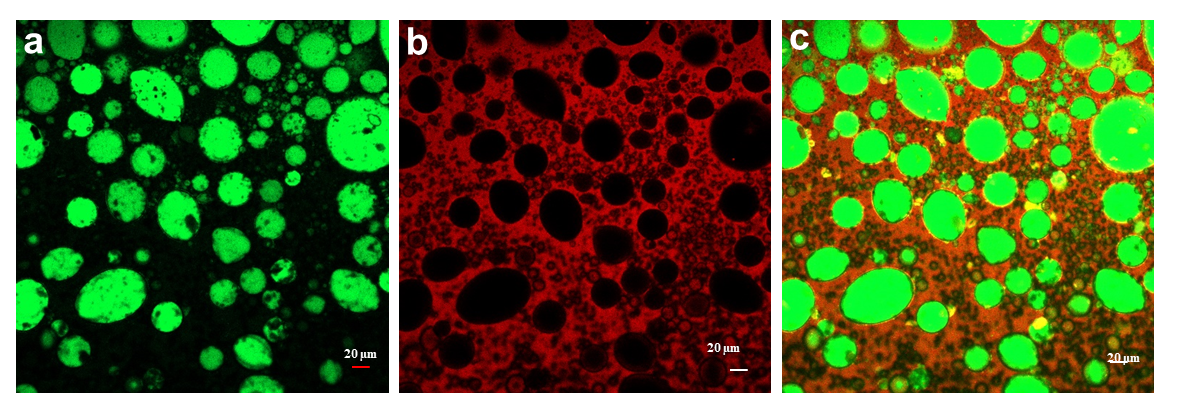


## Fig. S10. The Confocal laser scanning microscopy (CLSM) images of the droplets (Pickering emulsion system, Spy_BacGT_LCI@CM with UDP-Glc in the 8^th^ reaction cycle) dyed with (a) Fluorescein isothiocyanate (FITC), water-soluble, and (b) Nile red, oil-soluble/organic phase (2-hexanone), and (c) an individual water-in-oil Pickering emulsion.





## Fig. S11. Circular dichroism spectra of the washed buffer from the 8^th^ reaction cycle. These spectroscopic measurements were carried out to examine the existence of protein and its secondary structure of enzyme in the 190-260 nm range.

## Table S1. Sequence identity among the five GT candidates (BarGT-3, BpsGT, BcyGT, BacGT, BceGT).

| **GTs (%)** | **BarGT-3** | **BpsGT** | **BcyGT** | **BacGT** |
| --- | --- | --- | --- | --- |
| **BpsGT** | 75.3 |  |  |  |
| **BcyGT** | 66.5 | 70.9 |  |  |
| **BacGT** | 51.9 | 51 | 50.1 |  |
| **BceGT** | 54.9 | 56 | 57.2 | 71 |

## 3. ^1^H and ^13^C NMR spectral data of glycosylated products (Fig. S12-S36)

**(1). Aca-Glc and aca-Gal**

**Aca-Glc: acacetin 7-O-β-D glucoside**

**^1^H NMR (400 MHz, DMSO-*d*_6_)** δ 12.87 (s, 1H), 8.02 (d, *J* = 8.9 Hz, 2H), 7.08 (d, *J* = 9.0 Hz, 2H), 6.91 (s, 1H), 6.81 (d, *J* = 2.1 Hz, 1H), 6.41 (d, *J* = 2.1 Hz, 1H), 5.37 (d, *J* = 4.9 Hz, 1H), 5.10 (d, *J* = 4.7 Hz, 1H), 5.06 – 4.89 (m, 2H), 4.57 (t, *J* = 5.6 Hz, 1H), 3.81 (s, 3H), 3.66 (dd, *J* = 10.1, 5.3 Hz, 1H), 3.49 – 3.35 (m, 2H), 3.27 – 3.02 (m, 3H). **^13^C NMR (101 MHz, DMSO)** δ 182.1, 163.8, 163.0, 162.5, 161.1, 157.0, 128.5, 128.3, 122.7, 114.6, 114.5, 105.4, 103.8, 99.9, 99.6, 94.9, 77.2, 76.4, 73.1, 69.5, 60.6, 55.6.

**Aca-Gal: acacetin 7-*O*-β-D galactoside**

**^1^H NMR (400 MHz, DMSO-*d*_6_)** δ 12.87 (s, 1H), 8.09 – 7.95 (m, 2H), 7.15 – 7.03 (m, 2H), 6.92 (s, 1H), 6.81 (d, *J* = 2.2 Hz, 1H), 6.41 (d, *J* = 2.2 Hz, 1H), 5.36 (d, *J* = 4.9 Hz, 1H), 5.10 (d, *J* = 4.8 Hz, 1H), 5.06 – 4.96 (m, 2H), 4.63 – 4.54 (m, 1H), 3.82 (s, 3H), 3.66 (dd, *J* = 10.0, 5.3 Hz, 1H), 3.47 – 3.36 (m, 2H), 3.26 – 3.10 (m, 3H). **^13^C NMR (101 MHz, DMSO-*d*_6_)** δ 182.1, 163.8, 163.0, 162.5, 161.1, 157.0, 128.5, 128.5, 122.7, 114.7, 114.6, 105.4, 103.8, 99.9, 99.6, 94.9, 77.2, 76.4, 73.1, 69.5, 60.6, 55.6.


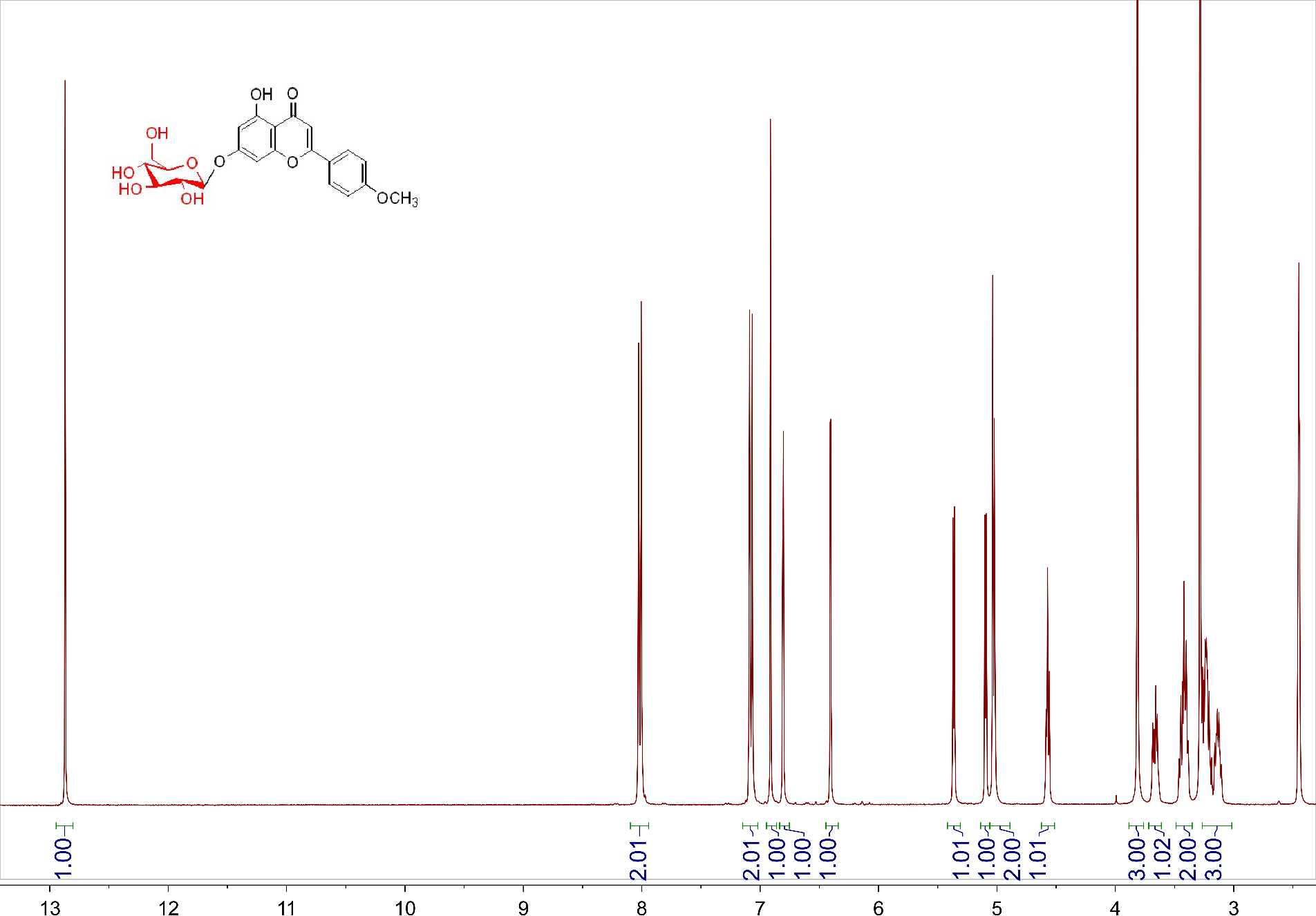


**Fig. S12.** ^1^H NMR spectrum of aca-Glc (400 MHz, DMSO-d_6_)


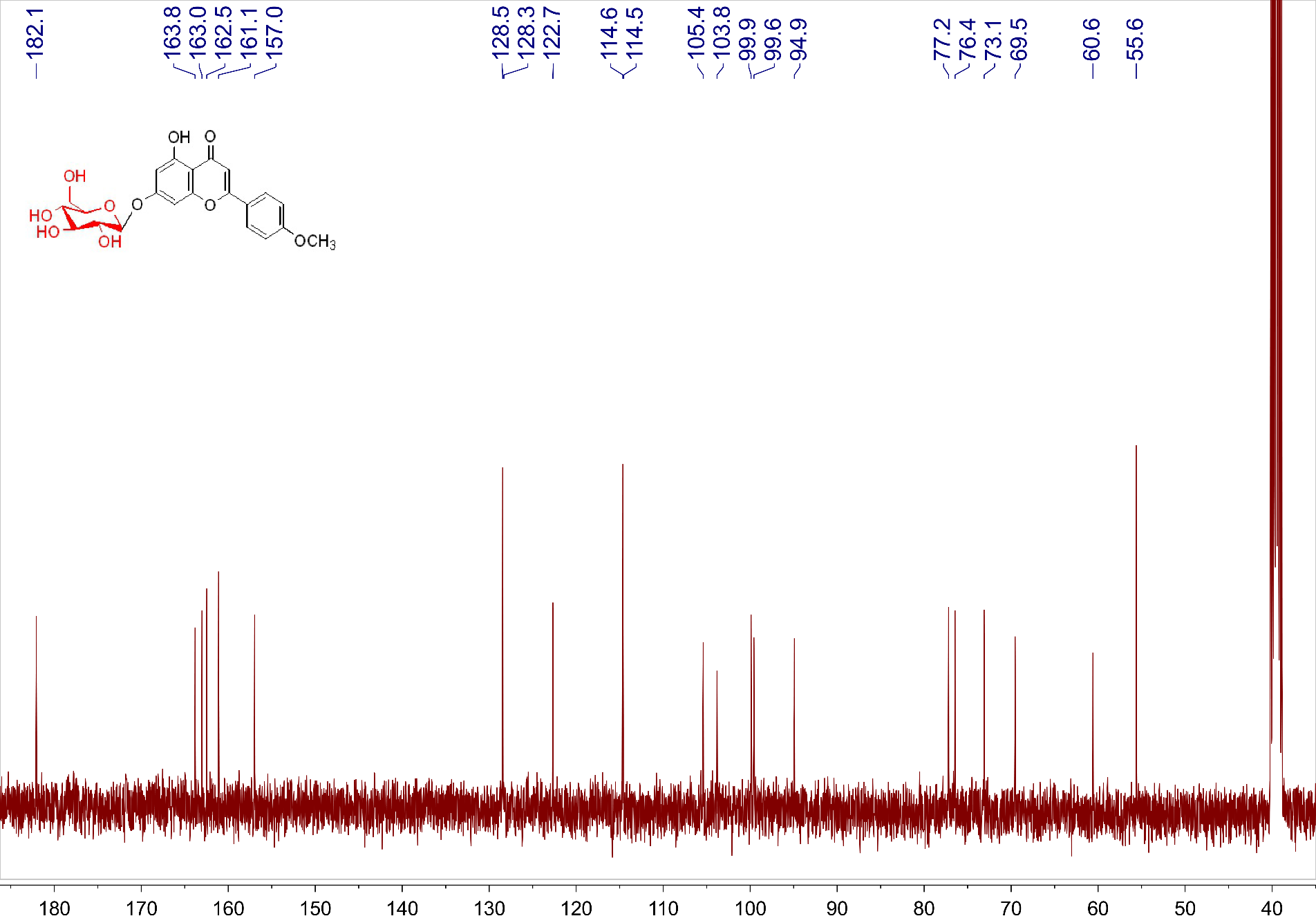


**Fig. S13.** ^13^C NMR spectrum of aca-Glc (101 MHz, DMSO-d_6_)


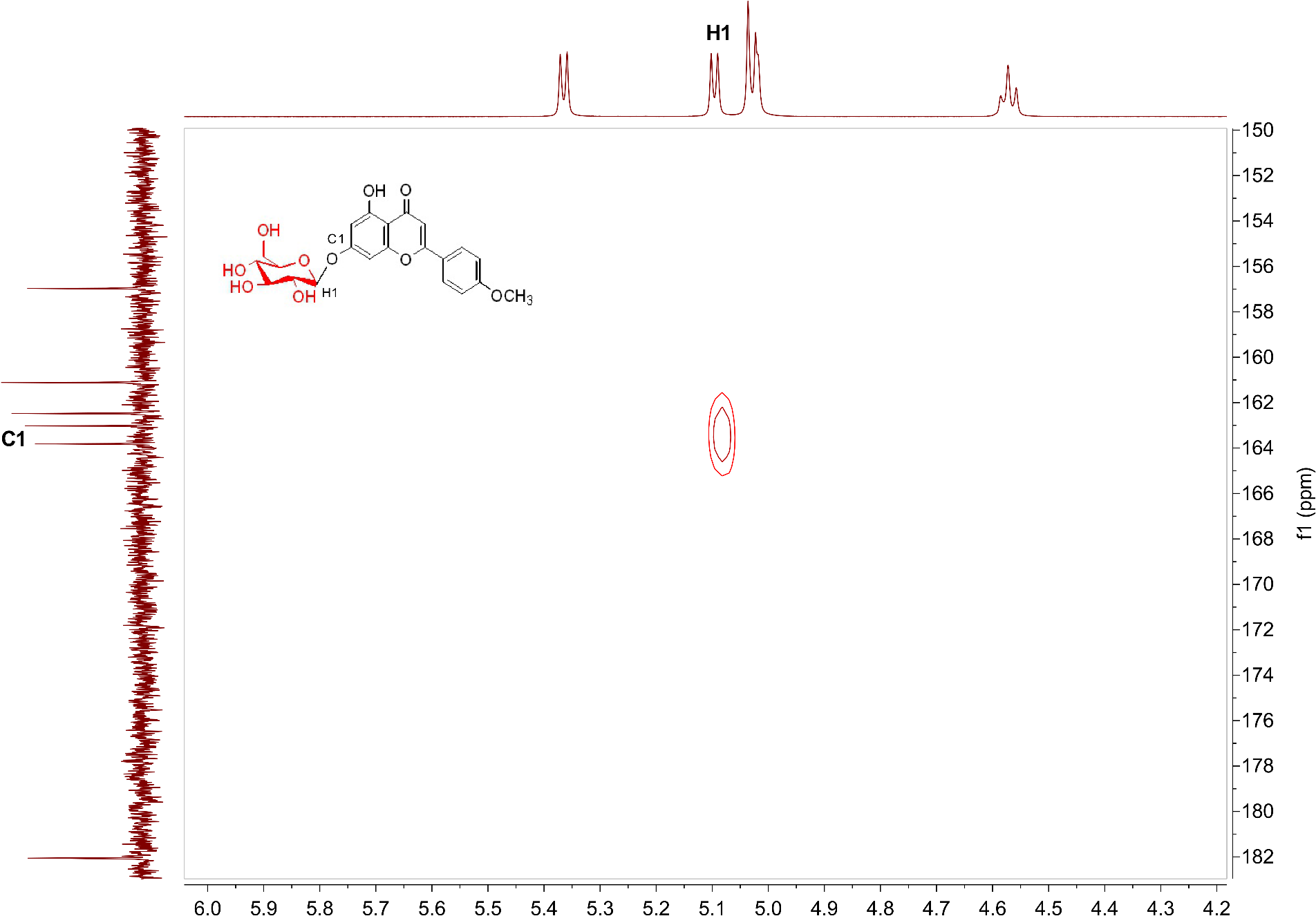


**Fig. S14.** HMBC spectrum of aca-Glc (400 MHz/101 MHz, DMSO-d_6_)


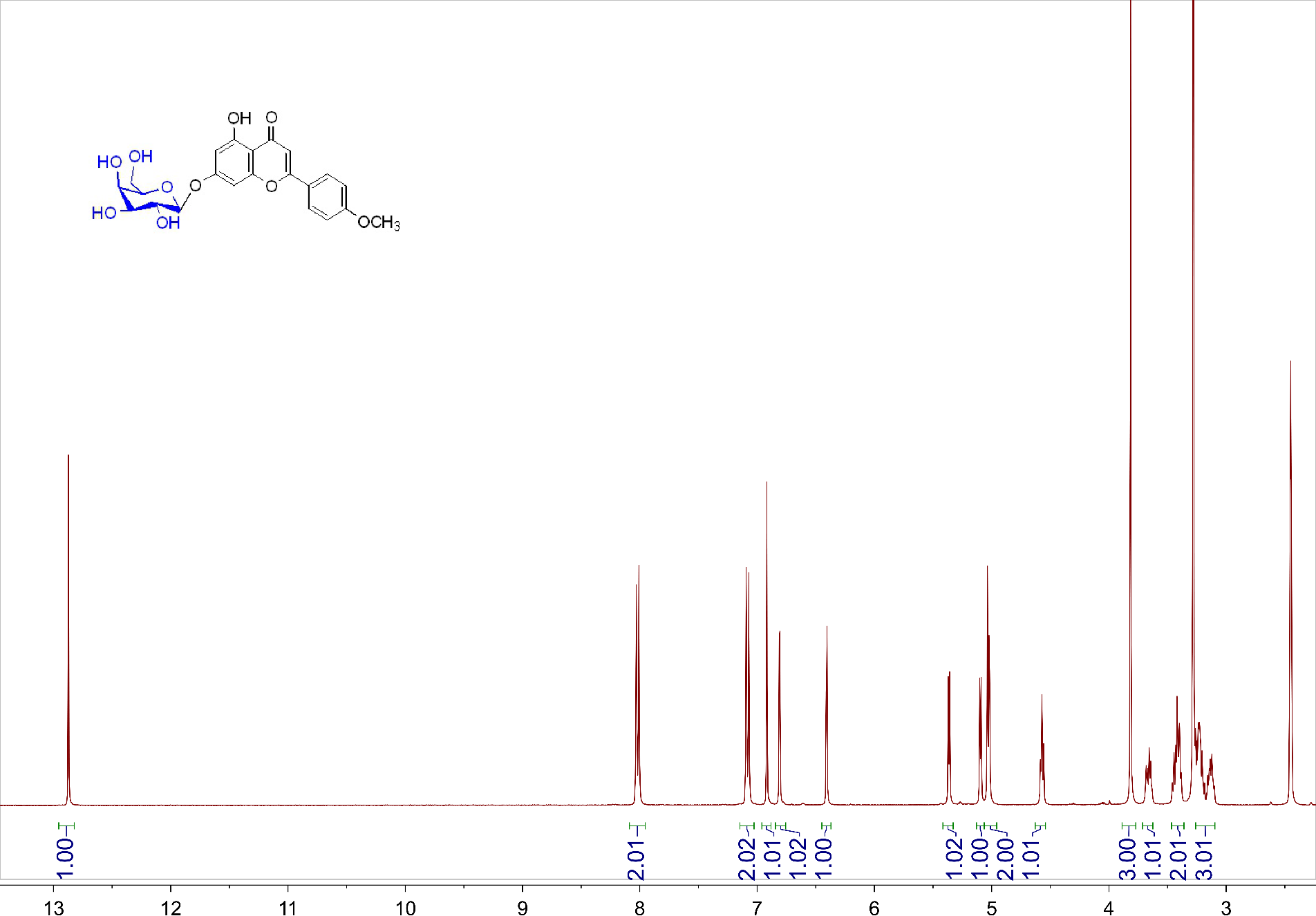


**Fig. S15.** ^1^H NMR spectrum of aca-Gal (400 MHz, DMSO-d_6_)


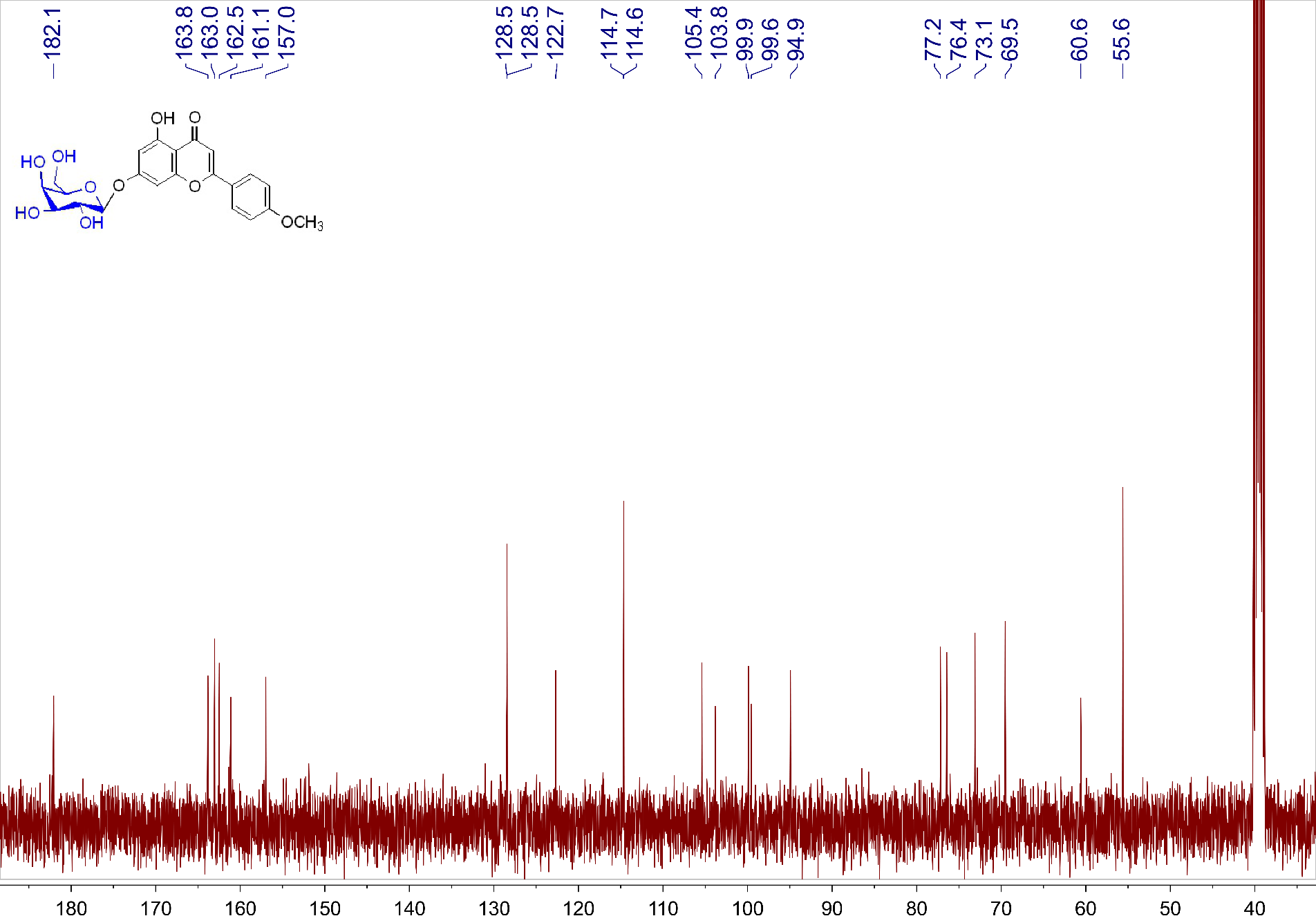


**Fig. S16.** ^13^C NMR spectrum of aca-Gal (101 MHz, DMSO-d_6_)


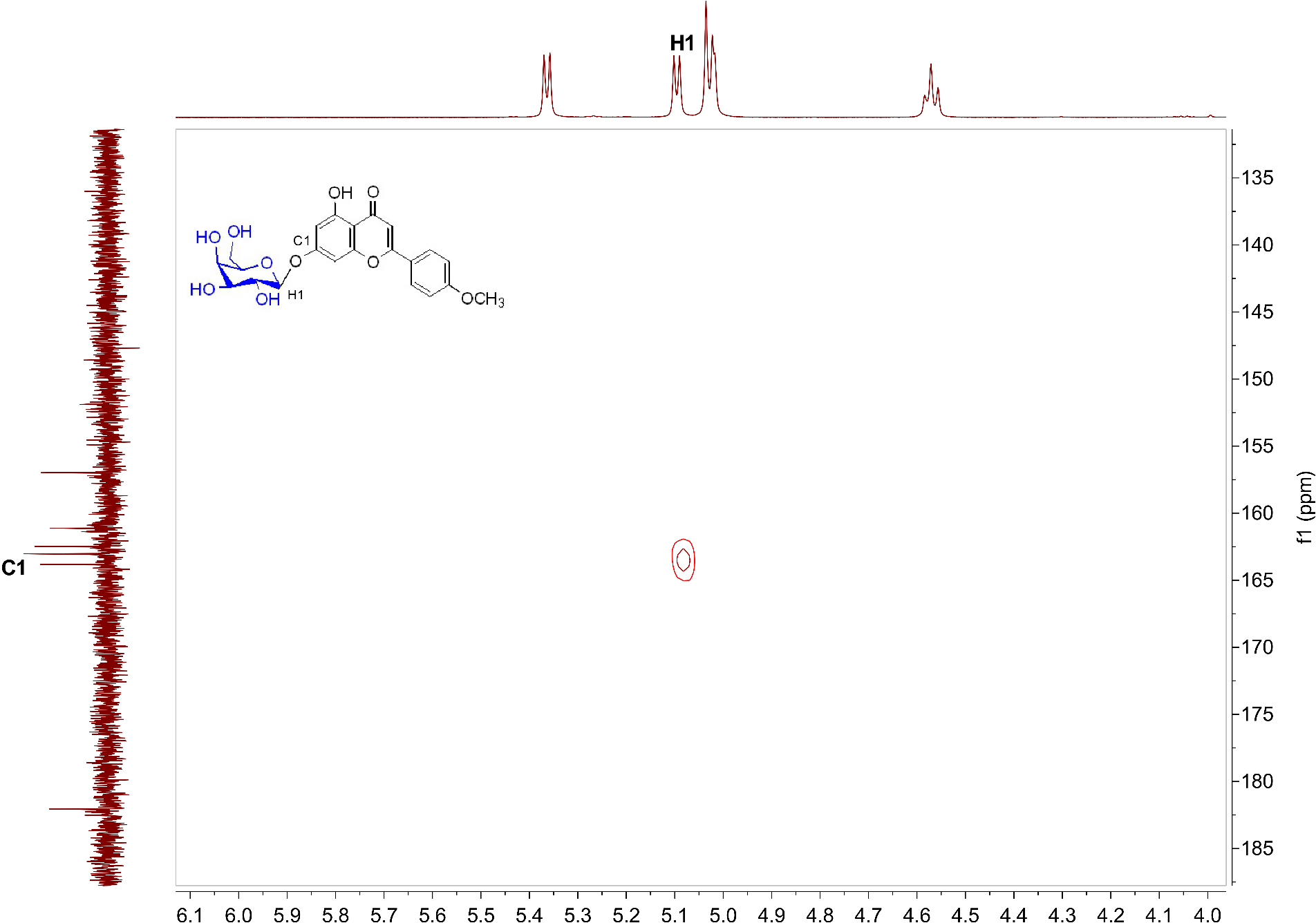


**Fig. S17.** HMBC spectrum of aca-Gal (400 MHz/101 MHz, DMSO-d_6_)

**(2). MS and NMR spectral data for glycosylated products 1a-6a**

**

**

**Fig. S18.** Positive ion mass spectrometry (MS) analysis for glycosylated products **1a**-**6a**

**^1^H and ^13^C NMR spectral data of the glycosylated products 1a-6a**

**1a**: 1-naphthol *O*-β-D-glucoside

**^1^H NMR (400 MHz, DMSO-*d*_6_)** δ 8.33 (dd, *J* = 7.3, 2.4 Hz, 1H), 7.88 (dd, *J* = 7.0, 2.6 Hz, 1H), 7.57 – 7.47 (m, 3H), 7.42 (t, *J* = 7.9 Hz, 1H), 7.19 (d, *J* = 7.7 Hz, 1H), 5.47 (d, *J* = 5.4 Hz, 1H), 5.13 (d, *J* = 4.9 Hz, 1H), 5.05 (d, *J* = 5.3 Hz, 1H), 5.01 (d, *J* = 7.7 Hz, 1H), 4.59 (t, *J* = 5.7 Hz, 1H), 3.73 (ddd, *J* = 11.7, 5.3, 2.1 Hz, 1H), 3.54 – 3.42 (m, 2H), 3.42 – 3.33 (m, 2H), 3.31 (d, *J* = 4.8 Hz, 1H), 3.23 (td, *J* = 9.2, 5.2 Hz, 1H). **^13^C NMR (101 MHz, DMSO)** δ 152.8, 133.8, 127.1, 126.5, 126.2, 125.9, 125.1, 121.9, 121.1, 108.8, 100.9, 77.0, 76.4, 73.2, 69.6, 60.5.

**2a**: 2-naphthol *O*-β-D-glucoside

**^1^H NMR (400 MHz, DMSO-*d*_6_)** δ 7.83 (dd, *J* = 8.7, 3.3 Hz, 2H), 7.77 (d, *J* = 7.9 Hz, 1H), 7.48 – 7.41 (m, 2H), 7.35 (ddd, *J* = 8.2, 6.8, 1.3 Hz, 1H), 7.25 (dd, *J* = 8.9, 2.6 Hz, 1H), 5.36 (d, *J* = 4.9 Hz, 1H), 5.11 (d, *J* = 4.6 Hz, 1H), 5.03 (dd, *J* = 12.2, 6.4 Hz, 2H), 4.59 (t, *J* = 5.7 Hz, 1H), 3.72 (ddd, *J* = 11.7, 5.3, 2.1 Hz, 1H), 3.49 (dt, *J* = 11.9, 6.1 Hz, 1H), 3.40 (ddd, *J* = 9.8, 5.7, 2.1 Hz, 1H), 3.29 (td, *J* = 4.8, 2.6 Hz, 2H), 3.19 (td, *J* = 9.1, 5.3 Hz, 1H). **^13^C NMR (101 MHz, DMSO)** δ 155.4, 134.2, 129.4, 129.2, 127.7, 127.1, 126.5, 124.2, 119.0, 110.6, 100.7, 77.3, 76.9, 73.5, 69.9, 60.9.

**3a**: 3-hydroxyflavone *O*-β-D-glucoside

**^1^H NMR (400 MHz, DMSO-*d*_6_)** δ 8.16 (dq, *J* = 5.4, 2.3 Hz, 2H), 8.11 (dd, *J* = 8.0, 1.6 Hz, 1H), 7.84 (ddd, *J* = 8.7, 7.0, 1.7 Hz, 1H), 7.78 – 7.72 (m, 1H), 7.56 – 7.49 (m, 4H), 5.58 (d, *J* = 7.8 Hz, 1H), 5.38 (d, *J* = 4.7 Hz, 1H), 5.08 (d, *J* = 4.7 Hz, 1H), 4.97 (s, 1H), 4.29 (d, *J* = 26.5 Hz, 3H), 3.54 (dd, *J* = 11.6, 4.7 Hz, 1H), 3.16 – 3.12 (m, 1H), 3.07 (dd, *J* = 4.5, 2.6 Hz, 2H). **^13^C NMR (101 MHz, DMSO)** δ 173.5, 155.6, 154.7, 136.2, 134.1, 130.6, 130.6, 129.0, 128.1, 125.1, 124.9, 123.3, 118.3, 100.4, 77.4, 76.4, 74.0, 69.8, 63.4, 60.7, 56.6.

**4a**: 4’-hydroxyflavone *O*-β-D-glucoside

**^1^H NMR (400 MHz, DMSO-*d*_6_)** δ 8.06 (d, *J* = 8.8 Hz, 2H), 8.02 (d, *J* = 7.9 Hz, 1H), 7.81 (td, *J* = 7.7, 7.1, 1.3 Hz, 1H), 7.77 (d, *J* = 8.2 Hz, 1H), 7.48 (t, *J* = 7.4 Hz, 1H), 7.19 (d, *J* = 8.9 Hz, 2H), 6.96 (s, 1H), 5.37 (d, *J* = 4.6 Hz, 1H), 5.11 (d, *J* = 3.9 Hz, 1H), 5.05 (d, *J* = 5.0 Hz, 1H), 5.01 (d, *J* = 7.3 Hz, 1H), 4.58 (t, *J* = 5.6 Hz, 1H), 3.68 (dd, *J* = 11.9, 3.5 Hz, 1H), 3.45 (dd, *J* = 11.8, 5.9 Hz, 1H), 3.42 – 3.35 (m, 2H), 3.25 – 3.13 (m, 2H). **^13^C NMR (101 MHz, DMSO)** δ 175.9, 161.4, 159.0, 154.5, 133.1, 127.0, 124.3, 123.7, 123.3, 122.2, 117.4, 115.5, 104.6, 98.8, 76.1, 75.5, 72.1, 68.6, 60.5, 59.5.

**5a**: 6-hydroxyflavone *O*-β-D-glucoside

**^1^H NMR (400 MHz, DMSO-*d*_6_)** δ 8.10 (dd, *J* = 7.7, 2.0 Hz, 2H), 7.77 (d, *J* = 9.0 Hz, 1H), 7.64 – 7.54 (m, 4H), 7.51 (dd, *J* = 9.2, 3.1 Hz, 1H), 7.01 (s, 1H), 5.37 (d, *J* = 4.9 Hz, 1H), 5.10 (d, *J* = 4.8 Hz, 1H), 5.02 (d, *J* = 5.3 Hz, 1H), 4.96 (d, *J* = 7.3 Hz, 1H), 4.57 (t, *J* = 5.8 Hz, 1H), 3.68 (ddd, *J* = 11.6, 5.3, 2.1 Hz, 1H), 3.49 (p, *J* = 5.9 Hz, 1H), 3.35 (td, *J* = 5.5, 5.0, 2.2 Hz, 1H), 3.28 – 3.06 (m, 3H). **^13^C NMR (101 MHz, DMSO)** δ 177.0, 162.6, 154.9, 151.2, 132.0, 131.4, 129.3, 126.8, 126.6, 124.3, 124.3, 120.2, 109.7, 107.9, 106.4, 101.3, 77.3, 76.6, 73.5, 69.8, 60.8.

**6a**: 7-hydroxyflavone *O*-β-D-glucoside

**^1^H NMR (600 MHz, DMSO-*d*_6_)** δ 8.08 (dt, *J* = 6.4, 1.7 Hz, 2H), 7.96 (d, *J* = 8.9 Hz, 1H), 7.63 – 7.55 (m, 3H), 7.40 (d, *J* = 2.4 Hz, 1H), 7.14 (dd, *J* = 8.9, 2.4 Hz, 1H), 6.97 (s, 1H), 5.12 (d, *J* = 7.4 Hz, 1H), 3.73 – 3.68 (m, 2H), 3.50 – 3.44 (m, 3H), 3.33 – 3.27 (m, 3H), 3.19 (t, *J* = 9.0 Hz, 2H). **^13^C NMR (151 MHz, DMSO)** δ 176.4, 162.3, 161.7, 157.1, 131.7, 131.2, 129.1, 126.2, 126.2, 118.0, 115.5, 106.8, 103.8, 100.0, 77.2, 76.5, 73.1, 69.5, 60.8, 60.5, 40.4.

**
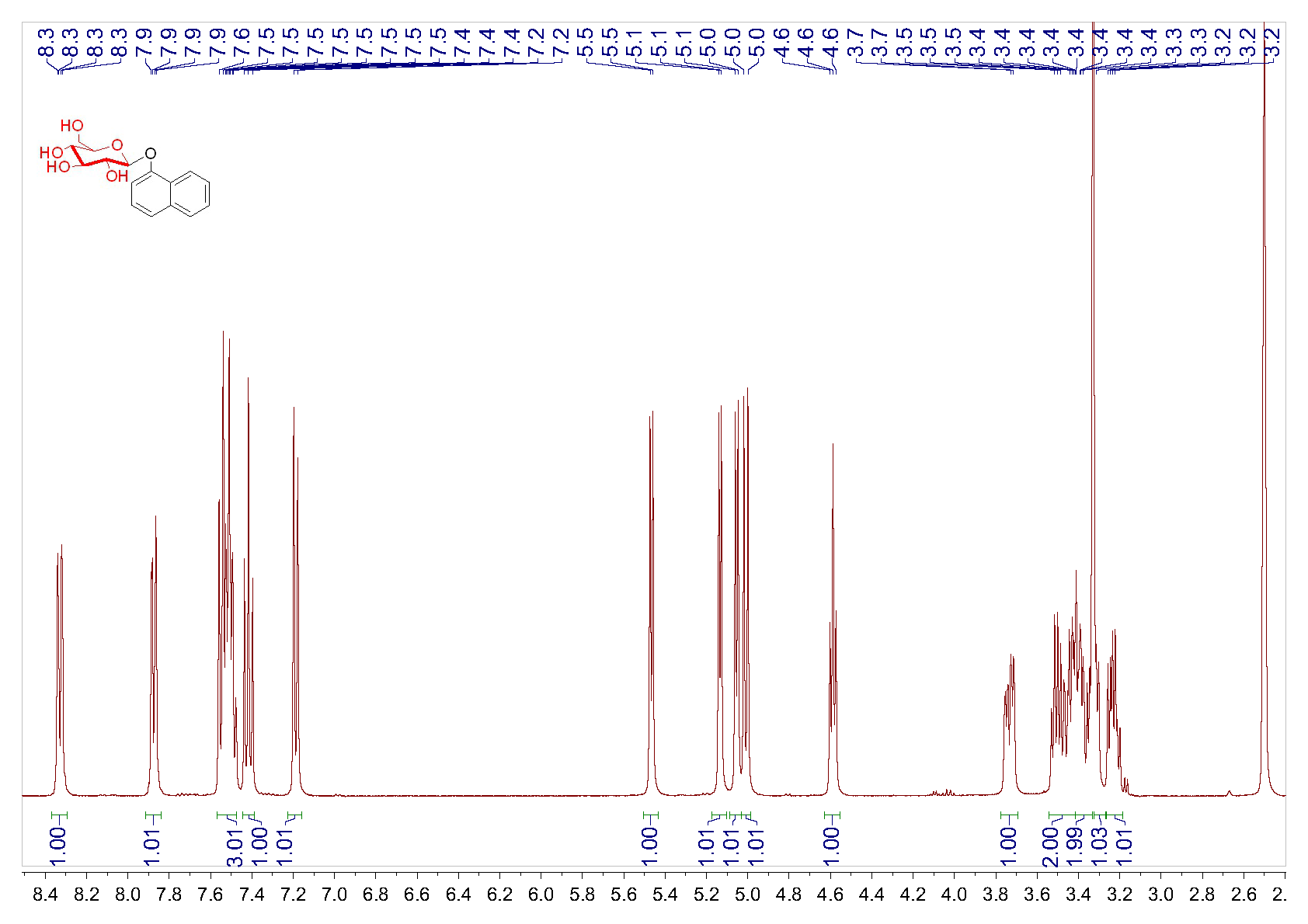
**

**Fig. S19.** ^1^H NMR spectrum of **1a** (400 MHz, DMSO-d_6_)


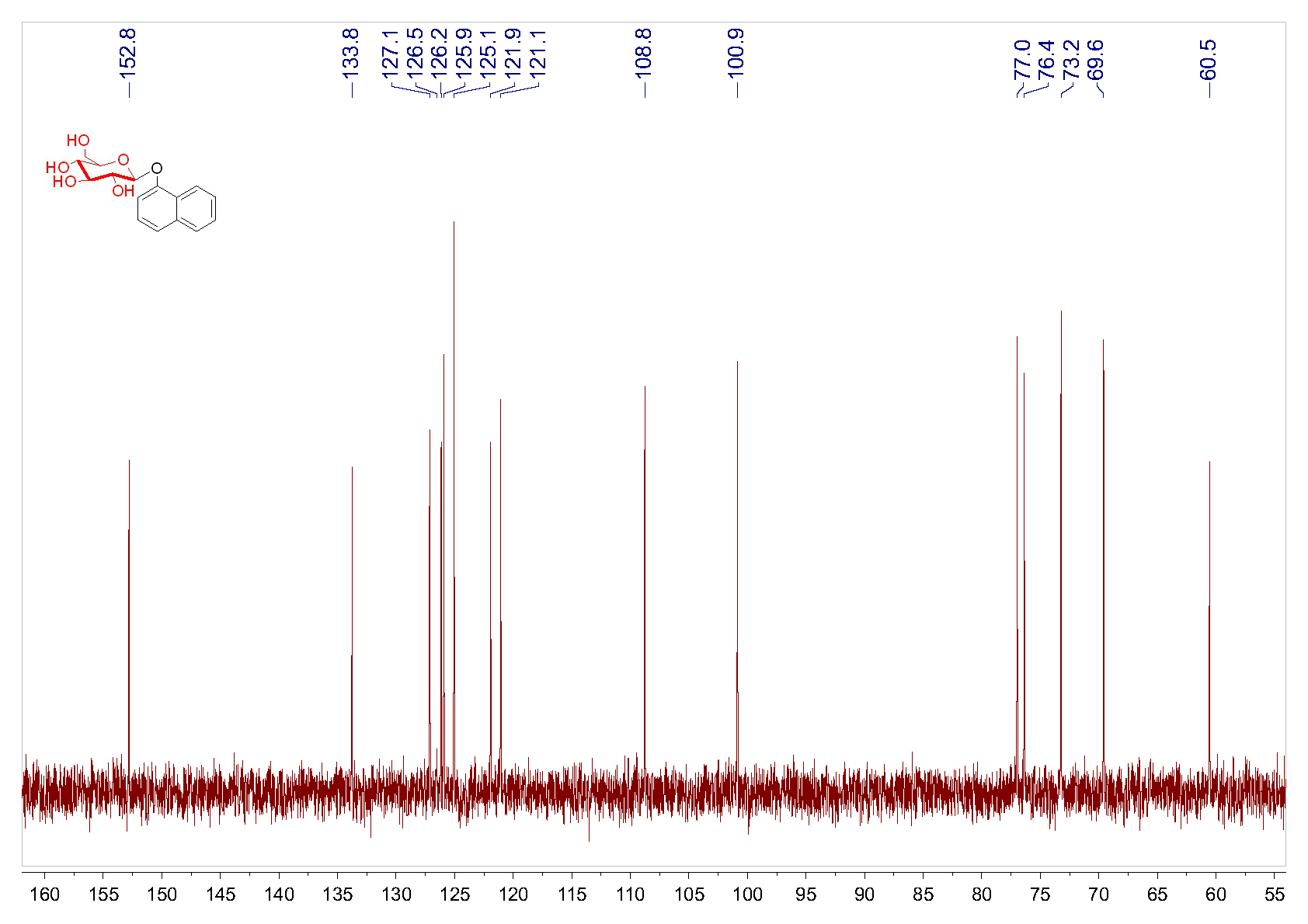


**Fig. S20.** ^13^C NMR spectrum of **1a** (101 MHz, DMSO-d_6_)

**
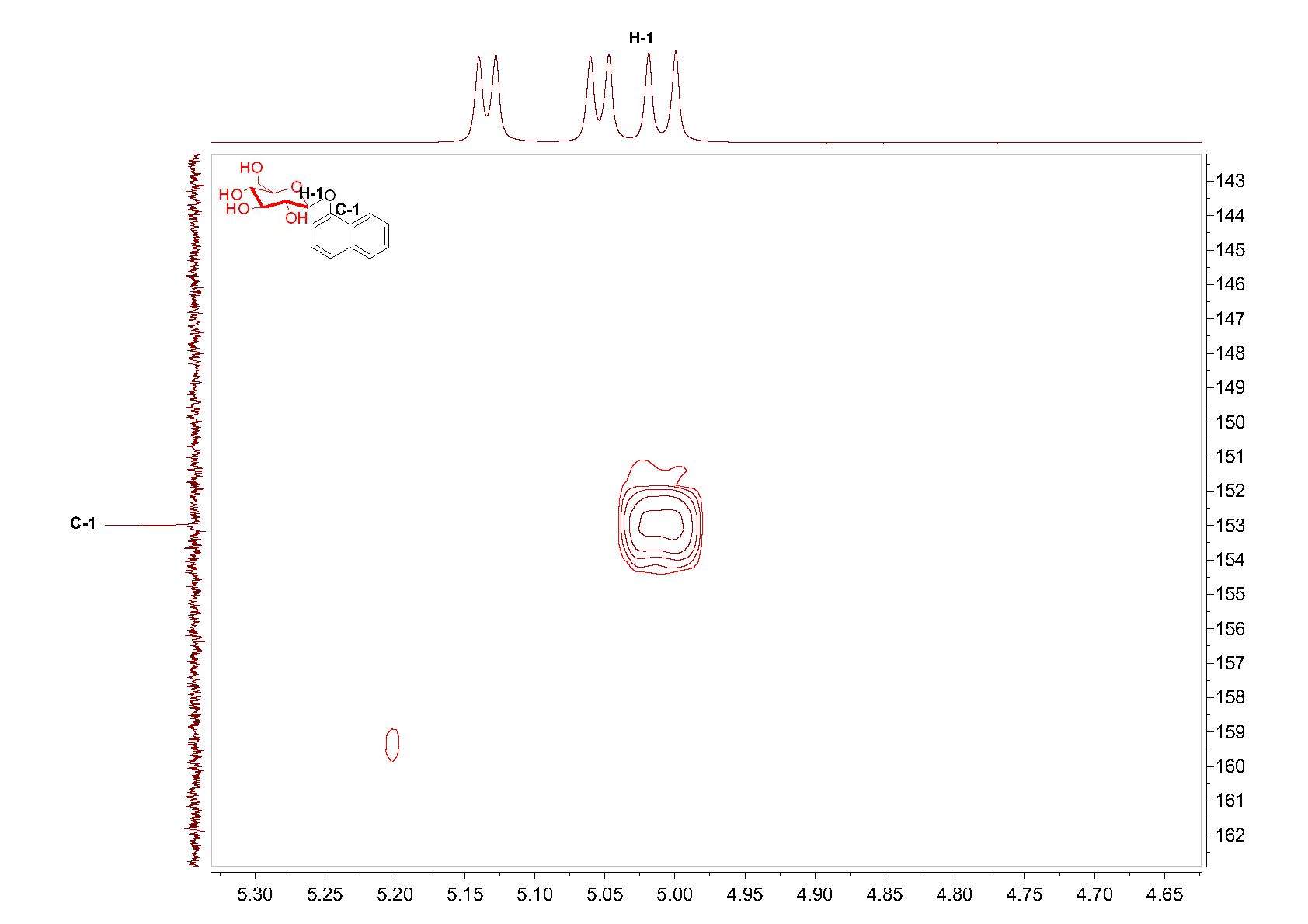
**

**Fig. S21.** HMBC spectrum of **1a** (400 MHz/101 MHz, DMSO-d_6_)

**
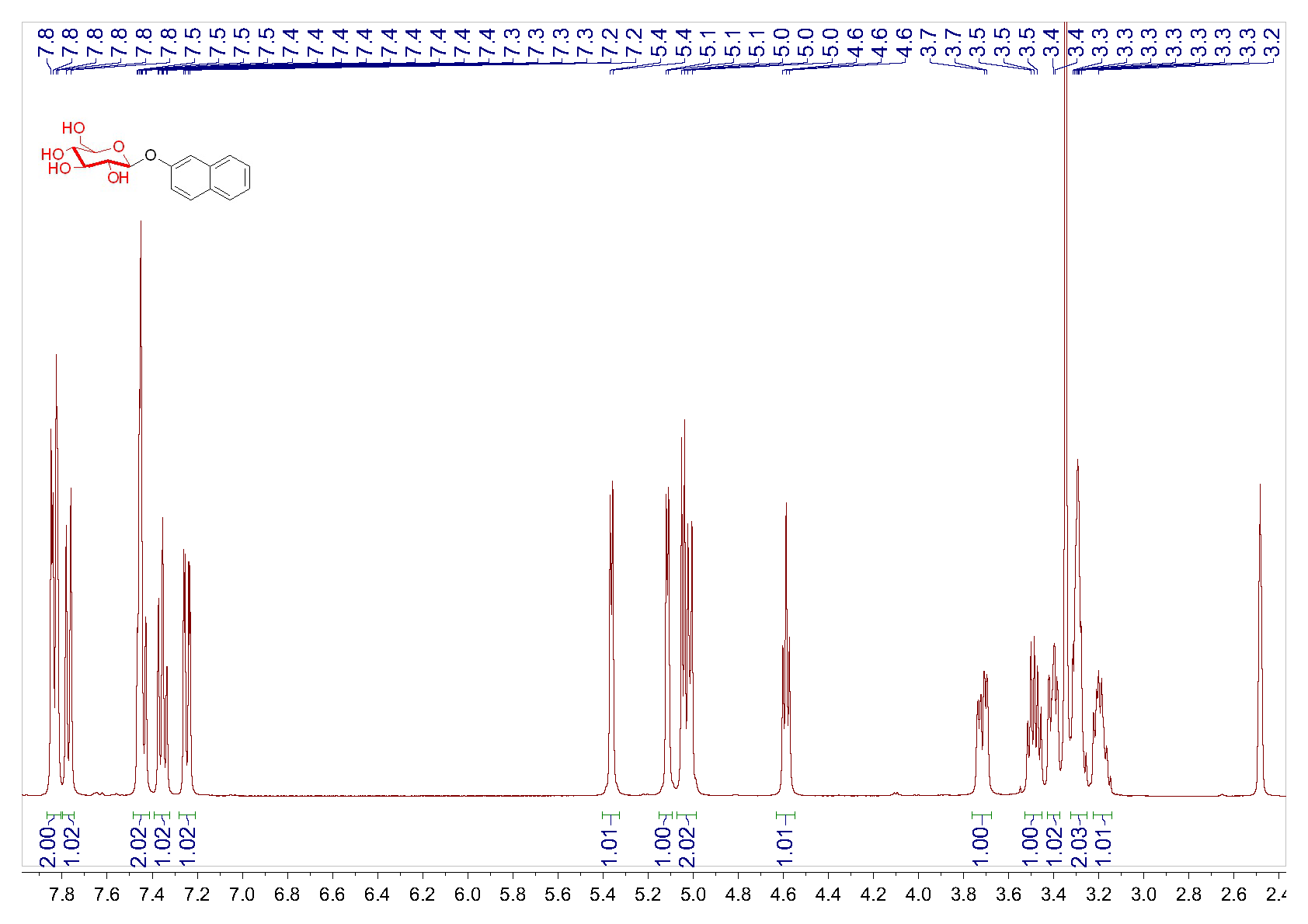
**

**Fig. S22.** ^1^H NMR spectrum of **2a** (400 MHz, DMSO-d_6_)

**
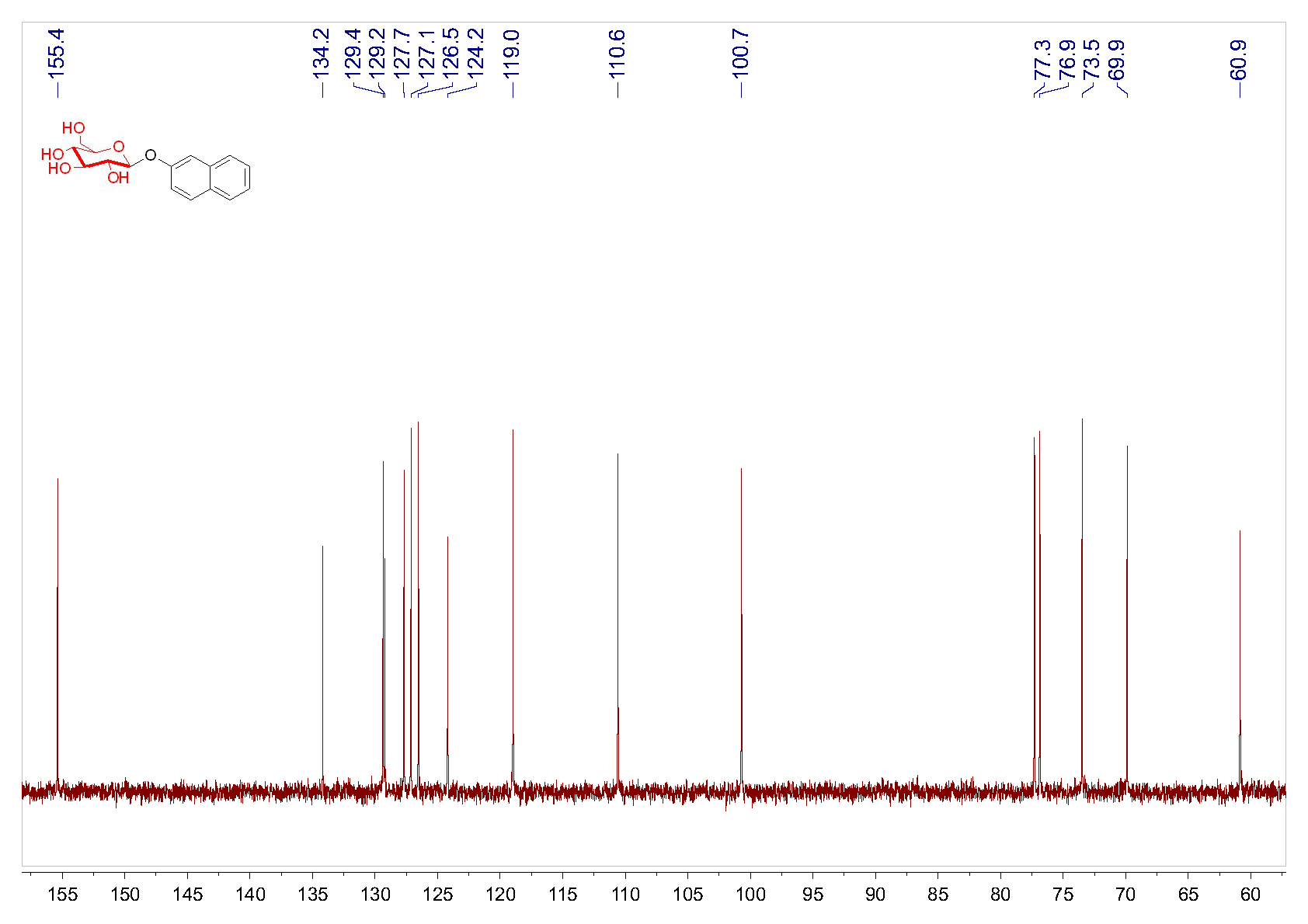
**

**Fig. S23.** ^13^C NMR spectrum of **2a** (101 MHz, DMSO-d_6_)

**
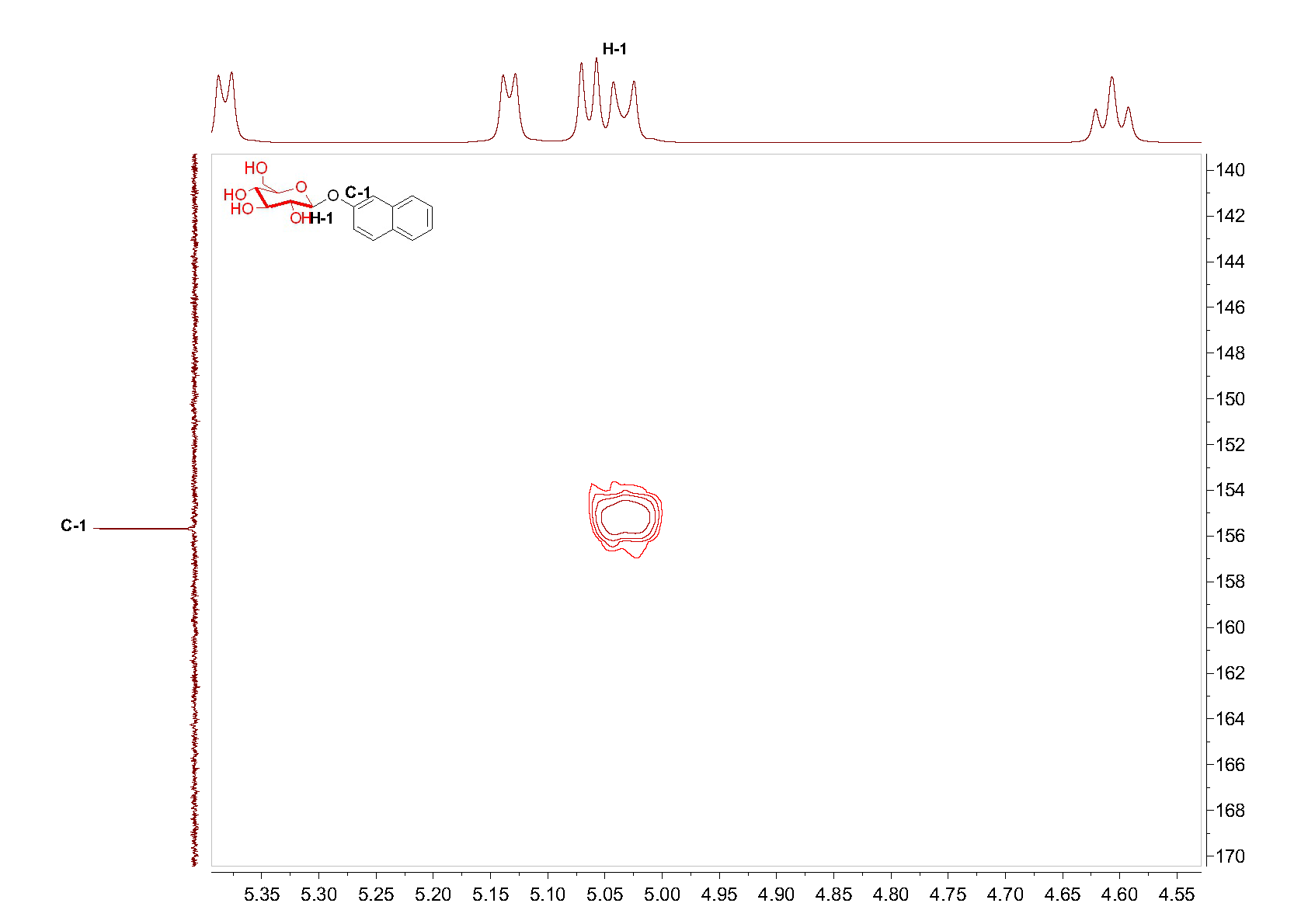
**

**Fig. S24.** HMBC spectrum of **2a** (400 MHz/101 MHz, DMSO-d_6_)


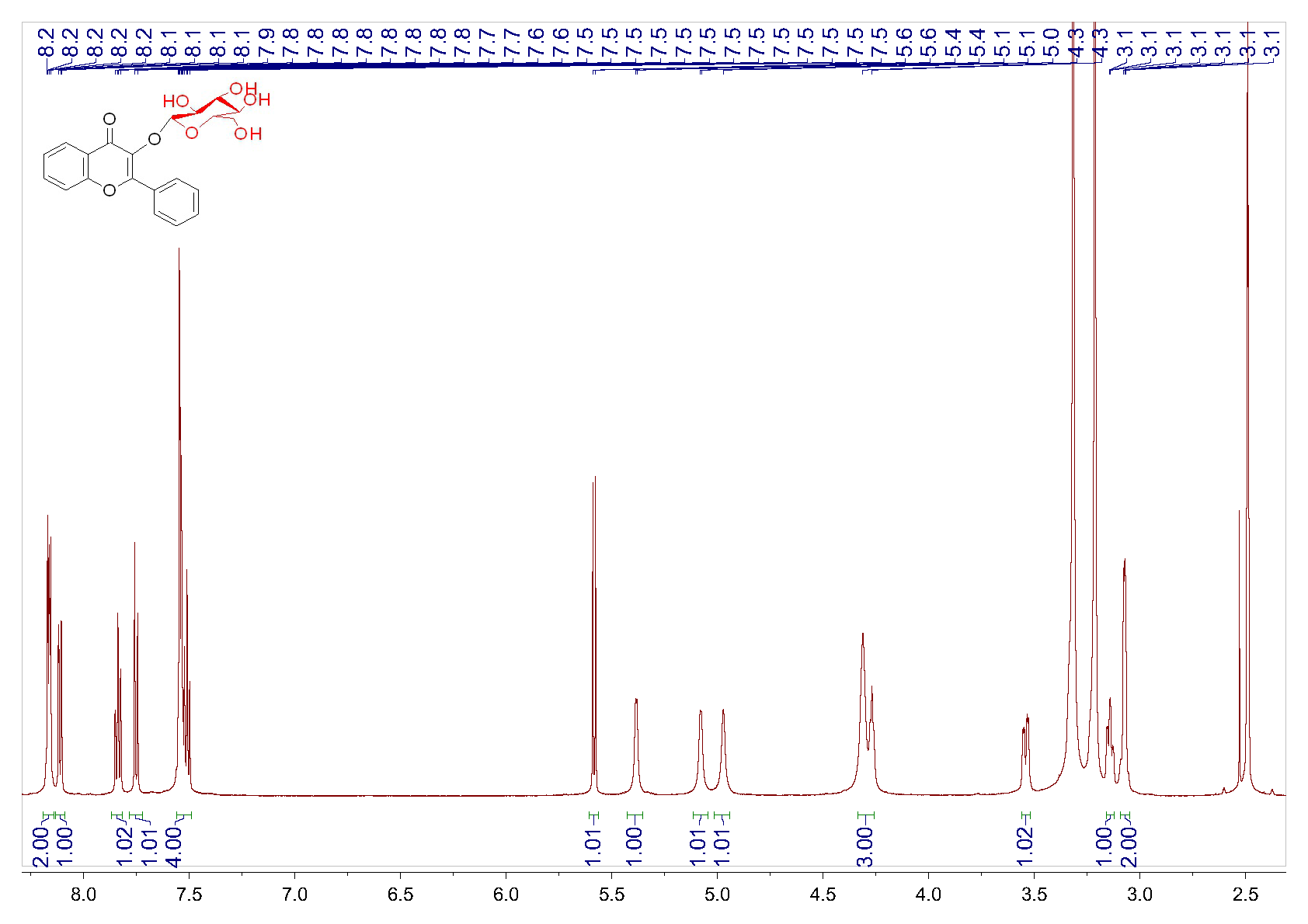


**Fig. S25.** ^1^H NMR spectrum of **3a** (400 MHz, DMSO-d_6_)


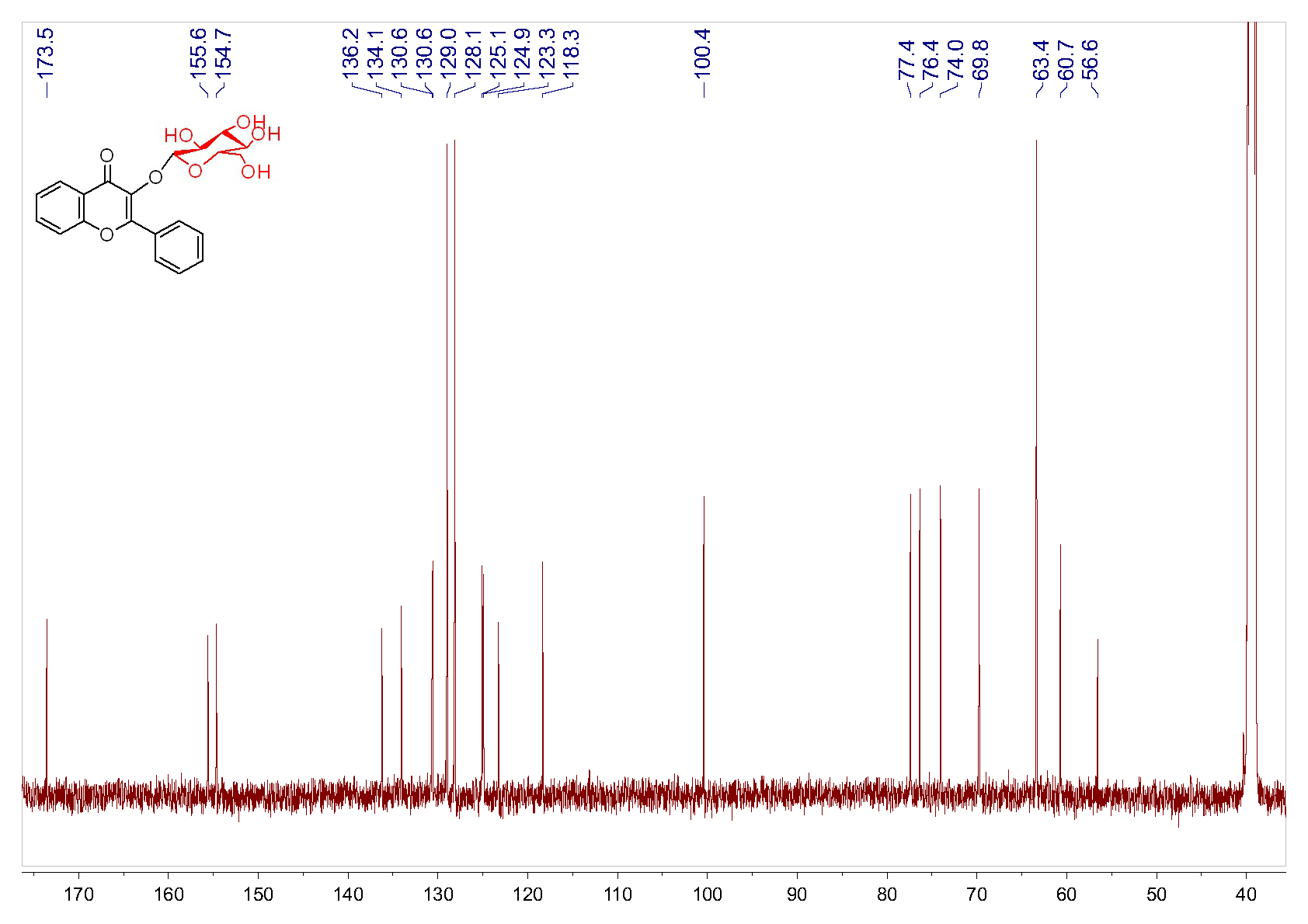


**Fig. S26.** ^13^C NMR spectrum of **3a** (101 MHz, DMSO-d_6_)

**
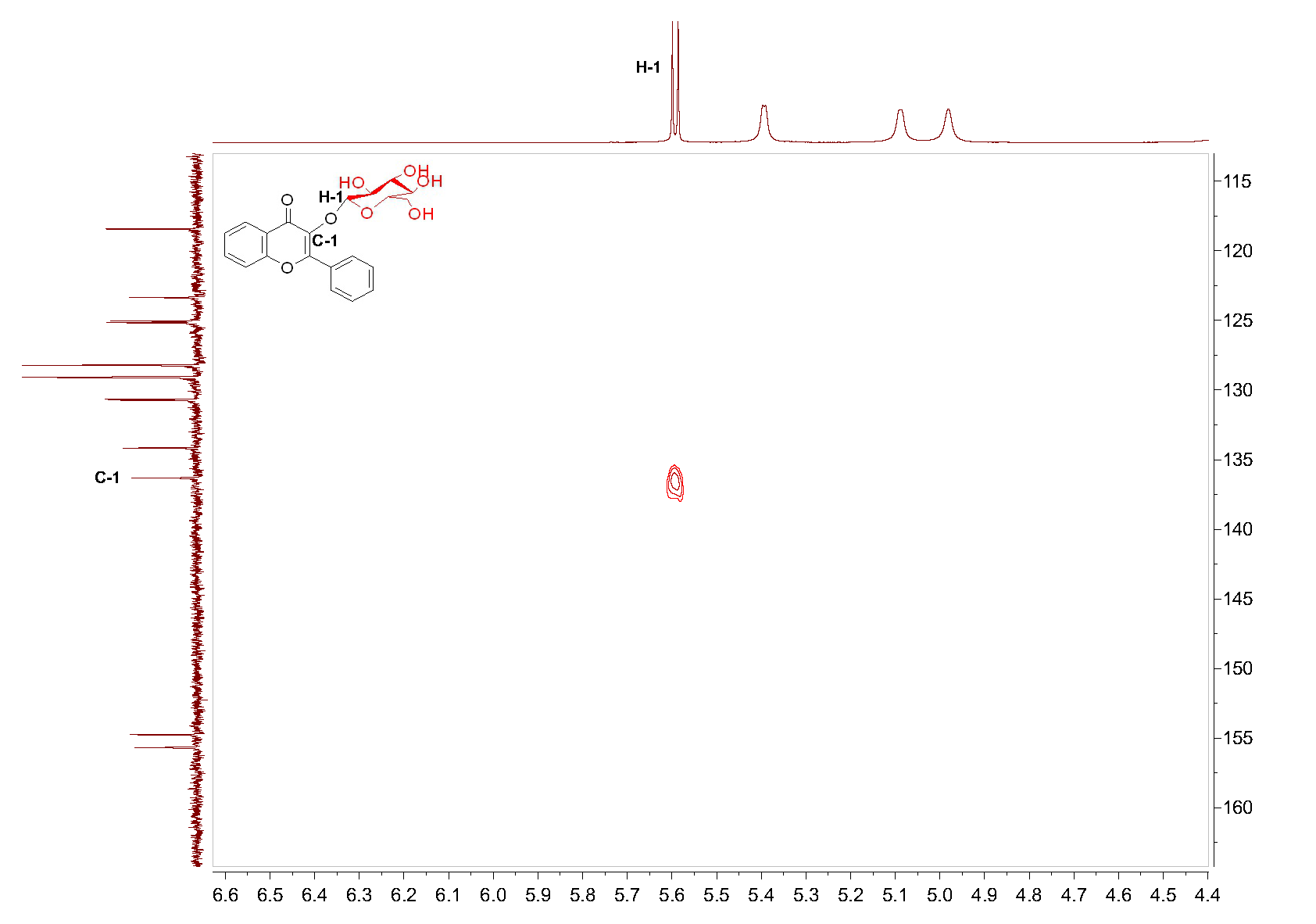
**

**Fig. S27.** HMBC spectrum of **3a** (400 MHz/101 MHz, DMSO-d_6_)

**
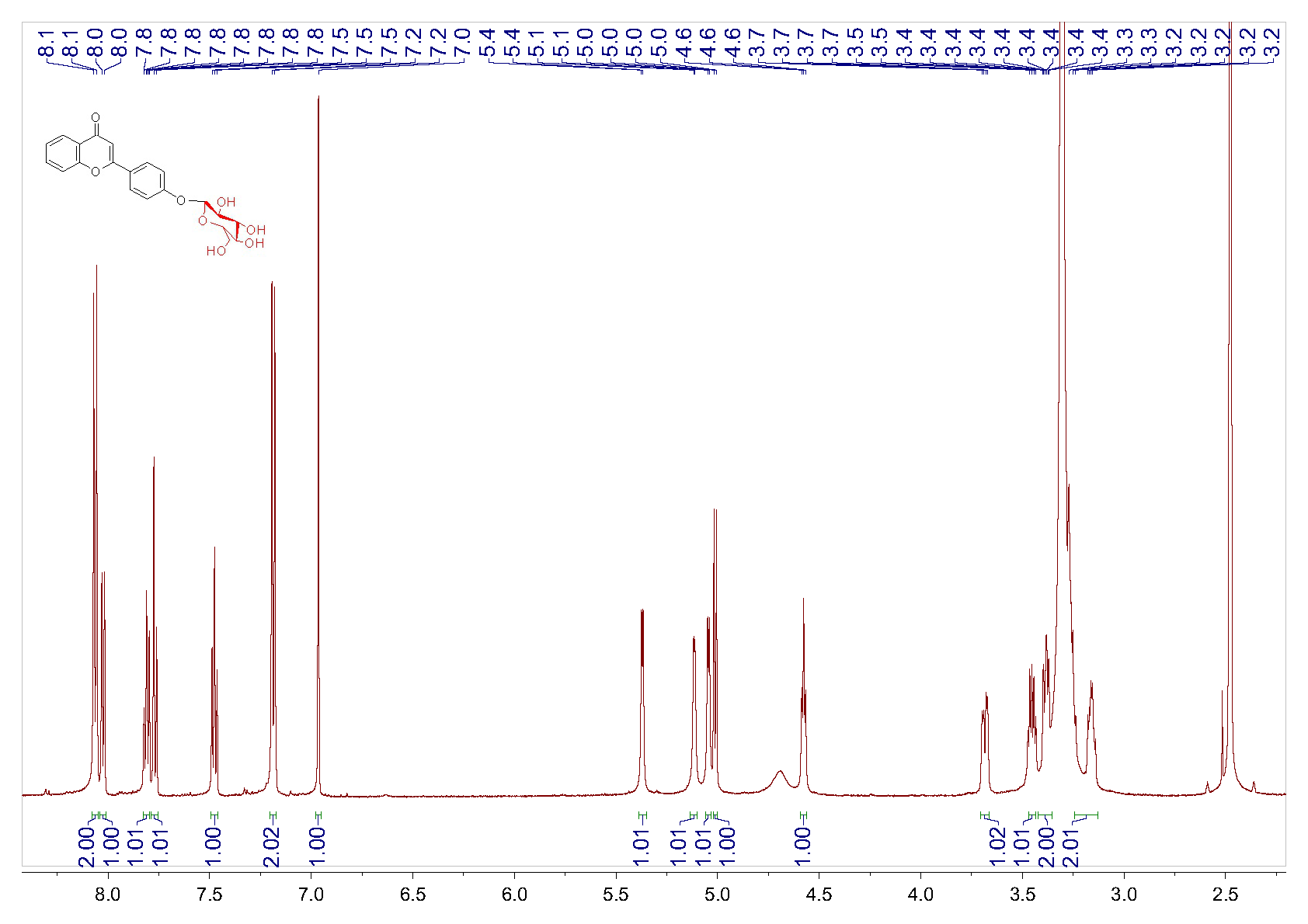
**

**Fig. S28.** ^1^H NMR spectrum of **4a** (400 MHz, DMSO-d_6_)

**
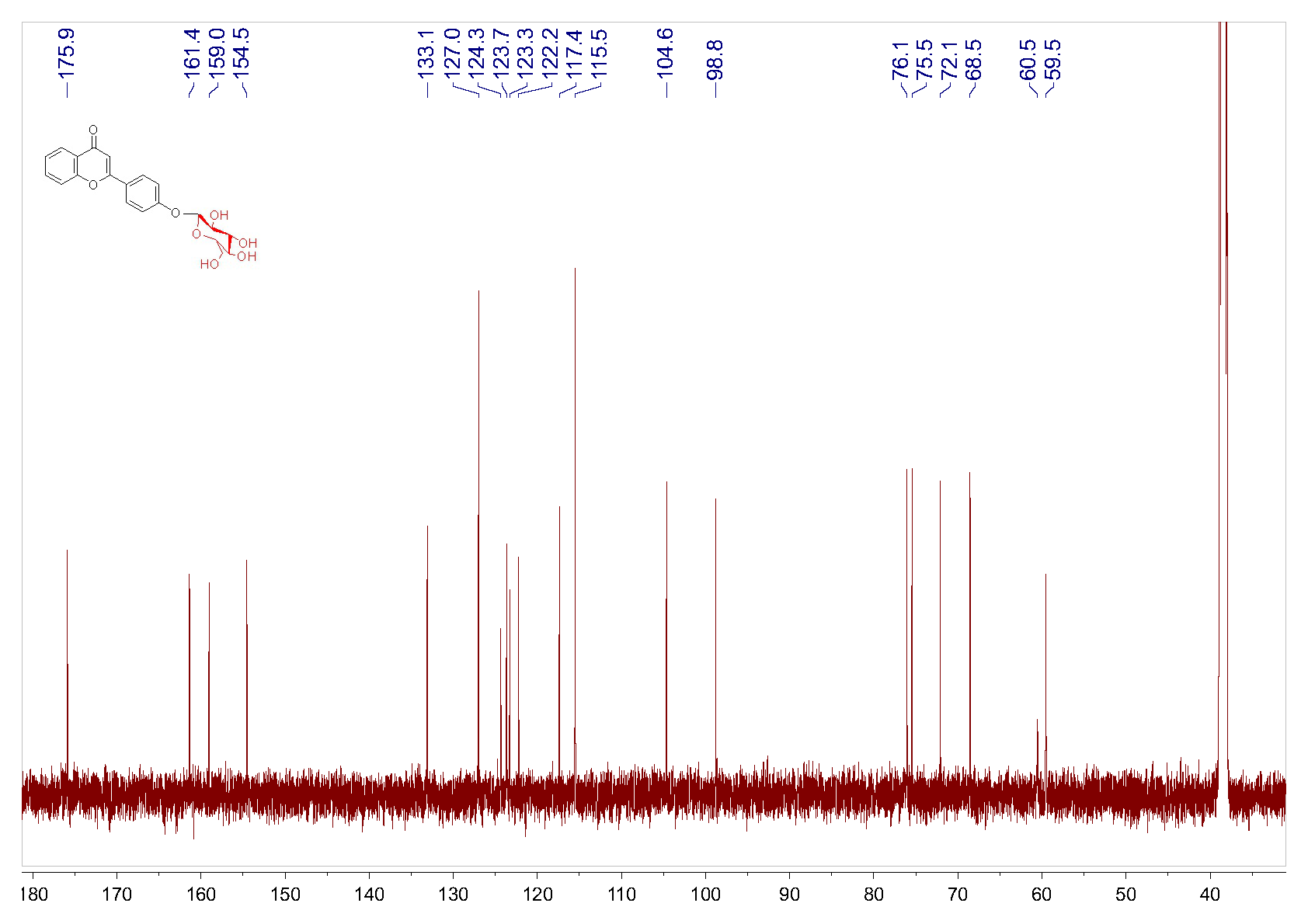
**

**Fig. S29.** ^13^C NMR spectrum of **4a** (101 MHz, DMSO-d_6_)

**
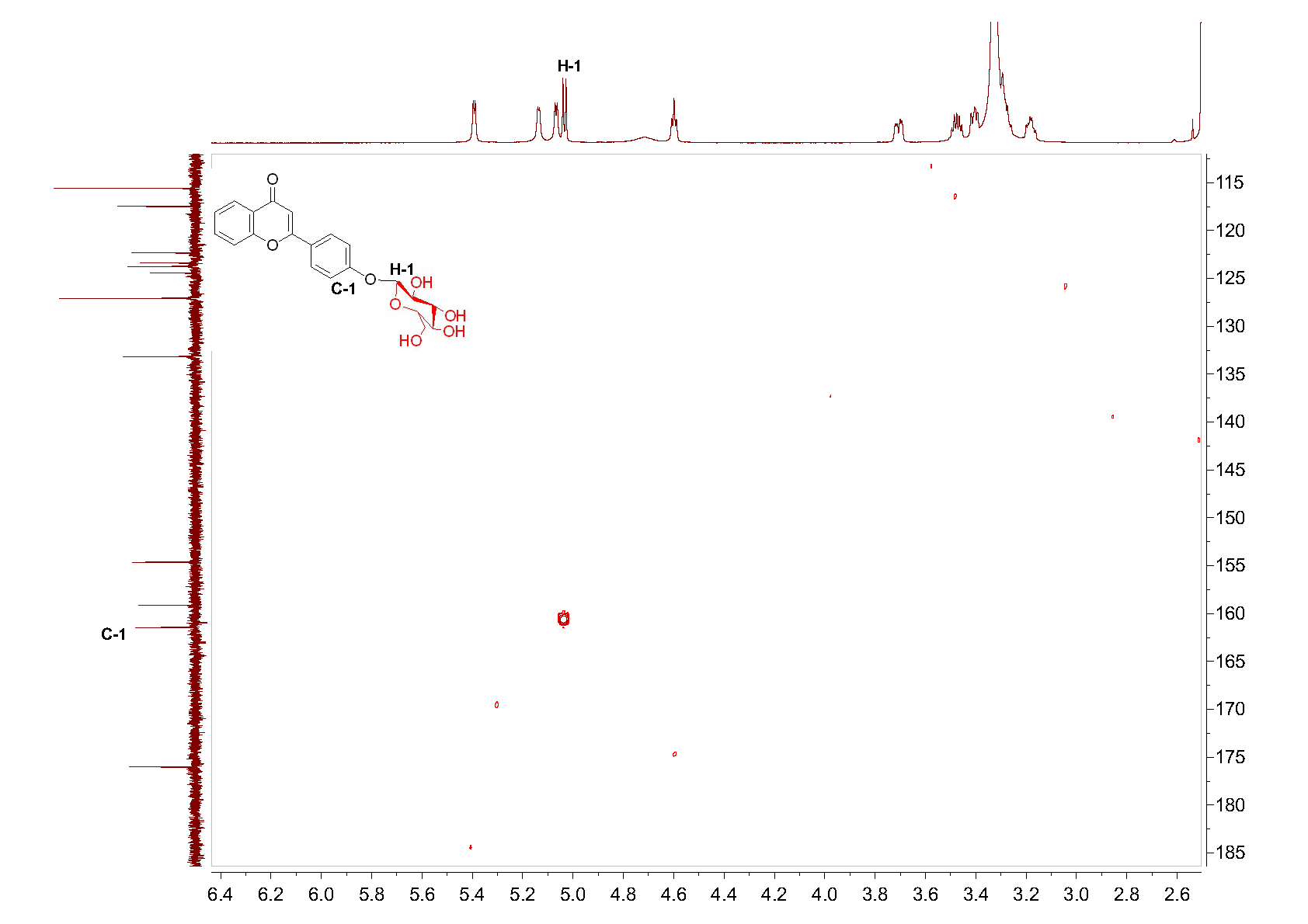
**

**Fig. S30.** HMBC spectrum of **4a** (400 MHz/101 MHz, DMSO-d_6_)


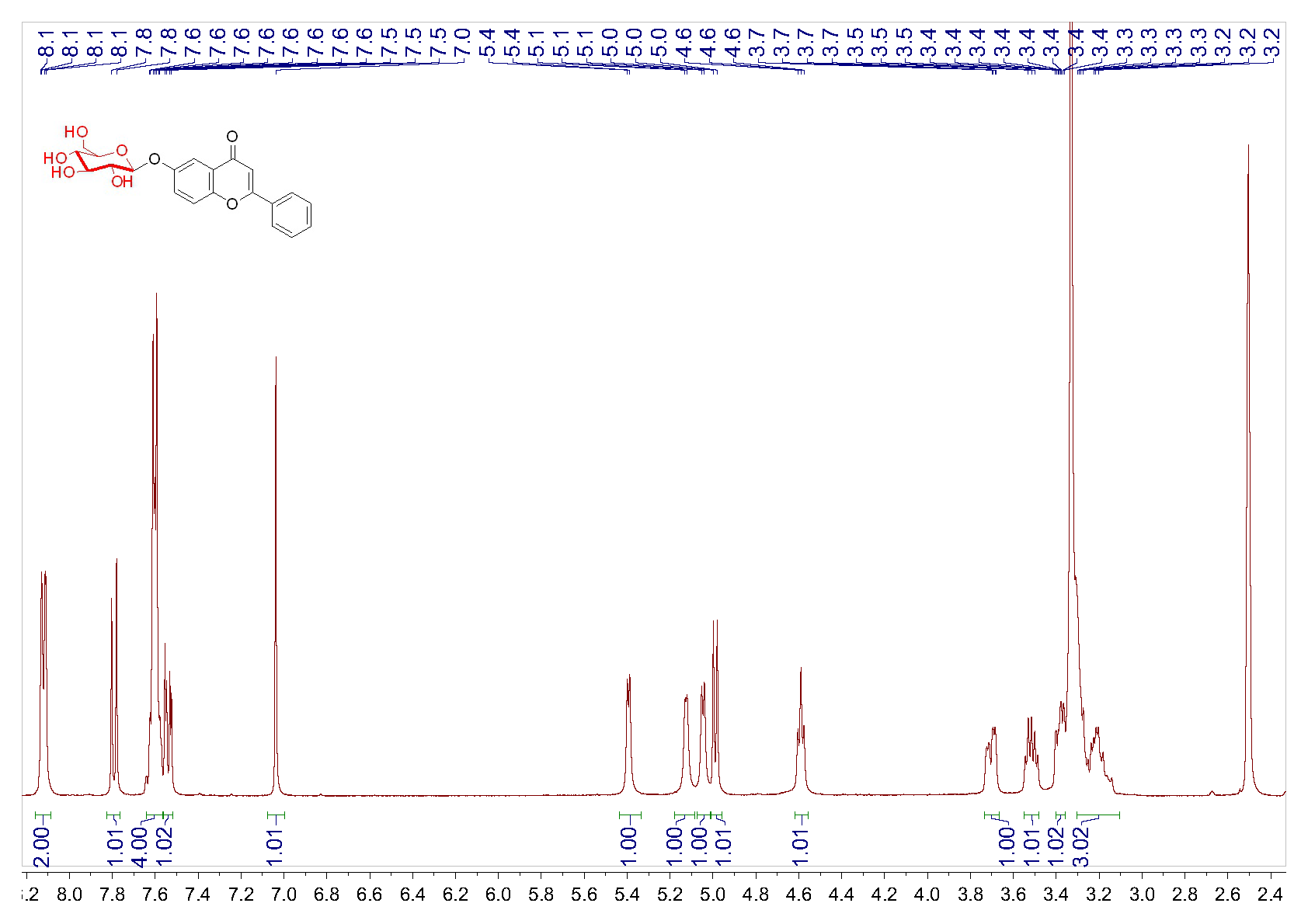


**Fig. S31.** ^1^H NMR spectrum of **5a** (400 MHz, DMSO-d_6_)


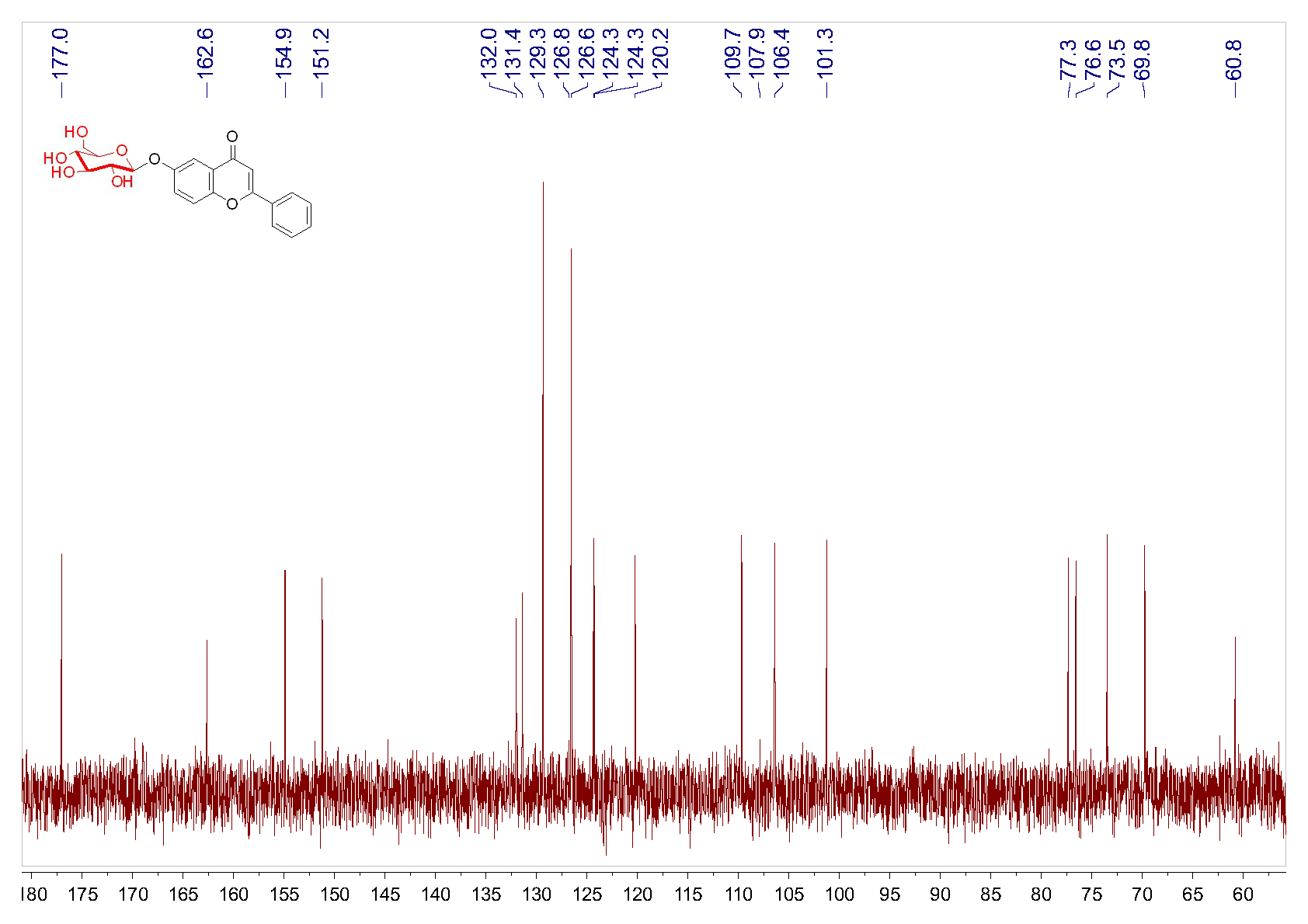


**Fig. S32.** ^13^C NMR spectrum of **5a** (101 MHz, DMSO-d_6_)

**
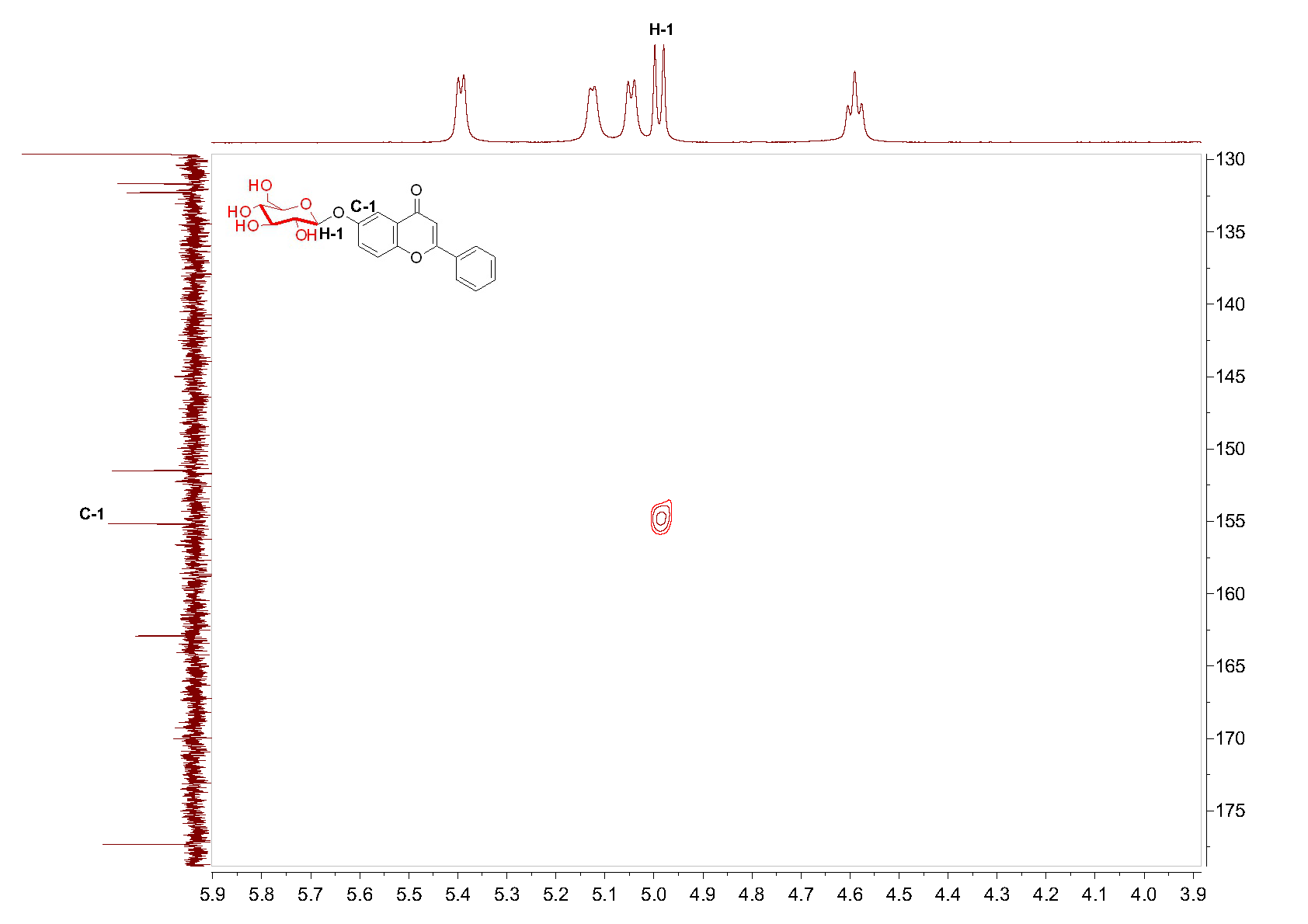
**

**Fig. S33.** HMBC spectrum of **5a** (400 MHz/101 MHz, DMSO-d_6_)


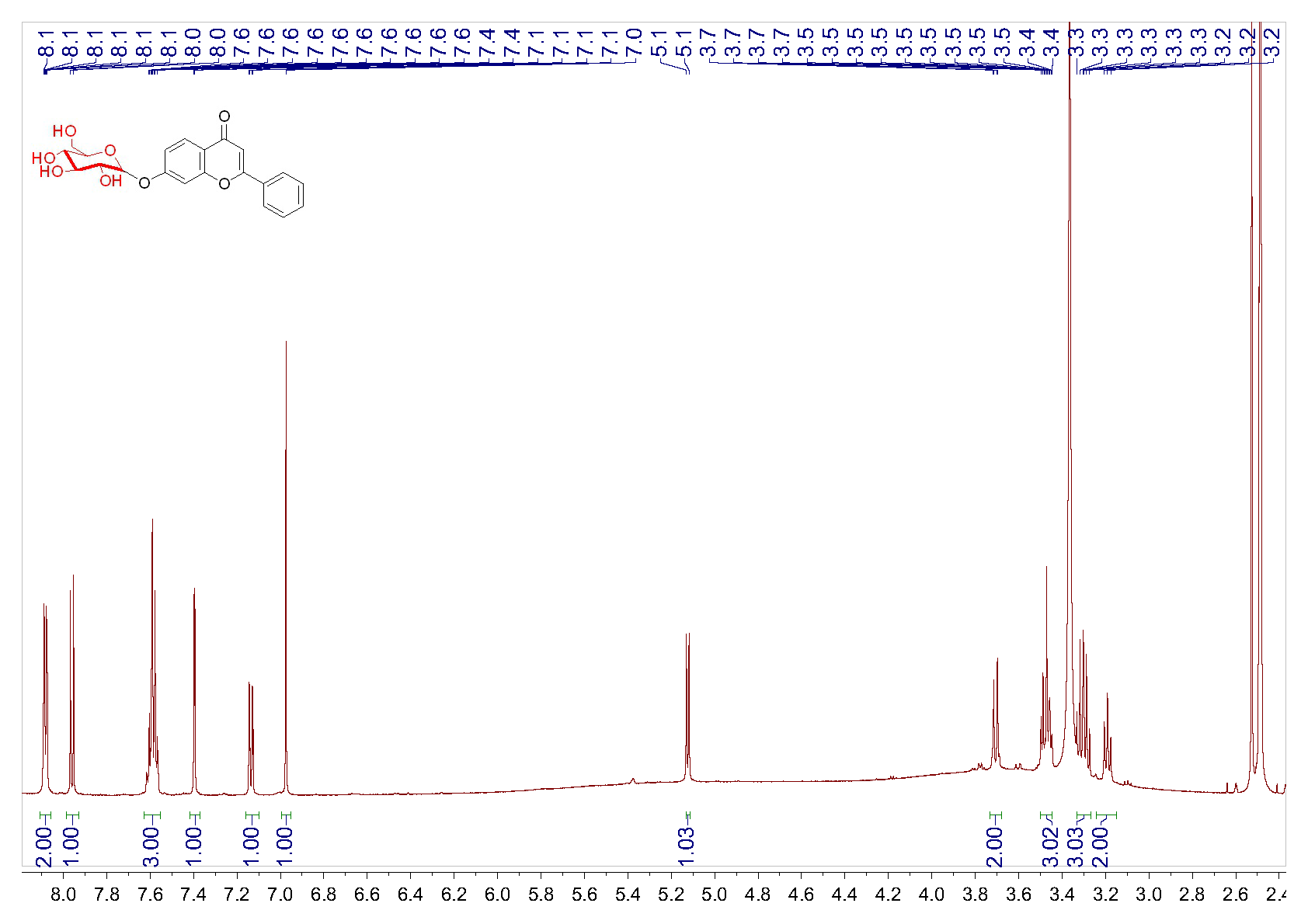


**Fig. S34.** ^1^H NMR spectrum of **6a** (600 MHz, DMSO-d_6_)


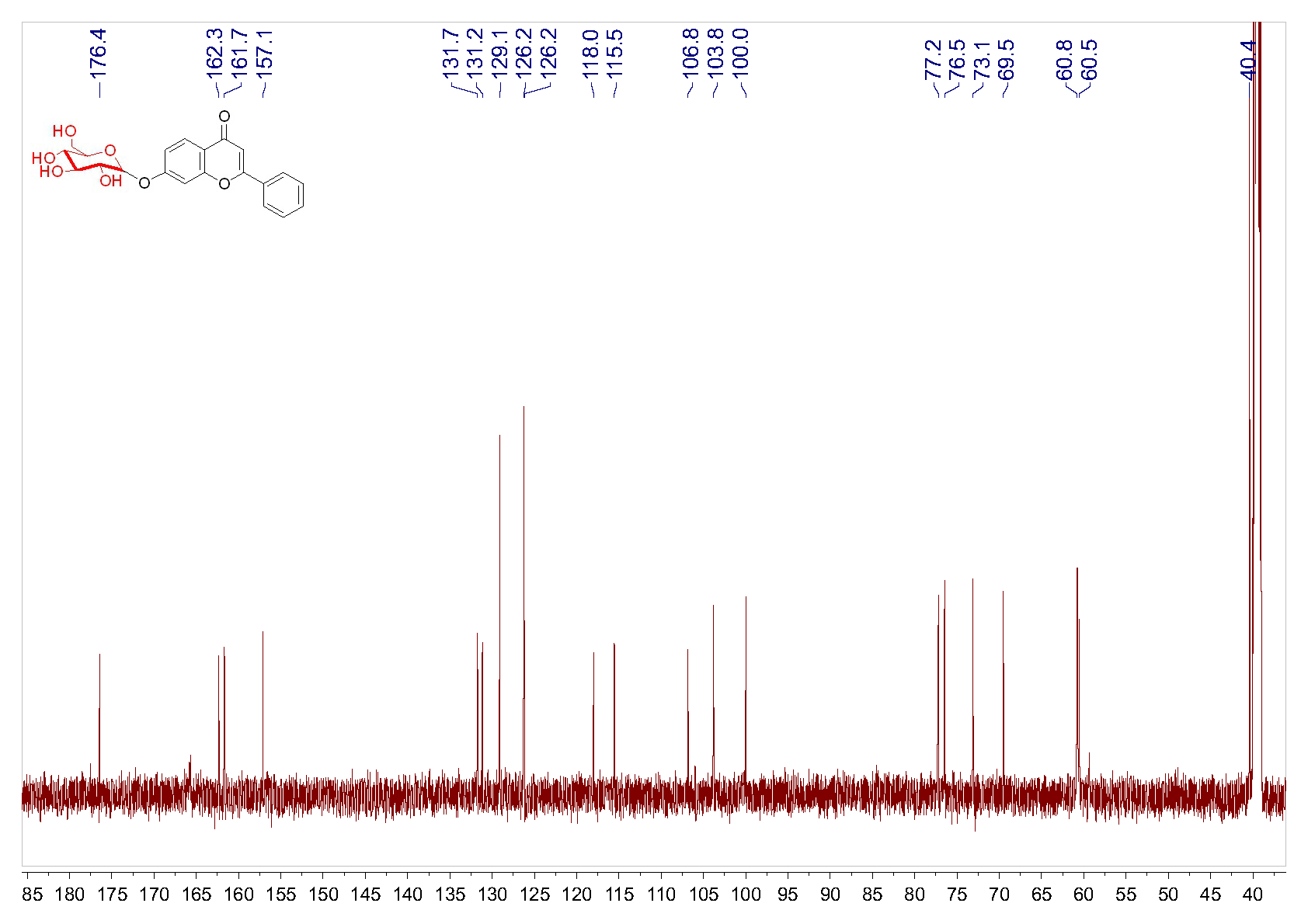


**Fig. S35.** ^13^C NMR spectrum of **6a** (151 MHz, DMSO-d_6_)

**
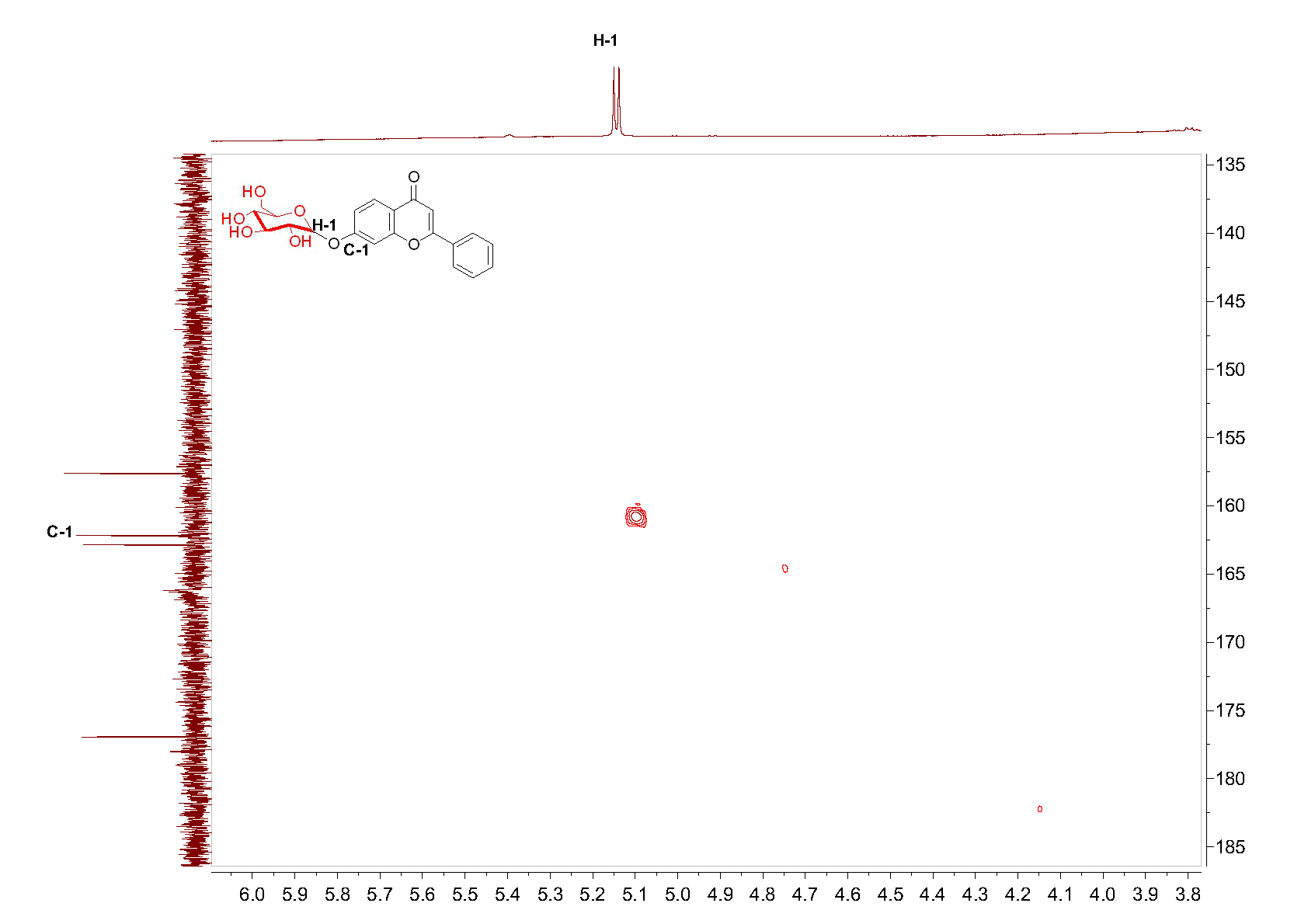
**

**Fig. S36.** HMBC spectrum of **6a** (600 MHz/151 MHz, DMSO-d_6_)


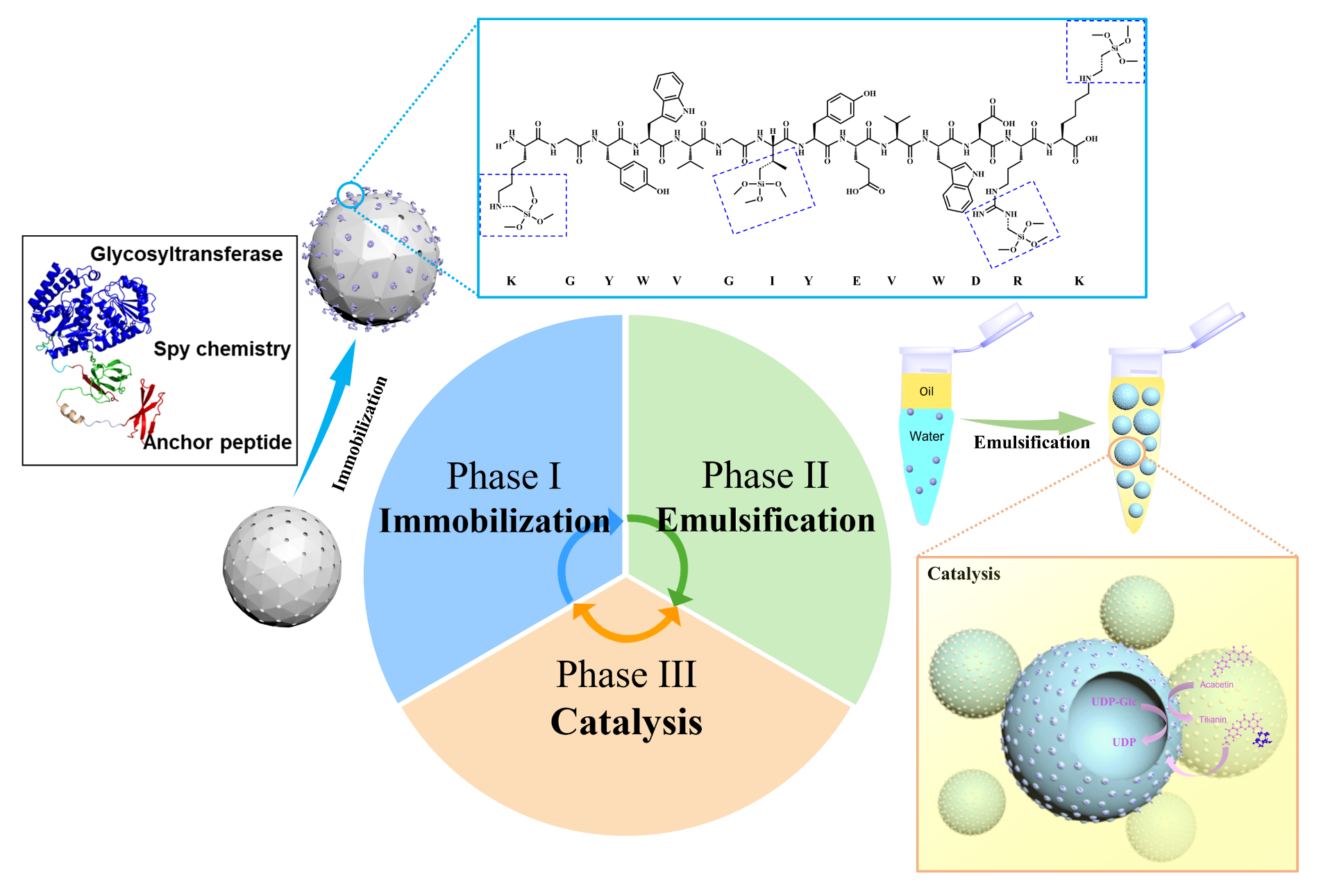


## Fig. S37. The integration of Spy chemistry with anchor peptide-based immobilization for enhancing enzyme robustness and catalytic performance in three phases. Phase I: The enzyme immobilization was carried out in 1.5 mL tube with shaker. 500 µL purified enzyme (1 mM) was incubated with 5 mg carriers of mesoporous (CM). Phase II: The collected immobilized enzyme (~ 2 mg) was applied in the construction of Pickering emulsion (200 µL) for the interfacial catalysis. The immobilized enzyme worked as the emulsifier and catalyst. The buffer (50 mM Tris–HCl, pH 7.5, 10 mM MgCl_2_, with 4 mM UDP-sugars) was viewed as water-phase. The organic solvent 2-hexanone (with 2 mM acacetin) worked as oil phase. Phase III: The Pickering emulsion interfacial catalysis occurred effectively, and the continuous transfer of glycoside products (aca-Glc, tilianin) from the oil phase to the water phase prevented the reaction from reaching equilibrium, thereby pushing the reaction forward.

# 4. The DNA and protein sequences of GTs with restriction sites.

**>BarGT-3**

Vector: pET-28a(+)

Restriction sites: NdeI and NotI

**DNA sequence:**

CATATGGCGAACGTGCTGGTGATTAACTTTCCGGGCGAAGGCCATATTAACCCGACCCTGGCGATTGTGAGCGAACTGATTCAGCGCGGCGAAACCGTGGTGAGCTATTGCATTGAAGATTATCGCAAAAAAGTGGAAGCGACCGGCGCGGAATTTCGCGTGTTTGAAAACTTTCTGAGTCAGATTAACATTATGGAACGCGTGAACGAAGGCGGCAGCCCGCTGACCATGCTGAGCCACATGATTGAAGCGAGCGAACGCATTGTGACGCAGATTGTGGAAGAAACCAAAGAAGAAAAATATGATTATCTGATTTATGATAACCATTTTCCGGTGGGCCGCATTATTGCGAACATTCTGCATCTGCCGAGCGTGAGCAGCTGCACCACCTTTGCGGTGAATCAGTATATTAACTTTCATGATGAACAAGAAAGCCGCCAAGTGGATGAAATGGATCCGCTGTATCAGAGCTGCCTGGCGGGCATGGAACGCTGGAACAAACAGTATGGCATGAAATGCAACAGCATGTATGATATTATGAACCATCCGGGCGATATTACCATTGTGTATACGAGCAAAGAATATCAGCCGCGCAGCGAAGTGTTTGATGAAAGCTATAAATTTGTGGGCCCGAGCATTGCGACCCGCAAAGAAGTGGGCAGCTTTCCGACCGAAGATCTGAAAAACGAAAAAGTGATTTTTATTAGCATGGGCACCGTGTTTAACGAACAGCCGGCGCTGTATGAAAAATGCTTTGAAGCGTTTAAAGATGTGGATGCGACCGTGGTGCTGGTGGTGGGCAAAAAAATTAACATTAGTCAGTTTGAAAACATTCCGAAAAACTTTAAACTGTATAACTATGTGCCGCAGCTGGAAGTGCTGCAGCATGCGGATGTGTTTGTGACCCATGGCGGCATGAACAGCAGTAGCGAAGCGCTGTATTATGGCGTGCCGCTGGTGGTGATTCCGGTGACCGGCGATCAGCCGTTTGTGGCGAAACGCCTGACCGAAGTGGGCGCGGGCATTCGCCTGAACCGCAACGAACTGACGAGCGAACTGCTGCGCGAAGCGGTGAAAAAAGTGATGGATGATGTGACCTTTAAAGAAAACAGCCGCAAAGTGGGCGAAAGCCTGCGCAACGCGGGCGGCTATCAGCGCGCGGTGGAAGAAATTTTTAAACTGAAAATGAAACCGTATGTGAAAATTAAATAAGCGGCCGC

**Protein sequence (QEL68377)**

MANVLVINFPGEGHINPTLAIVSELIQRGETVVSYCIEDYRKKVEATGAEFRVFENFLSQINIMERVNEGGSPLTMLSHMIEASERIVTQIVEETKEEKYDYLIYDNHFPVGRIIANILHLPSVSSCTTFAVNQYINFHDEQESRQVDEMDPLYQSCLAGMERWNKQYGMKCNSMYDIMNHPGDITIVYTSKEYQPRSEVFDESYKFVGPSIATRKEVGSFPTEDLKNEKVIFISMGTVFNEQPALYEKCFEAFKDVDATVVLVVGKKINISQFENIPKNFKLYNYVPQLEVLQHADVFVTHGGMNSSSEALYYGVPLVVIPVTGDQPFVAKRLTEVGAGIRLNRNELTSELLREAVKKVMDDVTFKENSRKVGESLRNAGGYQRAVEEIFKLKMKPYVKIK

**>BpsGT**

Vector: pET-28a(+)

Restriction sites: NdeI and NotI

**DNA sequence:**

CATATGGCGAATGTACTTGTAATAAATTTTCCTGGAGAGGGGCATATCAACCCGACATTAGCAGTTGTAAGTGAATTAGTACACAGAGGAGAAAAAGTTGTTTCGTATTGTATAGAGGACTATAGAAATAAAATTGAAGCAACTGGTGTGGAATTTCGAGCATATGAAAATTTTCTCCCACAAATCAATATTATGAATCGAATTAATGAAGGCAATAGTCCTGTCGAAATGTTGTCCAATATGATTGCAGCTACAGATCGTATTGTAAACCAGATTTTAGAGGAAATAAAGGAAGAACAATATGATTATATTATATATGATAATCATTTCGCAGTAGGTCGCATTATAGCAACTGTTTTACAGTTACCTAAAATTTCTTCTTGTACAACATTTGCTTTTAATAATTACATCACTTTTAATGAAGAATCTGAATCGAGAAAAGTAGATGAAAATTCTCCATTATATCAATCATGTTTATTAGGAATGGAACGATGGGAAGAGAAGTATGGGATTAAGTGTAATAACTTGTATGATATTATGAATCACCCTGGAGATATTACGATAGTATATACATCTAAGGAATATCAACCGTTTTCTCAAGTATTTAATGATTCTTTTAAATTCGTTGGGCCATCAATTGCAGTACGTAAAGAGGTAGGAAGCTTTCCAGTTGACATTTTAAAAAAGAAAGTTATTTTCGTTTCCATGGGGACAGTCTTTAATGAGCAACCGGATTTATATGAGAACTGTTTTGAAGCATTTAGAAATATGAATGCAATGGTCGTGTTAGTGGTCGGGAAAAGGATAAATATAGACCAATTTGAAAATATTCCAGAGAATTTTAAAGTGTTTAATTATGTACCTCAATTAGAAGTATTACAGCATACAGATGTATTTGTTACACATGGTGGTATGAACAGTTCTAGCGAAGCGTTATATTATGGTGTCCCGTTAGTTGTGATTCCAGTTACAGGGGATCAGCCATTAGTCGCGAAACGAGTCACTGAGGTGGGAGCCGGAGTTCAGCTTGATCGCAGAACATTAACTCCTGAAATGCTGCGTGAAGCAGTAGAAAAGGTAATGAATGATAAGGAATTTAAAGAAAATAGTCGTAAGATTGGAGAGTCTTTACGTGAATCTGGTGGCTATAAACAGGCAGTTGAGGAAATTGTAAAGTTCACTTCCAATTGTCGTGTGTAGGCGGCCGC

**Protein sequence (AJI18252)**

MANVLVINFPGEGHINPTLAVVSELVHRGEKVVSYCIEDYRNKIEATGVEFRAYENFLPQINIMNRINEGNSPVEMLSNMIAATDRIVNQILEEIKEEQYDYIIYDNHFAVGRIIATVLQLPKISSCTTFAFNNYITFNEESESRKVDENSPLYQSCLLGMERWEEKYGIKCNNLYDIMNHPGDITIVYTSKEYQPFSQVFNDSFKFVGPSIAVRKEVGSFPVDILKKKVIFVSMGTVFNEQPDLYENCFEAFRNMNAMVVLVVGKRINIDQFENIPENFKVFNYVPQLEVLQHTDVFVTHGGMNSSSEALYYGVPLVVIPVTGDQPLVAKRVTEVGAGVQLDRRTLTPEMLREAVEKVMNDKEFKENSRKIGESLRESGGYKQAVEEIVKFTSNCRV

**>BcyGT**

Vector: pET-28a(+)

Restriction sites: NdeI and NotI

**DNA sequence:**

CATATGGCGAAAGTTCTTGTAATTCATTTTCCGGGAGAAGGGCATATCAATCCGACATTGGCAGTGCTAAGTGAATTAATAAAAAGAGGCGAAAAAGTTGTTTCGTATTGTATTGAAGAGTATCGAAATAAAATTGAAACGACAGGTGTTGAATTTCGAATATATGAAAATTTTCTTCCTGAAATCAATATTATGGAGCGAATAAAACAAGGGAATAGCCCTCTAGAGATGTTGGCAAAGATGGTTGAAGCTACTGATCGTATTGTAGAGAATATTCTAGAGGAAATAAAAGAAGAACAATATGATTATTTAATTTATGATAATCATTTTGCAGTCGGATACATTTTGGCGAAAACCTTGCAGGTACCAAAAATTTCTTCTTGTACAACTTTTGCTTTTAATAAAGAAACCTCTCTTCATGATAAAAATGAACGTAGACAAATAGATGAAAGTTCTCCGCTTTATCAGTCTTGTGTATTAGGTATGAAAAAATGGGAAGAAAAATATGGAGTCAAATGCAATGACTTATTGGATATTATGAATCATCCAGGAGATATTACGATTGTCTATACGTCAAAACAATATCAACCTTACGCTCATACATTTGATGAATCATATAAATTTGTTGGACCTTCAATCGCACCGCGTAAAGATGTGGAAAGCTTTCCACTCAAATTTTCAAAAAAGAAGCCAGTCCTTTTTATTTCGATGGGAACGATTTTTAACCAACAACCAGAACTATATGAAACATGTTTCAAAGCATTTCGAGATTTAAATGTAACTGTGGTGTTAGTAGTGGGGAGGAAAATAAATATCAGTCAATTTGAAGAAATTCCAGCGAATTTTAATGTGTATAACTATGTACCACAATTAGAAATATTACAACATACAGATGTATTTATTACACATGGAGGAATGAATAGTTCTAGTGAGGCGTTATATTATGGAGTTCCACTTGTTGTCATTCCCGTTATGGGGGATCAACCTTTTGTGGCAAAACGAGTCGAAGAATTAGGAGCAGGAATTCAGTTGGATCGTACAAATATAACCCCTGAAATATTACGTGAAGCGGTAGAAATGATACTAGATAATAAAAGTTTTAGAGAAAAAAGTCGTAAAATAGGGGAATCTTTACACGCGGCAGGTGGATATAGACGAGCTGTCGATGAAATTGTAACATTCACCTCAGGTGCTATGCATAAATCATGCTTTCAATTAAAAAATAGTAAGTGAGCGGCCGC

**Protein sequence (AWC60795)**

MAKVLVIHFPGEGHINPTLAVLSELIKRGEKVVSYCIEEYRNKIETTGVEFRIYENFLPEINIMERIKQGNSPLEMLAKMVEATDRIVENILEEIKEEQYDYLIYDNHFAVGYILAKTLQVPKISSCTTFAFNKETSLHDKNERRQIDESSPLYQSCVLGMKKWEEKYGVKCNDLLDIMNHPGDITIVYTSKQYQPYAHTFDESYKFVGPSIAPRKDVESFPLKFSKKKPVLFISMGTIFNQQPELYETCFKAFRDLNVTVVLVVGRKINISQFEEIPANFNVYNYVPQLEILQHTDVFITHGGMNSSSEALYYGVPLVVIPVMGDQPFVAKRVEELGAGIQLDRTNITPEILREAVEMILDNKSFREKSRKIGESLHAAGGYRRAVDEIVTFTSGAMHKSCFQLKNSK

**>BacGT**

Vector: pET-28a(+)

Restriction sites: NdeI and NotI

**DNA sequence:**

CATATGGCAAAGGTACTAATGTTTAATTTTCCAGGGGAAGGACATGTGAACCCAACTCTTGCACTTATTGAAGAATTAGTAAAAAGAGGGGAAGAGGTTGTTTATTATTGTGTAGAGGAGTATAAGGAGAAAATTGAAAAAACAGGAGCTTTGTTTCGTCCATATGAAAATTTTTTAGCAAAAGTAGACATGTTGAAAAGGATGAATGGAGAAATAGATCCCTCTGAACTATTATTACATATGGTAAAATCCATGGATAAAATAATAAAGATTGTTATAGAAGAGTTGAAAGAAGAAAAGTATGATTACGTAATATATGATAACAATTTTGCAGTAGGATGGATCATAGCTGAAGCATTACATTTACCTAAAATCTCTTCATGCACAACATTTGCAGTTACGAAAAAGTTATTTAACGCATTGATGAACAATCATAATGAAGGAGACAAAAAATCACCTCTTTATCAAGAAGTTACGCATATTTTAGAAGAGTGGCAAAAGGTATATGGCTTTTCTATGAATGAAAAGAAAAATGTAATGACTTGTGCTGGAAATATTACTATCGTATATACGTCTGAATTTTTCCAACCTGATGTGGAGGAATTGGATGATTCTTATATCTTTGTTGGCCCCTCTATTACATCACGGAAAGATATGCAAGAAATTACTTTTAAACAGAAGGAAGAAGAGAAACTCATTTATATTTCTATGGGAACTGTATTTAATCAACAGATGGATTTTTATTACATATGTTTTGAAGCATTTAAAAATTTTCCTGCTACCATCATATTGTCCGTAGGAAAACATATTGACATCAACCAATTGAAAAATATCCCATCTAATTTCAAAGTTTATAATTATGTGCCTCAGTTAGAGGTATTGAAACAAGCTAATCTTTTTATTACGCATGGGGGAATGAATAGTTCTAGTGAAAGCTTATATTTTGGCGTTCCTATGATCGTTATCCCAGTTATGGGAGATCAACCGATAGTGGCCCAAAGAATAGAAGATTTAAAAGCTGGCATACAATTAAATCTAAAAAAGCTTACTCCAGTGACATTATATAATGCAGTAATGGAAGTATTATCGAATGATGTATACTTAGAAAATAGTCATAAAATCAAATGTACTTTAAAAAATGCTGGTGGATATATTAAAGCCGCTGAAACAATAGAACTATTTAAGTTGAAGACGGGTATTAAATAAGCGGCCGC

**Protein sequence (AIE79969)**

MAKVLMFNFPGEGHVNPTLALIEELVKRGEEVVYYCVEEYKEKIEKTGALFRPYENFLAKVDMLKRMNGEIDPSELLLHMVKSMDKIIKIVIEELKEEKYDYVIYDNNFAVGWIIAEALHLPKISSCTTFAVTKKLFNALMNNHNEGDKKSPLYQEVTHILEEWQKVYGFSMNEKKNVMTCAGNITIVYTSEFFQPDVEELDDSYIFVGPSITSRKDMQEITFKQKEEEKLIYISMGTVFNQQMDFYYICFEAFKNFPATIILSVGKHIDINQLKNIPSNFKVYNYVPQLEVLKQANLFITHGGMNSSSESLYFGVPMIVIPVMGDQPIVAQRIEDLKAGIQLNLKKLTPVTLYNAVMEVLSNDVYLENSHKIKCTLKNAGGYIKAAETIELFKLKTGIK

**>BceGT**

Vector: pET-28a(+)

Restriction sites: NdeI and NotI

**DNA sequence:**

CATATGGCAAAAGTTCTAATGTTTAATTTTCCAGGAGAAGGACATATTAATCCGACAATTGCACTTGTCGAAGAACTAATAAAAAGAGGCGAGGAAGTTATTTACTATTGCGTAGAAAAGTATAGAAGTAAAATAGAGAATACAGGAGCTTTGTTTCGACCATATGAAAATTTCATATCACAAATAGACATGGTAAAAAGAATGAGTGGGCAAATAGATTCTCAAGAACTATTATTACACATGATAAAATCCATGGATAAAATAATAGAGATAATTTTAGAAGAAGTAAAAGAAGAAGAATACGATTATGTTATATATGATAATAATTTCGCTGTTGGATGGATCATAGCAGAAGTATTACAATTACCTAAAATTTCTTCATGTACAACATTTGCCATTACTAAAAAGATAGCTTCTACTCTTATGAAAAATCATGGAGAAGAAAATAAAAAATCCCCTTTATATCAGGAAGTCATGTGTATTTTGAAAAAATGGGAAGATATTTATGGAATTACTCTTAATGAAAAACAGAATGTGATGACATGCCCAGGGGATATTACAATTGTATATACATCAAAATTGTATCAACCAAATGTCGAAGAATTTGATGATTCTTATATATTTGTCGGGCCCTCTATTGCACAGCGTAAAGATGTAGATTCTTTTTCCTTAGAACAGGAAAAGGGAAACAAGCTGATTTTTATTTCTATGGGAACAGTATTTAATCAGCAACCCGACTTTTATTACACATGTTTTGAAGCATTTCGAGATAGCTCGGTTACTGTCATATTAACTATAGGGGAACAAAATAATATTAGTCAATTAGATAATATTCCTTCTAATTTTAAGTTATATAATTACGTTCCTCAATTAGATATTCTACAACAAACTGATCTATTTATTACACACGGGGGAATGAATAGTTCCAGTGAAAGCTTATATTTTGGTGTTCCTATGCTAGTTATTCCAGTTATGGGAGATCAGCCTATAGTTGCTCAAAGAATAGAAGAATTAGAAGCCGGAGTCCAGTTAGATCGTAATTTGCTTACTCCGGAAATATTACGTAATACAGCAATGCATGTGTTATCGAATGATATTTATATAAAAAATAGCCATAGAATTGGAGAATCTTTAAAAAATGCTGGAGGATATAACAAGGCAGCCGATGAAATTGAAACCTTTAAATTTAAAATGGGTATTAAGTAAGCGGCCGC

**Protein sequence (QKH10521)**

MAKVLMFNFPGEGHINPTIALVEELIKRGEEVIYYCVEKYRSKIENTGALFRPYENFISQIDMVKRMSGQIDSQELLLHMIKSMDKIIEIILEEVKEEEYDYVIYDNNFAVGWIIAEVLQLPKISSCTTFAITKKIASTLMKNHGEENKKSPLYQEVMCILKKWEDIYGITLNEKQNVMTCPGDITIVYTSKLYQPNVEEFDDSYIFVGPSIAQRKDVDSFSLEQEKGNKLIFISMGTVFNQQPDFYYTCFEAFRDSSVTVILTIGEQNNISQLDNIPSNFKLYNYVPQLDILQQTDLFITHGGMNSSSESLYFGVPMLVIPVMGDQPIVAQRIEELEAGVQLDRNLLTPEILRNTAMHVLSNDIYIKNSHRIGESLKNAGGYNKAADEIETFKFKMGIK

**>BarGT-1**

Vector: pET-28a(+)

Restriction sites: NdeI and NotI

**DNA sequence:**

CATATGCTGAACATTCTGGTGGTGAACTTTCCGGCGGAAGGCCATGTGAACCCGACCCTGAACCTGGTGAAAGCGTTTACCAAACGCGGCGATAACGTGCATTATATTACCACCGCGAACTTTAAAGATCGCATTGAAGATCTGGGCGCGACCGTGCATATTCATCCGGATCTGCTGAAGGAAATTAGCATTGATGCGGAAACGAGCAGCGGCCTGAACGCGTTTTTTCATGTGCATGTGCAGACGAGCCTGTATATTCTGGAAATTACCAAACAGCTGTGCGAAAGCATTAACTTTGATTTTGTGATTTATGATATTTTTGGCGCGGGCGAACTGGTGAAAGAATATCTGCAAGTGCCGGGCGTGGTGAGCAGCCCGATTTTTCTGATTCCGCCGGAATTTCTGAAAACCCTGCCGTTTCATCCGAACGCGGATATGCCGTTTCAGCCGGAAGAAATTAGCGAAAAACTGCTGAATCAGATGGAACATAAATTTGGCGTGAAACCGAAAAACAACCTGCAGTTTATGAACAACAAAGGCGATGTGTGCCTGGTGTATACGAGCCGCTATTTTCAGCCGAACAACGAGAGCTTTGGCGAAAACAACATTTTTATTGGCCCGAGCATTAGCAAACGCAAAACCAACATTAAATTTCCGCTGGAAAGCCTGAAAGAAAAAAAAGTGATTTATATTAGCATGGGCACCCTGCTGGAAGGCCTGGAACCGTTTTTTAACACCTGCATTGATACCTTTAGCGATTTTGATGGCATTGTGGTGATGGCGATTGGCGATCGCAACGATATTAGCAAAATTAAAAAAGCGCCGGATAACTTTATTATTGCGCCGTATGTGCCGCAGAGCGAAATTCTGAACGAAGCGGATGTGTTTATTACCCATGGCGGCATGAACAGCGTGCATGATGCGATTCATTATAACGTGCCGTTTGTGATTATTCCGCATGATAAAGATCAGCCGATGATTGCGCAGCGCCTGACCGAACTGGAAGCGGCGCATCGCCTGCTGAAAGAACATGTGAACGTGCATACCCTGAAAGAAGCGGTGACCGATGTGCTGAGCAACGAAAAATATAAACATGGCATTCGCAAACTGAACGATAGCTTTCTGGAATGCGGCGGCAGCAAAGAAGCGATTGCGGTGATTGAAAGCCTGCTGAACAAAGTGAAACTGTAAGCGGCCGC

**Protein sequence (QEL68904)**

MLNILVVNFPAEGHVNPTLNLVKAFTKRGDNVHYITTANFKDRIEDLGATVHIHPDLLKEISIDAETSSGLNAFFHVHVQTSLYILEITKQLCESINFDFVIYDIFGAGELVKEYLQVPGVVSSPIFLIPPEFLKTLPFHPNADMPFQPEEISEKLLNQMEHKFGVKPKNNLQFMNNKGDVCLVYTSRYFQPNNESFGENNIFIGPSISKRKTNIKFPLESLKEKKVIYISMGTLLEGLEPFFNTCIDTFSDFDGIVVMAIGDRNDISKIKKAPDNFIIAPYVPQSEILNEADVFITHGGMNSVHDAIHYNVPFVIIPHDKDQPMIAQRLTELEAAHRLLKEHVNVHTLKEAVTDVLSNEKYKHGIRKLNDSFLECGGSKEAIAVIESLLNKVKL

**>Spy_BarGT-3**

Vector: pET-28a(+), SpyTag, Linkers, SpyCatcher

Restriction sites: NdeI and BamHI

**DNA sequence:**

CATATGCGTGGTGTACCACATATCGTGATGGTTGATGCGTACAAGCGTTATAAAGGATCTGGAGGATCTGGCGCGAACGTGCTGGTGATTAACTTTCCGGGCGAAGGCCATATTAACCCGACCCTGGCGATTGTGAGCGAACTGATTCAGCGCGGCGAAACCGTGGTGAGCTATTGCATTGAAGATTATCGCAAAAAAGTGGAAGCGACCGGCGCGGAATTTCGCGTGTTTGAAAACTTTCTGAGTCAGATTAACATTATGGAACGCGTGAACGAAGGCGGCAGCCCGCTGACCATGCTGAGCCACATGATTGAAGCGAGCGAACGCATTGTGACGCAGATTGTGGAAGAAACCAAAGAAGAAAAATATGATTATCTGATTTATGATAACCATTTTCCGGTGGGCCGCATTATTGCGAACATTCTGCATCTGCCGAGCGTGAGCAGCTGCACCACCTTTGCGGTGAATCAGTATATTAACTTTCATGATGAACAAGAAAGCCGCCAAGTGGATGAAATGGATCCGCTGTATCAGAGCTGCCTGGCGGGCATGGAACGCTGGAACAAACAGTATGGCATGAAATGCAACAGCATGTATGATATTATGAACCATCCGGGCGATATTACCATTGTGTATACGAGCAAAGAATATCAGCCGCGCAGCGAAGTGTTTGATGAAAGCTATAAATTTGTGGGCCCGAGCATTGCGACCCGCAAAGAAGTGGGCAGCTTTCCGACCGAAGATCTGAAAAACGAAAAAGTGATTTTTATTAGCATGGGCACCGTGTTTAACGAACAGCCGGCGCTGTATGAAAAATGCTTTGAAGCGTTTAAAGATGTGGATGCGACCGTGGTGCTGGTGGTGGGCAAAAAAATTAACATTAGTCAGTTTGAAAACATTCCGAAAAACTTTAAACTGTATAACTATGTGCCGCAGCTGGAAGTGCTGCAGCATGCGGATGTGTTTGTGACCCATGGCGGCATGAACAGCAGTAGCGAAGCGCTGTATTATGGCGTGCCGCTGGTGGTGATTCCGGTGACCGGCGATCAGCCGTTTGTGGCGAAACGCCTGACCGAAGTGGGCGCGGGCATTCGCCTGAACCGCAACGAACTGACGAGCGAACTGCTGCGCGAAGCGGTGAAAAAAGTGATGGATGATGTGACCTTTAAAGAAAACAGCCGCAAAGTGGGCGAAAGCCTGCGCAACGCGGGCGGCTATCAGCGCGCGGTGGAAGAAATTTTTAAACTGAAAATGAAACCGTATGTGAAAATTAAAGGATCCGGATCTGGAGGATCTGGCGTAACCACCTTATCAGGTTTATCAGGTGAGCAAGGTCCGTCCGGTGATATGACAACTGAAGAAGATAGTGCTACCCATATTAAATTCTCAAAACGTGATGAGGACGGCCGTGAGTTAGCTGGTGCAACTATGGAGTTGCGTGATTCATCTGGTAAAACTATTAGTACATGGATTTCAGATGGACATGTGAAGGATTTCTACCTGTATCCAGGAAAATATACATTTGTCGAAACCGCAGCACCAGACGGTTATGAGGTAGCAACTCCTATTGAATTTACAGTTAATGAGGACGGTCAGGTTACTGTAGATGGCGAAGCAACTGAAGGTGACGCTCATACTTAA

**Protein sequence**

HMRGVPHIVMVDAYKRYKGSGGSGANVLVINFPGEGHINPTLAIVSELIQRGETVVSYCIEDYRKKVEATGAEFRVFENFLSQINIMERVNEGGSPLTMLSHMIEASERIVTQIVEETKEEKYDYLIYDNHFPVGRIIANILHLPSVSSCTTFAVNQYINFHDEQESRQVDEMDPLYQSCLAGMERWNKQYGMKCNSMYDIMNHPGDITIVYTSKEYQPRSEVFDESYKFVGPSIATRKEVGSFPTEDLKNEKVIFISMGTVFNEQPALYEKCFEAFKDVDATVVLVVGKKINISQFENIPKNFKLYNYVPQLEVLQHADVFVTHGGMNSSSEALYYGVPLVVIPVTGDQPFVAKRLTEVGAGIRLNRNELTSELLREAVKKVMDDVTFKENSRKVGESLRNAGGYQRAVEEIFKLKMKPYVKIKGSGSGGSGVTTLSGLSGEQGPSGDMTTEEDSATHIKFSKRDEDGRELAGATMELRDSSGKTISTWISDGHVKDFYLYPGKYTFVETAAPDGYEVATPIEFTVNEDGQVTVDGEATEGDAHT

**>Spy_BarGT-3**

Vector: pET-28a(+), SpyTag, Linkers, SpyCatcher, **TEV protease cleavage site**, **LCI**

Restriction sites: NdeI and BamHI

**DNA sequence:**

CATATGCGTGGTGTACCACATATCGTGATGGTTGATGCGTACAAGCGTTATAAAGGATCTGGAGGATCTGGCGCGAACGTGCTGGTGATTAACTTTCCGGGCGAAGGCCATATTAACCCGACCCTGGCGATTGTGAGCGAACTGATTCAGCGCGGCGAAACCGTGGTGAGCTATTGCATTGAAGATTATCGCAAAAAAGTGGAAGCGACCGGCGCGGAATTTCGCGTGTTTGAAAACTTTCTGAGTCAGATTAACATTATGGAACGCGTGAACGAAGGCGGCAGCCCGCTGACCATGCTGAGCCACATGATTGAAGCGAGCGAACGCATTGTGACGCAGATTGTGGAAGAAACCAAAGAAGAAAAATATGATTATCTGATTTATGATAACCATTTTCCGGTGGGCCGCATTATTGCGAACATTCTGCATCTGCCGAGCGTGAGCAGCTGCACCACCTTTGCGGTGAATCAGTATATTAACTTTCATGATGAACAAGAAAGCCGCCAAGTGGATGAAATGGATCCGCTGTATCAGAGCTGCCTGGCGGGCATGGAACGCTGGAACAAACAGTATGGCATGAAATGCAACAGCATGTATGATATTATGAACCATCCGGGCGATATTACCATTGTGTATACGAGCAAAGAATATCAGCCGCGCAGCGAAGTGTTTGATGAAAGCTATAAATTTGTGGGCCCGAGCATTGCGACCCGCAAAGAAGTGGGCAGCTTTCCGACCGAAGATCTGAAAAACGAAAAAGTGATTTTTATTAGCATGGGCACCGTGTTTAACGAACAGCCGGCGCTGTATGAAAAATGCTTTGAAGCGTTTAAAGATGTGGATGCGACCGTGGTGCTGGTGGTGGGCAAAAAAATTAACATTAGTCAGTTTGAAAACATTCCGAAAAACTTTAAACTGTATAACTATGTGCCGCAGCTGGAAGTGCTGCAGCATGCGGATGTGTTTGTGACCCATGGCGGCATGAACAGCAGTAGCGAAGCGCTGTATTATGGCGTGCCGCTGGTGGTGATTCCGGTGACCGGCGATCAGCCGTTTGTGGCGAAACGCCTGACCGAAGTGGGCGCGGGCATTCGCCTGAACCGCAACGAACTGACGAGCGAACTGCTGCGCGAAGCGGTGAAAAAAGTGATGGATGATGTGACCTTTAAAGAAAACAGCCGCAAAGTGGGCGAAAGCCTGCGCAACGCGGGCGGCTATCAGCGCGCGGTGGAAGAAATTTTTAAACTGAAAATGAAACCGTATGTGAAAATTAAAGGATCCGGATCTGGAGGATCTGGCGTAACCACCTTATCAGGTTTATCAGGTGAGCAAGGTCCGTCCGGTGATATGACAACTGAAGAAGATAGTGCTACCCATATTAAATTCTCAAAACGTGATGAGGACGGCCGTGAGTTAGCTGGTGCAACTATGGAGTTGCGTGATTCATCTGGTAAAACTATTAGTACATGGATTTCAGATGGACATGTGAAGGATTTCTACCTGTATCCAGGAAAATATACATTTGTCGAAACCGCAGCACCAGACGGTTATGAGGTAGCAACTCCTATTGAATTTACAGTTAATGAGGACGGTCAGGTTACTGTAGATGGCGAAGCAACTGAAGGTGACGCTCATACTTAAGGATCCGCTGCAGCCGCAGCAGCAGCTGCCGCAGCC**GAAAATCTGTATTTTCAGGGTGCCATTAAACTGGTTCAGAGCCCGAATGGTAATTTTGCAGCAAGTTTTGTTCTGGATGGCACCAAATGGATCTTCAAAAGCAAATACTATGACAGCAGCAAAGGTTATTGGGTGGGTATTTATGAAGTGTGGGATCGCAAATAA**

**Protein sequence**

HMRGVPHIVMVDAYKRYKGSGGSGANVLVINFPGEGHINPTLAIVSELIQRGETVVSYCIEDYRKKVEATGAEFRVFENFLSQINIMERVNEGGSPLTMLSHMIEASERIVTQIVEETKEEKYDYLIYDNHFPVGRIIANILHLPSVSSCTTFAVNQYINFHDEQESRQVDEMDPLYQSCLAGMERWNKQYGMKCNSMYDIMNHPGDITIVYTSKEYQPRSEVFDESYKFVGPSIATRKEVGSFPTEDLKNEKVIFISMGTVFNEQPALYEKCFEAFKDVDATVVLVVGKKINISQFENIPKNFKLYNYVPQLEVLQHADVFVTHGGMNSSSEALYYGVPLVVIPVTGDQPFVAKRLTEVGAGIRLNRNELTSELLREAVKKVMDDVTFKENSRKVGESLRNAGGYQRAVEEIFKLKMKPYVKIKGSGSGGSGVTTLSGLSGEQGPSGDMTTEEDSATHIKFSKRDEDGRELAGATMELRDSSGKTISTWISDGHVKDFYLYPGKYTFVETAAPDGYEVATPIEFTVNEDGQVTVDGEATEGDAHTGSAAAAAAAAAA**ENLYFQGAIKLVQSPNGNFAASFVLDGTKWIFKSKYYDSSKGYWVGIYEVWDRK**

**>Spy_BacGT**

Vector: pET-28a(+), SpyTag, Linkers, SpyCatcher

Restriction sites: NdeI and BamHI

**DNA sequence:**

CATATGCGTGGTGTACCACATATCGTGATGGTTGATGCGTACAAGCGTTATAAAGGATCTGGAGGATCTGGCGCAAAGGTACTAATGTTTAATTTTCCAGGGGAAGGACATGTGAACCCAACTCTTGCACTTATTGAAGAATTAGTAAAAAGAGGGGAAGAGGTTGTTTATTATTGTGTAGAGGAGTATAAGGAGAAAATTGAAAAAACAGGAGCTTTGTTTCGTCCATATGAAAATTTTTTAGCAAAAGTAGACATGTTGAAAAGGATGAATGGAGAAATAGATCCCTCTGAACTATTATTACATATGGTAAAATCCATGGATAAAATAATAAAGATTGTTATAGAAGAGTTGAAAGAAGAAAAGTATGATTACGTAATATATGATAACAATTTTGCAGTAGGATGGATCATAGCTGAAGCATTACATTTACCTAAAATCTCTTCATGCACAACATTTGCAGTTACGAAAAAGTTATTTAACGCATTGATGAACAATCATAATGAAGGAGACAAAAAATCACCTCTTTATCAAGAAGTTACGCATATTTTAGAAGAGTGGCAAAAGGTATATGGCTTTTCTATGAATGAAAAGAAAAATGTAATGACTTGTGCTGGAAATATTACTATCGTATATACGTCTGAATTTTTCCAACCTGATGTGGAGGAATTGGATGATTCTTATATCTTTGTTGGCCCCTCTATTACATCACGGAAAGATATGCAAGAAATTACTTTTAAACAGAAGGAAGAAGAGAAACTCATTTATATTTCTATGGGAACTGTATTTAATCAACAGATGGATTTTTATTACATATGTTTTGAAGCATTTAAAAATTTTCCTGCTACCATCATATTGTCCGTAGGAAAACATATTGACATCAACCAATTGAAAAATATCCCATCTAATTTCAAAGTTTATAATTATGTGCCTCAGTTAGAGGTATTGAAACAAGCTAATCTTTTTATTACGCATGGGGGAATGAATAGTTCTAGTGAAAGCTTATATTTTGGCGTTCCTATGATCGTTATCCCAGTTATGGGAGATCAACCGATAGTGGCCCAAAGAATAGAAGATTTAAAAGCTGGCATACAATTAAATCTAAAAAAGCTTACTCCAGTGACATTATATAATGCAGTAATGGAAGTATTATCGAATGATGTATACTTAGAAAATAGTCATAAAATCAAATGTACTTTAAAAAATGCTGGTGGATATATTAAAGCCGCTGAAACAATAGAACTATTTAAGTTGAAGACGGGTATTAAAGGATCCGGATCTGGAGGATCTGGCGTAACCACCTTATCAGGTTTATCAGGTGAGCAAGGTCCGTCCGGTGATATGACAACTGAAGAAGATAGTGCTACCCATATTAAATTCTCAAAACGTGATGAGGACGGCCGTGAGTTAGCTGGTGCAACTATGGAGTTGCGTGATTCATCTGGTAAAACTATTAGTACATGGATTTCAGATGGACATGTGAAGGATTTCTACCTGTATCCAGGAAAATATACATTTGTCGAAACCGCAGCACCAGACGGTTATGAGGTAGCAACTCCTATTGAATTTACAGTTAATGAGGACGGTCAGGTTACTGTAGATGGCGAAGCAACTGAAGGTGACGCTCATACTTAA

**Protein sequence**

HMRGVPHIVMVDAYKRYKGSGGSGAKVLMFNFPGEGHVNPTLALIEELVKRGEEVVYYCVEEYKEKIEKTGALFRPYENFLAKVDMLKRMNGEIDPSELLLHMVKSMDKIIKIVIEELKEEKYDYVIYDNNFAVGWIIAEALHLPKISSCTTFAVTKKLFNALMNNHNEGDKKSPLYQEVTHILEEWQKVYGFSMNEKKNVMTCAGNITIVYTSEFFQPDVEELDDSYIFVGPSITSRKDMQEITFKQKEEEKLIYISMGTVFNQQMDFYYICFEAFKNFPATIILSVGKHIDINQLKNIPSNFKVYNYVPQLEVLKQANLFITHGGMNSSSESLYFGVPMIVIPVMGDQPIVAQRIEDLKAGIQLNLKKLTPVTLYNAVMEVLSNDVYLENSHKIKCTLKNAGGYIKAAETIELFKLKTGIKGSGSGGSGVTTLSGLSGEQGPSGDMTTEEDSATHIKFSKRDEDGRELAGATMELRDSSGKTISTWISDGHVKDFYLYPGKYTFVETAAPDGYEVATPIEFTVNEDGQVTVDGEATEGDAHT

**>Spy_BacGT_LCI**

Vector: pET-28a(+), SpyTag, Linkers, SpyCatcher, **TEV protease cleavage site**, **LCI**

Restriction sites: NdeI and BamHI

**DNA sequence:**

CATATGCGTGGTGTACCACATATCGTGATGGTTGATGCGTACAAGCGTTATAAAGGATCTGGAGGATCTGGCGCAAAGGTACTAATGTTTAATTTTCCAGGGGAAGGACATGTGAACCCAACTCTTGCACTTATTGAAGAATTAGTAAAAAGAGGGGAAGAGGTTGTTTATTATTGTGTAGAGGAGTATAAGGAGAAAATTGAAAAAACAGGAGCTTTGTTTCGTCCATATGAAAATTTTTTAGCAAAAGTAGACATGTTGAAAAGGATGAATGGAGAAATAGATCCCTCTGAACTATTATTACATATGGTAAAATCCATGGATAAAATAATAAAGATTGTTATAGAAGAGTTGAAAGAAGAAAAGTATGATTACGTAATATATGATAACAATTTTGCAGTAGGATGGATCATAGCTGAAGCATTACATTTACCTAAAATCTCTTCATGCACAACATTTGCAGTTACGAAAAAGTTATTTAACGCATTGATGAACAATCATAATGAAGGAGACAAAAAATCACCTCTTTATCAAGAAGTTACGCATATTTTAGAAGAGTGGCAAAAGGTATATGGCTTTTCTATGAATGAAAAGAAAAATGTAATGACTTGTGCTGGAAATATTACTATCGTATATACGTCTGAATTTTTCCAACCTGATGTGGAGGAATTGGATGATTCTTATATCTTTGTTGGCCCCTCTATTACATCACGGAAAGATATGCAAGAAATTACTTTTAAACAGAAGGAAGAAGAGAAACTCATTTATATTTCTATGGGAACTGTATTTAATCAACAGATGGATTTTTATTACATATGTTTTGAAGCATTTAAAAATTTTCCTGCTACCATCATATTGTCCGTAGGAAAACATATTGACATCAACCAATTGAAAAATATCCCATCTAATTTCAAAGTTTATAATTATGTGCCTCAGTTAGAGGTATTGAAACAAGCTAATCTTTTTATTACGCATGGGGGAATGAATAGTTCTAGTGAAAGCTTATATTTTGGCGTTCCTATGATCGTTATCCCAGTTATGGGAGATCAACCGATAGTGGCCCAAAGAATAGAAGATTTAAAAGCTGGCATACAATTAAATCTAAAAAAGCTTACTCCAGTGACATTATATAATGCAGTAATGGAAGTATTATCGAATGATGTATACTTAGAAAATAGTCATAAAATCAAATGTACTTTAAAAAATGCTGGTGGATATATTAAAGCCGCTGAAACAATAGAACTATTTAAGTTGAAGACGGGTATTAAAGGATCCGGATCTGGAGGATCTGGCGTAACCACCTTATCAGGTTTATCAGGTGAGCAAGGTCCGTCCGGTGATATGACAACTGAAGAAGATAGTGCTACCCATATTAAATTCTCAAAACGTGATGAGGACGGCCGTGAGTTAGCTGGTGCAACTATGGAGTTGCGTGATTCATCTGGTAAAACTATTAGTACATGGATTTCAGATGGACATGTGAAGGATTTCTACCTGTATCCAGGAAAATATACATTTGTCGAAACCGCAGCACCAGACGGTTATGAGGTAGCAACTCCTATTGAATTTACAGTTAATGAGGACGGTCAGGTTACTGTAGATGGCGAAGCAACTGAAGGTGACGCTCATACTTAAGGATCCGCTGCAGCCGCAGCAGCAGCTGCCGCAGCC**GAAAATCTGTATTTTCAGGGTGCCATTAAACTGGTTCAGAGCCCGAATGGTAATTTTGCAGCAAGTTTTGTTCTGGATGGCACCAAATGGATCTTCAAAAGCAAATACTATGACAGCAGCAAAGGTTATTGGGTGGGTATTTATGAAGTGTGGGATCGCAAATAA**

**Protein sequence**

HMRGVPHIVMVDAYKRYKGSGGSGAKVLMFNFPGEGHVNPTLALIEELVKRGEEVVYYCVEEYKEKIEKTGALFRPYENFLAKVDMLKRMNGEIDPSELLLHMVKSMDKIIKIVIEELKEEKYDYVIYDNNFAVGWIIAEALHLPKISSCTTFAVTKKLFNALMNNHNEGDKKSPLYQEVTHILEEWQKVYGFSMNEKKNVMTCAGNITIVYTSEFFQPDVEELDDSYIFVGPSITSRKDMQEITFKQKEEEKLIYISMGTVFNQQMDFYYICFEAFKNFPATIILSVGKHIDINQLKNIPSNFKVYNYVPQLEVLKQANLFITHGGMNSSSESLYFGVPMIVIPVMGDQPIVAQRIEDLKAGIQLNLKKLTPVTLYNAVMEVLSNDVYLENSHKIKCTLKNAGGYIKAAETIELFKLKTGIKGSGSGGSGVTTLSGLSGEQGPSGDMTTEEDSATHIKFSKRDEDGRELAGATMELRDSSGKTISTWISDGHVKDFYLYPGKYTFVETAAPDGYEVATPIEFTVNEDGQVTVDGEATEGDAHTGSAAAAAAAAAA**ENLYFQGAIKLVQSPNGNFAASFVLDGTKWIFKSKYYDSSKGYWVGIYEVWDRK**

**>Spy_BarGT-1_LCI**

Vector: pET-28a(+), SpyTag, Linkers, SpyCatcher, **TEV protease cleavage site**, **LCI**

Restriction sites: NdeI and BamHI

**DNA sequence:**

CATATGCGTGGTGTACCACATATCGTGATGGTTGATGCGTACAAGCGTTATAAAGGATCTGGAGGATCTGGCCTGAACATTCTGGTGGTGAACTTTCCGGCGGAAGGCCATGTGAACCCGACCCTGAACCTGGTGAAAGCGTTTACCAAACGCGGCGATAACGTGCATTATATTACCACCGCGAACTTTAAAGATCGCATTGAAGATCTGGGCGCGACCGTGCATATTCATCCGGATCTGCTGAAGGAAATTAGCATTGATGCGGAAACGAGCAGCGGCCTGAACGCGTTTTTTCATGTGCATGTGCAGACGAGCCTGTATATTCTGGAAATTACCAAACAGCTGTGCGAAAGCATTAACTTTGATTTTGTGATTTATGATATTTTTGGCGCGGGCGAACTGGTGAAAGAATATCTGCAAGTGCCGGGCGTGGTGAGCAGCCCGATTTTTCTGATTCCGCCGGAATTTCTGAAAACCCTGCCGTTTCATCCGAACGCGGATATGCCGTTTCAGCCGGAAGAAATTAGCGAAAAACTGCTGAATCAGATGGAACATAAATTTGGCGTGAAACCGAAAAACAACCTGCAGTTTATGAACAACAAAGGCGATGTGTGCCTGGTGTATACGAGCCGCTATTTTCAGCCGAACAACGAGAGCTTTGGCGAAAACAACATTTTTATTGGCCCGAGCATTAGCAAACGCAAAACCAACATTAAATTTCCGCTGGAAAGCCTGAAAGAAAAAAAAGTGATTTATATTAGCATGGGCACCCTGCTGGAAGGCCTGGAACCGTTTTTTAACACCTGCATTGATACCTTTAGCGATTTTGATGGCATTGTGGTGATGGCGATTGGCGATCGCAACGATATTAGCAAAATTAAAAAAGCGCCGGATAACTTTATTATTGCGCCGTATGTGCCGCAGAGCGAAATTCTGAACGAAGCGGATGTGTTTATTACCCATGGCGGCATGAACAGCGTGCATGATGCGATTCATTATAACGTGCCGTTTGTGATTATTCCGCATGATAAAGATCAGCCGATGATTGCGCAGCGCCTGACCGAACTGGAAGCGGCGCATCGCCTGCTGAAAGAACATGTGAACGTGCATACCCTGAAAGAAGCGGTGACCGATGTGCTGAGCAACGAAAAATATAAACATGGCATTCGCAAACTGAACGATAGCTTTCTGGAATGCGGCGGCAGCAAAGAAGCGATTGCGGTGATTGAAAGCCTGCTGAACAAAGTGAAACTGGGATCCGGATCTGGAGGATCTGGCGTAACCACCTTATCAGGTTTATCAGGTGAGCAAGGTCCGTCCGGTGATATGACAACTGAAGAAGATAGTGCTACCCATATTAAATTCTCAAAACGTGATGAGGACGGCCGTGAGTTAGCTGGTGCAACTATGGAGTTGCGTGATTCATCTGGTAAAACTATTAGTACATGGATTTCAGATGGACATGTGAAGGATTTCTACCTGTATCCAGGAAAATATACATTTGTCGAAACCGCAGCACCAGACGGTTATGAGGTAGCAACTCCTATTGAATTTACAGTTAATGAGGACGGTCAGGTTACTGTAGATGGCGAAGCAACTGAAGGTGACGCTCATACTTAAGGATCCGCTGCAGCCGCAGCAGCAGCTGCCGCAGCC**GAAAATCTGTATTTTCAGGGTGCCATTAAACTGGTTCAGAGCCCGAATGGTAATTTTGCAGCAAGTTTTGTTCTGGATGGCACCAAATGGATCTTCAAAAGCAAATACTATGACAGCAGCAAAGGTTATTGGGTGGGTATTTATGAAGTGTGGGATCGCAAATAA**

**Protein sequence**

HMRGVPHIVMVDAYKRYKGSGGSGLNILVVNFPAEGHVNPTLNLVKAFTKRGDNVHYITTANFKDRIEDLGATVHIHPDLLKEISIDAETSSGLNAFFHVHVQTSLYILEITKQLCESINFDFVIYDIFGAGELVKEYLQVPGVVSSPIFLIPPEFLKTLPFHPNADMPFQPEEISEKLLNQMEHKFGVKPKNNLQFMNNKGDVCLVYTSRYFQPNNESFGENNIFIGPSISKRKTNIKFPLESLKEKKVIYISMGTLLEGLEPFFNTCIDTFSDFDGIVVMAIGDRNDISKIKKAPDNFIIAPYVPQSEILNEADVFITHGGMNSVHDAIHYNVPFVIIPHDKDQPMIAQRLTELEAAHRLLKEHVNVHTLKEAVTDVLSNEKYKHGIRKLNDSFLECGGSKEAIAVIESLLNKVKLGSGSGGSGVTTLSGLSGEQGPSGDMTTEEDSATHIKFSKRDEDGRELAGATMELRDSSGKTISTWISDGHVKDFYLYPGKYTFVETAAPDGYEVATPIEFTVNEDGQVTVDGEATEGDAHTGSAAAAAAAAAA**ENLYFQGAIKLVQSPNGNFAASFVLDGTKWIFKSKYYDSSKGYWVGIYEVWDRK**

# 5. References

[1] K. Katoh, J. Rozewicki, K. D. Yamada, *Briefings in bioinformatics* **2019**, *20*, 1160-1166.

[2] F. Ronquist, M. Teslenko, P. Van Der Mark, D. L. Ayres, A. Darling, S. Höhna, B. Larget, L. Liu, M. A. Suchard, J. P. Huelsenbeck, *Systematic biology* **2012**, *61*, 539-542.

[3] I. Letunic, P. Bork, *Nucleic acids research* **2021**, *49*, W293-W296.

[4] P. Zhang, Y. Ji, S. Meng, Z. Li, D. Hirtz, L. Elling, U. Schwaneberg, *Green Chemistry* **2023**.

[5] X. Wang, Y. Zhang, W. Luo, A. A. Elzatahry, X. Cheng, A. Alghamdi, A. M. Abdullah, Y. Deng, D. Zhao, *Chemistry of Materials* **2016**, *28*, 2356-2362.
